# Patient-specific functional networks track early cognitive alterations and minor hallucinations in Parkinson’s disease

**DOI:** 10.1038/s44220-026-00657-x

**Published:** 2026-06-08

**Authors:** Sara Stampacchia, Fosco Bernasconi, Dimitri Van De Ville, Enrico Amico, Lada Kohoutová, John-Paul Taylor, Alan Thomas, Javier Pagonabarraga, Jaime Kulisevsky, Olaf Blanke

**Affiliations:** 1 Laboratory of Cognitive Neuroscience (LNCO), Neuro-X Institute and Brain Mind Institute, École Polytechnique Fédérale de Lausanne (EPFL), Lausanne, Switzerland; 2 Neuro X Institute, École Polytechnique Fédérale de Lausanne (EPFL), Lausanne, Switzerland; 3 Department of Radiology and Medical Informatics, University of Geneva, Geneva, Switzerland; 4 School of Mathematics, University of Birmingham, Birmingham, UK; 5 Centre for Human Brain Health, University of Birmingham, Birmingham, UK; 6 Translational and Clinical Research Institute, Newcastle University, Newcastle, UK; 7 Movement Disorders Unit, Neurology Department, Sant Pau Hospital, Barcelona, Spain; 8 Universitat Autònoma de Barcelona (UAB), Barcelona, Spain; 9 Centro de Investigación en Red-Enfermedades Neurodegenerativas (CIBERNED), Madrid, Spain; 10 Biomedical Research Institute (IIB-Sant Pau), Barcelona, Spain; 11 Department of Clinical Neurosciences, Geneva University Hospital, Geneva, Switzerland

**Keywords:** Parkinson's disease, Network models

## Abstract

Minor hallucinations (MHs) affect up to 40% of patients with Parkinson’s disease (PD) and are early indicators of cognitive decline. Unlike previous group-level studies, connectome-based brain fingerprinting enables the capture of individualized neural features. Leveraging this framework in a cross-sectional cohort of patients with Parkinson’s disease with and without MHs using resting-state functional magnetic resonance imaging, here we show that each patient exhibits a unique brain fingerprint, irrespective of MH status. The most individualized features were associated with clinically relevant measures such as motor-symptom severity and cognitive performance. Patients with MHs showed a loss of individual-specific features in brain networks linked to cognitive health, whereas the cerebellar-to-cortex connectivity—typically less distinctive—became more prominent, emphasizing the role of somatosensory and motor integration in MH pathogenesis. Moreover, MH-related fingerprint regions mapped onto cortical territories with lower densities of neurotransmitter systems implicated in hallucinations. Overall, our fully data-driven findings reveal a distinct, patient-specific signature that differentiates patients with Parkinson’s disease and MHs.

## Abstract (web summary)

In this study, the authors investigated minor hallucinations in patients with Parkinson’s disease using a connectome-based brain fingerprinting approach and examined their association with cognitive decline

## Main

Parkinson’s disease (PD) is an irreversible, progressive neurodegenerative disease characterized by Lewy bodies (misfolded α-synuclein)^1^, affecting the brainstem, nigrostriatal dopaminergic neurons and other subcortical and cortical structures^2^. Although primarily a movement disorder, PD is characterized by early non-motor symptoms, including hallucinations^3,4^, which are highly prevalent^5–8^, affecting up to 50% of patients regularly^9–11^ and with a prevalence of up to 70% in the advanced stages^12,13^. Hallucinations are linked to faster cognitive decline, dementia^9,12,14–17^, earlier home placement^18–20^ and increased mortality risk^18,20^.

Most studies on the neural substrates of hallucinations in PD have focused on complex visual hallucinations, the most common type in advanced PD^14^. Activation studies suggest altered bottom–up and top–down visual processing in visual hallucinations, with reduced activation in primary visual cortices and increased activation in fronto-parietal regions during visual stimulation^21^ as well as ongoing hallucinatory episodes^22^. Similarly, resting-state functional connectivity studies reveal convergent findings, with increased connectivity between sensory and default-mode regions, consistent with an overreliance on top–down signals (recently reviewed in ref. ^23^). In line with these results, the attentional network hypothesis^24–26^ proposes that hallucinations in PD arise from impaired recruitment of the dorsal attention network (DAN) in response to ambiguous visual input, itself a consequence of abnormal bottom–up processing in early visual areas^27^. This failure of stimulus-driven control may bias perception towards internally driven interpretations, mediated by default mode (DMN) and/or salience (SAL) networks.

Although the majority of past imaging work has focused on complex visual hallucinations in PD, so-called minor hallucinations (MHs)—which include presence hallucinations, passage hallucinations and pareidolias^11,28^—are of clinical relevance because they often occur at the earlier stages of PD^7,10^, may even precede Parkinsonian motor symptoms^29^ and occur in up to 40% of patients^9,30^. Brain alterations in MHs partially overlap with those observed in complex visual hallucinations and are consistent with the attentional network hypothesis. Recent work revealed increased activation in the posterior middle-temporal gyrus, a hub at the intersection of the DMN, SAL and DAN, and inferior frontal gyrus (part of the DMN) during robotically induced MHs^31^ as well as increased frontal EEG activity during rest^9^. Structurally, MHs are associated with gray-matter reduction in visuo-temporal regions^32,33^. These studies have linked these brain alterations with mild cognitive impairment. Resting-state connectivity studies have reported increased connectivity within the DMN^34,35^, reduced DAN connectivity and disrupted interactions between the DAN and the DMN^34^. Although this body of research proposes a convergent account about the neural mechanisms of MHs and visual hallucinations in PD, it has primarily relied on group-averaged analyses, which do not capture the patient-specific nature of functional brain architecture (also known as the brain fingerprint)^36,37^. Considering the heterogeneity and individually distinct phenomenological expression of hallucinations, accounting for neural variability is particularly crucial.

Functional connectivity patterns estimated from resting-state functional magnetic resonance imaging (fMRI) or magnetoencephalography (MEG) data—referred to as functional connectomes (FCs)—are unique to each individual^36–38^. Accordingly, these have been called ‘brain fingerprints’ as they allow for the identification of individuals from a population, that is, the correct matching of an individual to their own FC, leveraging individual differences across individuals. In this article we therefore refer to this property of FCs as brain fingerprinting or identifiability interchangeably. These unique features of FCs have been linked to cognitive traits^39,40^ and are altered in psychiatric^41–43^ as well as neurodegenerative diseases, including Alzheimer’s disease^44,45^ and PD^46,47^. In a MEG study, Troisi Lopez and colleagues^47^ reported decreased identifiability in PD versus healthy controls (HCs) predicting clinical motor impairment, whereas da Silva Castanheira and colleagues^46^ replicated and extended these findings by showing that identifiability remained high in patients with PD relative to HCs only when focusing on rhythmic activity, predicting the laterality of the symptoms. This indicates that some aspects of patient-specific connectivity may be preserved in PD, depending on the neural signal components examined. Building on these insights, the present study investigates patient-specific connectivity using fMRI, leveraging its higher spatial resolution, to examine whether fMRI connectome-based fingerprints can differentiate patients with PD and, in particular, those with MHs (PD-MH) or without any type of hallucinations (PD-nH).

If FCs are unique to an individual, they may also encode person-specific features that capture clinically meaningful variability. This exploratory study aims to determine the extent to which group-level alterations previously reported in patients with PD-MH reflect consistent patterns across individuals versus idiosyncratic effects. To this end, we investigate whether connectome-based fingerprints derived from fMRI can identify patient-specific functional networks in PD similarly to HCs and whether there are patterns unique to patients with PD-MH relative to those with PD-nH. We further examine whether these person-specific alterations are associated with clinical traits, thereby testing whether individual neural signatures correspond to clinically relevant variability. Given that hallucinations represent a major risk factor for dementia^12^, we hypothesise that patient-specific network alterations in PD-MH are associated with early signs of cognitive impairment. Finally, we investigate whether brain regions with high interindividual neural variability are associated with the cortical density of major neurotransmitters systems implicated with hallucinations. Previous studies have reported alterations in cholinergic, serotonergic, dopaminergic and GABAergic systems in patients with PD and hallucinations^48–50^. Understanding whether interindividual variability in functional connectivity reflects the cortical distribution of specific neurotransmitter systems is of considerable interest as it could reveal the neurochemical underpinnings of individual differences in brain organization.

## Results

**Fig. 1:**
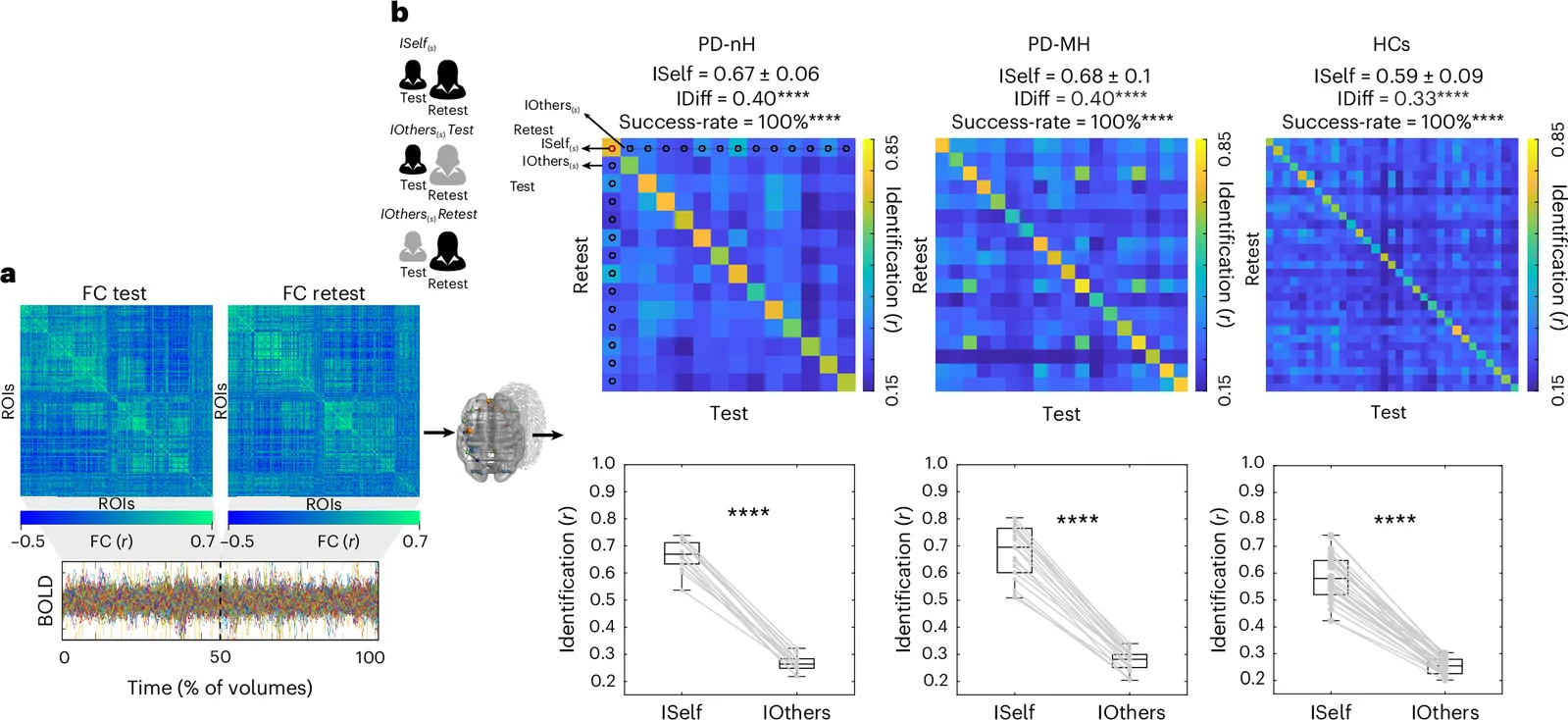
Functional connectivity variability within and between individuals. **a**, Functional connectome estimation at test (first half of the volumes) and retest (second half of the volumes). ROIs, regions of interest. BOLD, blood-oxygenation-level-dependent. **b**, Identifiability matrices (top) show ISelf and IOthers test–retest reliability, determined as the Pearson’s correlation coefficient (*r*), in patients with PD-nH (*n* = 14; left) and PD-MH (*n* = 18; middle) as well as HCs (*n* = 31; right). Each data point represents the similarity/dissimilarity of one individual across separate measurements (test versus retest). The ISelf and IOthers values of individuals are represented in the diagonal and off-diagonal, respectively, elements of the matrix. The average ISelf (mean ± s.d.), IDiff and Success-rate were similar in the three groups, and IDiff and Success-rate differed at *P* < 0.00001 from random distributions. Box plots show that ISelf was significantly higher than IOthers in all three groups (paired-sample two-sided *t*-test; PD-MH, *P* = 1.63 × 10⁻^14^; PD-nH, *P* = 3.79 × 10⁻^13^; HCs; *P* = 1.97 × 10⁻^22^; bottom). Box plots display the median (central line), interquartile range (box edges) and 1.5× the interquartile range (whiskers); the gray lines connect individual data points across metrics. ****P < 0.0001. Credit: ‘Person’ icons (https://thenounproject.com/icon/person-4851862/, https://thenounproject.com/icon/person-4851854/) by Wilson Joseph from Noun Project (CC BY 3.0).

**Fig. 2:**
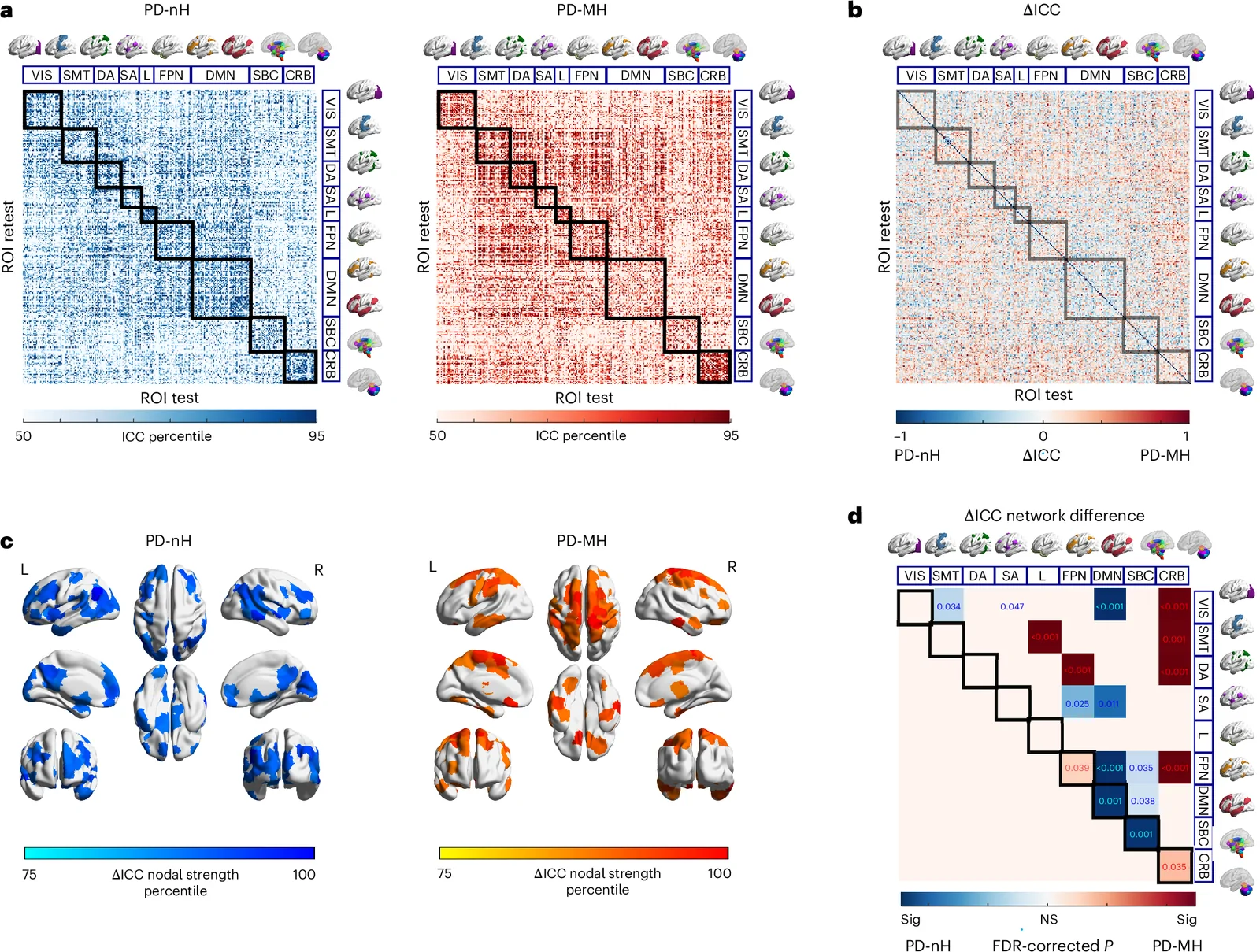
Functional connectivity patterns making individuals identifiable differ between patients with Parkinson’s disease with and without minor hallucinations. **a**, Edgewise ICC matrices for patients with PD-nH (*n* = 14; left) and PD-MH (*n* = 18; right). The ICC quantifies the within-individual similarity between test and retest for each edge (FC between two regions). Here we show a different configuration of the edges with highest ICC in PD-nH versus PD-MH. We display edges with ICCs between the 50th and the 95th percentile. **b**, Minor hallucination-related differential ICC, that is, ΔICC matrix showing the differential ICC between PD-MH and PD-nH. **a**,**b**, Data are ordered according the seven RSNs, and the subcortical and cerebellar regions. **c**, Brain renders showing the nodal strength of each group ICC map, masked for the ΔICC value for the PD-nH and PD-MH groups. Nodal strength was computed as the average of edge weights for each ROI. Only the nodes of the >75th percentiles are displayed here. L, left; R, right. **d**, Matrix illustrating network pairs that show significant differences in the proportion of high ΔICC edges (that is, MH-related signature) between the two groups. High ΔICC edges are defined as those exceeding the 97th percentile within each group (approximately corresponding to an ΔICC of ±0.5, where ICC = 0.5 is typically interpreted as ‘fair’)^84^. Results are presented for all seven RSNs as well as the subcortical and cerebellar regions, including both within-network and between-network connections. Network-pair comparisons were performed using a two-sided binomial test, testing whether the number of edges where ΔICC was higher in PD-MH exceeded what would be expected by chance (null hypothesis: *P* = 0.5). *P* values were corrected for multiple comparisons across all network pairs using the Benjamini–Hochberg FDR procedure. Colored cells indicate network pairs with significant group differences, with the FDR-corrected *P* value reported; NS, *P* > 0.05; sig, significant (*P* < 0.05). Colors reflect the directionality of the effect, with blue indicating a higher proportion of high-ΔICC edges in PD-nH and red indicating a higher proportion in PD-MH. DA, DAN; L, limbic network; SA, SAL.

**Fig. 3:**
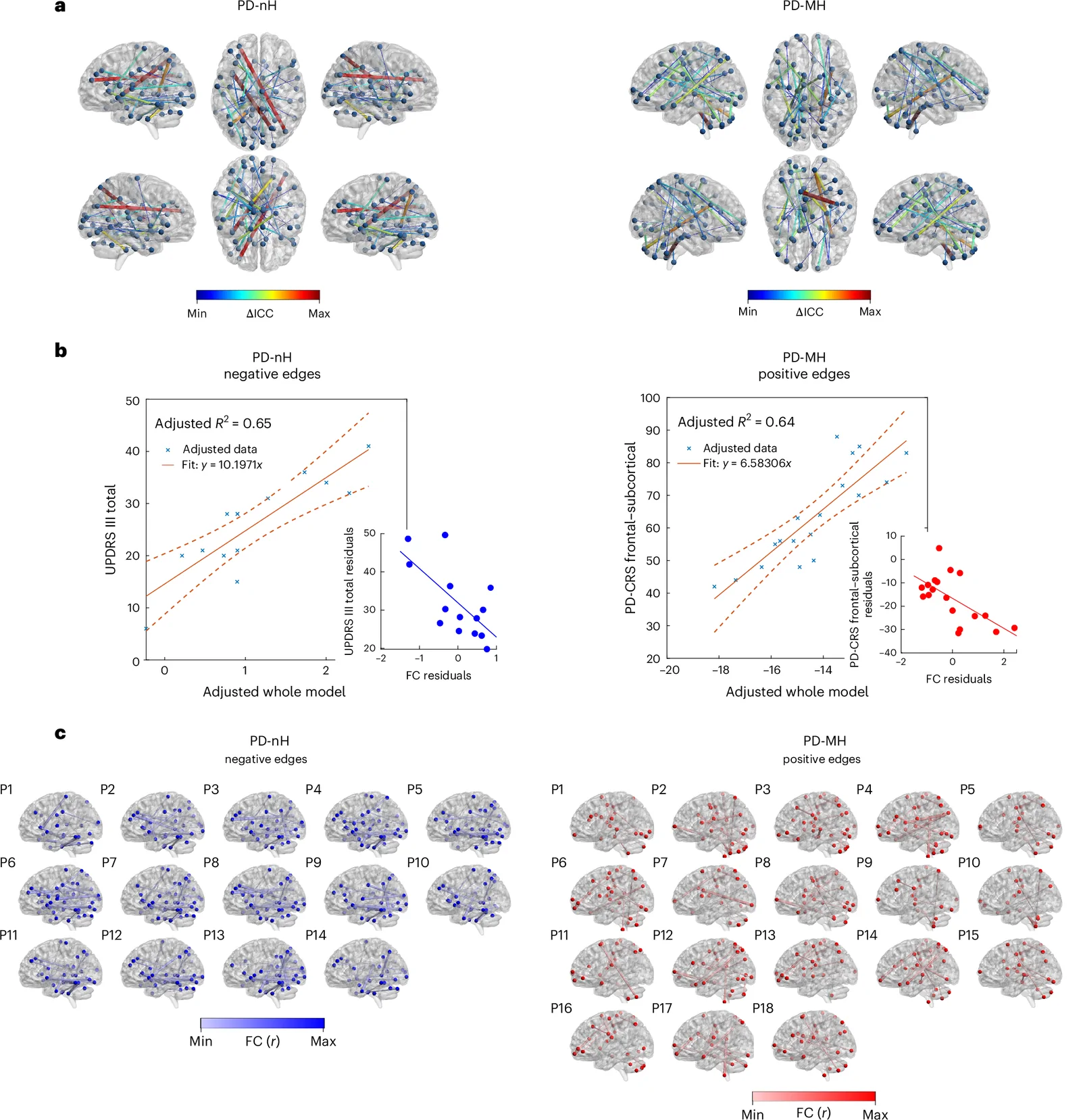
Functional connections with a high fingerprint associate with clinical scores. **a**, Brain renders showing the top-0.1% ΔICC edges for the two PD groups. The top ΔICC matrices were used as masks to isolate the functional connections that maximize differentiability in fingerprint between the two groups in each FC. **b**, Linear regression models showing that clinical scores were significantly associated with functional connectivity in the top ΔICC edges (clinical score = FC_top ΔICC group_ + age + years of education + disease duration). The dashed lines represent the 95% confidence intervals around the fitted regression line. Insets: correlation between residuals of the clinical score and functional connectivity to show directionality of the association. For UPDRS-III total: higher scores = greater motor impariment; for PD-CRS frontal-subcortical: lower scores = worse cognition. For functional connectivity: positive values indicate greater connectivity and negative values indicate greater disconnectivity. **c**, Brain renders of individual patients showing the functional connections with high fingerprint associated with clinical score. Each render displays, for every participant (PD-nH P1–14 and PD-MH P1–18), the FC strength of the top-0.1% ΔICC edges identified at the group level (**a**). The group-derived ΔICC mask was applied to the FC of each study participant and the FC values within these edges were extracted (and averaged for the score in **b**). Positive and negative ΔICC edges were treated separately to avoid cancellation effects when computing network strength. As this separation is performed at the individual level, the subset of expressed edges (and corresponding nodes) may vary across participants, although their spatial distribution largely overlaps. The color intensity reflects the patient-specific expression of the MH-related fingerprint. Max, maximum; min, minimum.

**Fig. 4:**
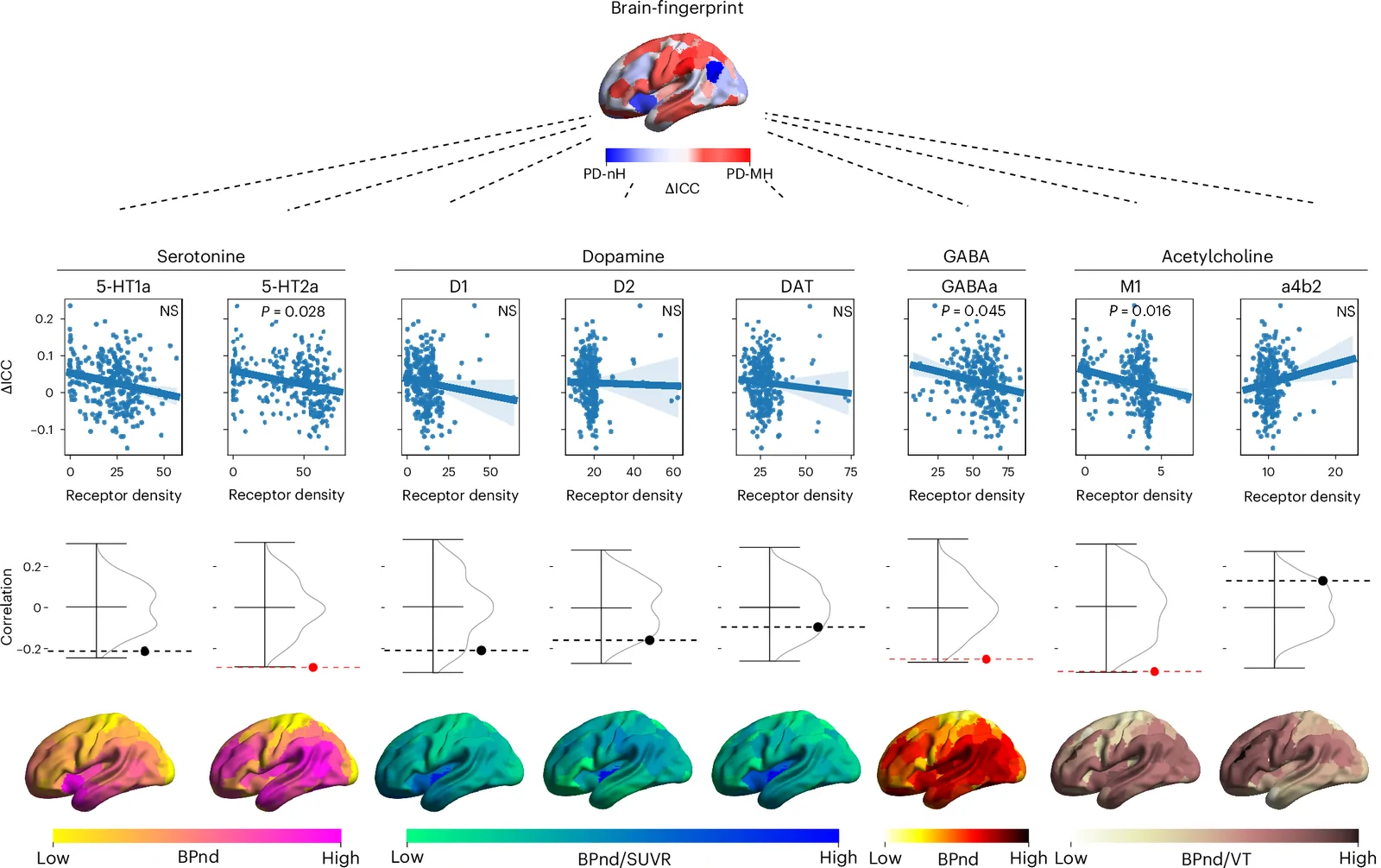
Association between minor hallucination-related brain fingerprint and neurotransmitter receptor density. Scatterplots showing Spearman spatial correlations between the ΔICC nodal strength map (where higher values indicate connections with an MH-related brain fingerprint) and cortical neurotransmitter receptor densities for dopamine, serotonin, GABA and acetylcholine. Each correlation was computed across 278 cortical regions (parcels), yielding a spatial correlation value for each receptor map. Violin plots depict the observed Spearman correlation (red or black dot with dashed line) relative to a null distribution of correlations obtained by computing Spearman correlations between the ΔICC map and 1,000 spatially permuted surrogate receptor maps (Moran spectral randomization), which accounts for spatial autocorrelation. The whiskers represent the minimum and maximum values of the null distribution and the horizontal line indicates the median. Statistical significance was assessed using a two-sided permutation test; the reported *P* values were corrected for multiple comparisons using the Benjamini–Hochberg FDR procedure. Red dots indicate that the observed correlation exceeds the surrogate distribution at *P* < 0.05; NS, not significant (*P* > 0.05). Serotonin receptors include 5-HT1a and 5-HT2a; dopamine receptors include D1 and D2, and DAT is the dopamine transporter; GABAa is the GABA receptor; and acetylcholine receptors include M1 (muscarinic) and α4β2 (nicotinic). BPnd, binding potential; SUVR, standardized uptake value ratio; VT, free-fraction-corrected distribution volume.

Our approach (Methods) involved the following steps. We first estimated the FCs for each study participant in the first and the second half of the fMRI acquisition^45^ (Fig. 1a). We then assessed derived metrics such as ISelf (self-similarity), IOthers (similarity to others), IDiff (brain discriminability) and Success-rate^36^ separately for patients with PD-MH and PD-nH as well as HCs using identifiability matrices^37^ (Fig. 1b). Next, we analysed spatial specificity by computing individual FC-edge distinctiveness using intraclass correlation coefficients (ICCs), mapping brain fingerprints across functional networks and onto the cortical surface (Fig. 2). We then tested whether functional connections with high fingerprinting capability would differ between PD-MH and PD-nH, and whether they would associate with clinical scores (Fig. 3). Finally, we explored the association between the cortical topography of brain fingerprints and neurotransmitters systems implicated in hallucinations (Fig. 4).

### Whole-brain within-session fingerprints

Investigating the whole-brain fingerprints, we observed a 100% Success-rate in the PD-MH, PD-nH and HC groups, indicating that identifiability is preserved in the presence of PD. In other words, as in healthy individuals, each patient could be correctly identified based solely on their FC (Fig. 1b; ISelf > IOthers, *P* < 0.001). The IDiff was equally high in the PD-MH and PD-nH groups (0.40), indicating strong individual brain discriminability in the patient groups. The IDiff was slightly lower in HCs (0.33), although differences in magnitude between individuals with PD and HCs should be interpreted with caution given the potential variability in signal-to-noise ratio across acquisition sites. The test–retest reliability (ISelf) was high in the three groups, with no significant group differences after controlling for nuisance variables (analysis of variance (ANOVA), 5,000 permutations; *F*(2,53) = 0.1, *P* = 0.605; mean ± s.d.: PD-nH, 0.67 ± 0.06; PD-MH, 0.68 ± 0.1; HCs, 0.59 ± 0.08; Fig. 1b). Similarly, no group differences were observed in the between-individual similarity (IOthers; ANOVA, 5,000 permutations; *F*(2,53) = 0.70, *P* = 0.248; mean ± s.d.: PD-nH, 0.27 ± 0.03; PD-MH, 0.27 ± 0.04; HCs, 0.25 ± 0.03). Although no group differences were observed, some covariates (for example, age and framewise displacement (FD)) were significantly associated with IOthers (full statistics and additional analyses in Supplementary Table 1). This covariate effect was primarily driven by the HC group rather than the patients with PD, as confirmed by supplementary analyses (compare with Supplementary Information). Permutation testing confirmed that IDiff and Success-rate significantly deviated from null distributions (*P* < 0.0001) in both PD subtypes and HC alike (Fig. 1b). In summary, these findings demonstrate that fingerprinting can be applied in PD: patients can be individually identified with accuracy comparable to HCs based solely on within-scan brain connectivity patterns, independent of clinical status and significantly above surrogate null models.

### Spatial specificity of brain fingerprints of minor hallucinations in Parkinson’s disease

Analysing the local properties of the brain fingerprints using edgewise ICC^37^, we observed that the functional connections with the highest fingerprint (that is, the most reliable within groups) exhibited different spatial distributions in the PD-nH, PD-MH and HC groups (Fig. 2a and Supplementary Fig. 1). To identify ICC edges that differed between patients with and without MHs—that is, features related to MHs rather than general PD characteristics—we computed an edge-based differential identifiability (ΔICC) matrix (that is, ICC_PD-MH _− ICC_PD-nH_; Fig. 2b). To test whether the features that most strongly differed in PD-MH from PD-nH represented a signature of MHs rather than features generally associated with PD, we compared them with an edgewise differential identifiability matrix contrasting patients with PD (PD-MH + PD-nH) with HCs (that is, ICC_PD_ − ICC_HC_). Only 0.5% of the top ΔICC_PD-MH − PD-nH_ connections had the same direction of effect and exceeded the minimal ΔICC value observed in PD-MH compared with PD-nH (ICC = 0.5), suggesting that the edges with the highest ΔICC_PD-MH − PD-nH_ values are largely related to MHs (Supplementary Fig. 1).

The MH-related differential ICC edges (ΔICC_PD-MH – PD-nH_) were then mapped onto the cortical surface as nodal strength (Fig. 2c) and the highest differences across the seven resting-state networks (RSNs), subcortical and cerebellar regions were identified (Fig. 2d). This analysis revealed differences between PD-MH and PD-nH. A higher proportion of high ΔICC edges primarily involving the fronto-parietal network (FPN), including its connections with the DAN network and the cerebellum (CRB), as well as within-FPN networks connections was observed for the PD-MH group. Additional differences were observed in several cerebellar pairs (CRB–CBR; CRB–DAN and CRB with visual network, VIS) and networks connected with the somatomotor network (SMT; Limbic–SMT and CRB–SMT).

In contrast, PD-nH showed a higher proportion of high-ΔICC edges primarily involving the DMN network, including its connection with the SAL and VIS, as well as within-network DMN connections. This was followed by the subcortical network (SBC) pairs, specifically SBC–FPN and SBC–DMN. Additional differences were observed in SAL connections (SAL–VIS and SA–FPN) and SMT–VIS connections (Fig. 2d). Supplementary Fig. 2 shows that these patterns are largely maintained across different ΔICC thresholds (no threshold and 97th, 98th and 99th percentiles). In summary, these findings indicate that brain fingerprint is expressed through distinct functional connections in patients with PD with (PD-MH) and without MHs (PD-nH).

### Functional connections with a high fingerprint are associated with clinical scores

In this analysis we aimed to explore whether the functional connections that maximally differed in brain-fingerprint features between PD-nH and PD-MH were associated with clinical scores in the two groups. To identify ICC edges that maximally differed between the two groups, we selected the top-0.1% ΔICC edges separately in the PD-MH and PD-nH groups. Their spatial distribution on the cortical surface is shown for both groups separately in Fig. 3a. These top ΔICC edges were then used to isolate functional connections with the highest edgewise differential identifiability in the FC of each individual, from which we derived a sum score separately for positive and negative connections. Using linear models, we tested whether this functional connectivity score was related to interindividual variability in clinical scores of motor-symptom severity (using the Unified Parkinson’s Disease Rating Scale (UPDRS) III total score), frontal–subcortical executive functioning (using the PD-cognitive rating scale (PD-CRS) frontal–subcortical score) and posterior–cortical functions (using the PD-CRS posterior score) separately in each group.

In patients with PD-nH, negative connectivity in high-ΔICC edges was significantly associated with UPDRS III total score (adjusted *R*^2^ = 0.65, *P* = 0.003; Fig. 3b), indicating that more significant disconnection of fingerprinting hubs (individual brain renders in Fig. 3c) was associated with greater motor impairment. This differed for patients with PD-MH, in whom functional connectivity was associated with the PD-CRS frontal–subcortical neuropsychological score (adjusted *R*^2^ = 0.64, *P* = 0.010; Fig. 3b), with higher connectivity between fingerprinting hubs (Fig. 3c) linked to greater executive impairment. No significant associations were found for the PD-CRS posterior score in either group, which was consistent with the early disease stage. For transparency, we report both uncorrected and false-detection rate (FDR)-corrected *P* values. After FDR correction, some results were marginally significant (for example, PD-nH, *P* = 0.003, FDR-corrected *P* = 0.031; PD-MH, *P* = 0.010, FDR-corrected *P* = 0.059). Given the exploratory nature of these analyses, we emphasize that they are primarily hypothesis-generating rather than confirmatory, as fingerprinting is a relatively novel approach and we did not pre-register specific edgewise hypotheses.

Notably, neither the top-0.1% edges selected based on *t*-tests nor edges from the full connectome were associated with any of the clinical measures in either group, indicating that ICC-based edge selection identifies functionally relevant connections not captured by standard group-difference statistics nor when using unselected individualized edges.

### Cortical regions with stronger minor hallucination-related fingerprint exhibit reduced serotonin, GABA and acetylcholine receptor density

Given their role in the neurobiology of hallucinations, we examined whether the topography of the MH-related brain fingerprints aligned with the cortical distribution of dopamine, serotonin, GABA and acetylcholine receptors (Fig. 4). After accounting for spatial autocorrelation, we found that connections with a stronger hallucinations-specific brain fingerprint showed significantly lower cortical density of 5-HT2A serotonin (FDR-corrected *P* = 0.028), GABAa (FDR-corrected *P* = 0.045) and M1 acetylcholine (FDR-corrected *P* = 0.016) receptors.

## Discussion

In this study we analysed the features of brain fingerprints using resting-state fMRI in patients with PD-MH and PD-nH. First, we observed that identifiability is preserved in the presence of PD, comparable to HCs, and irrespective of the hallucination status of the patients, with no group differences between PD-MH, PD-nH and HCs in primary fingerprint indices (ISelf and IOthers; Fig. 1). Second, we identified the spatial features of brain fingerprints that maximally differed across the two groups (Fig. 2). Crucially, the spatial features of brain fingerprints that differed most strongly between patients with PD-MH and PD-nH also allowed us to identify patient-specific functional networks that associated with clinical traits at the individual level, differentiating cognitive symptoms in PD-MH from motor symptoms in PD-nH (Fig. 3). Finally, the spatial features of the brain fingerprints were associated with the cortical density of neurotransmitter systems linked to hallucinations, again with distinct patterns in patients with and without MHs (Fig. 4).

Our results demonstrate that individual functional connectivity patterns contain stable, individual-specific features, enabling patient identification in PD. These findings extend previous evidence from MEG studies in PD^46,47^ and align with fMRI studies in other neurodegenerative disorders^45^. Whereas previous MEG studies reported that identifiability in PD may vary depending on the neural signal component considered^46,47^, the present fMRI results show that stable individual-specific connectivity is detectable at the macroscale. This suggests that the preservation of brain fingerprints in PD may reflect broader organizational properties despite potentially different physiological underpinnings. The persistence of brain fingerprinting in neurodegenerative conditions is a nontrivial finding because it shows that even within a homogeneous group—patients with the same diagnosis, same range of biological alterations and symptoms and, in this case, belonging to the same subtype with potentially similar clinical trajectories—each individual remains highly distinct. Features embedded in these FCs therefore hold great promise for advancing personalized prognostics and treatments by providing objective data-driven biomarkers to identify individual differences and track disease progression, and may predict variability in therapeutic responses^51,52^. We note, however, that our cohort included patients with a relatively short disease duration (mean ± s.d. PD-MH: 5.2 ± 3.8 and PD-nH: 4 ± 2 yr, see Table 1). Future studies will be needed to determine whether these stable, individual-specific connectivity patterns persist in more advanced stages of PD.

**Table 1:** Demographics and clinical variables

	PD-MH	PD-nH	HCs	*P* value^a^
*n*	18	14	31	-
Sex (% female participants)	44.4	28.6	32.3	0.652
Age (yr; mean ± s.d.)	70.4 ± 5.5	65.8 ± 7.8	70.9 ± 4.2	0.094
Education (yr; mean ± s.d.)	12.5 ± 4.6	11.6 ± 4.9	13.7 ± 2.69	0.445
Motor symptoms severity: UPDRS III (mean ± s.d.)	21.9 ± 8.6	25.8 ± 9.2	-	0.238
Cognitive functions: PD-CRS frontal maximum = 104	63.3 ± 14.9	64.4 ± 14.8	-	0.848
Cognitive functions: PD-CRS posterior maximum = 30	28.6 ± 1.4	28.6 ± 1.7	-	0.875
Disease duration (yr; mean ± s.d.)	5.2 ± 3.8	4 ± 2	-	0.253
Daily dose of L-dopa (mg)	697.2 ± 311.4	601.1 ± 311.9	-	0.394
Dopamine agonist dose (mg)	143.7 ± 117.9	153.6 ± 139.9	-	0.833

^a^Group differences were assessed using a two-sided Fisher’s exact test for sex distribution, and a one-way ANOVA or its nonparametric equivalent (Kruskal–Wallis test) for three-group comparisons (PD-MH, PD-nH and HCs). Two-group comparisons (PD-MH versus PD-nH) were performed using two-sided independent samples *t*-test or its nonparametric equivalent (Wilcoxon rank-sum test) depending on whether normality assumptions were met (Shapiro–Wilk test). *P* > 0.05, not significant.

In healthy individuals, the most prominent networks allowing identification are the DMN, FPN and SAL^36,37^, a pattern that has also been confirmed in cognitively healthy older adults in previous work^45^ and in the present HC dataset (Supplementary Fig. 1). Here PD-MH, relative to PD-nH, exhibited lower ΔICC in DMN-within-network connections as well as in DMN–FPN, DMN–SAL, DMN–VIS and SAL–FPN edges (Fig. 2d), indicating diminished individual-specific features in these networks. This pattern aligns with the notion that PD-MH have a more severe and rapidly advancing form of PD often associated with cognitive impairments^10^ and may reflect early network fragility or emerging impairment. Patients with PD-nH, who are less likely to experience cognitive decline^12^, maintain a more stable, cognitively healthy brain fingerprint anchored in these core systems. Relative to those with PD-nH, patients with PD-MH also exhibited lower ΔICC in the subcortical regions and their connection with DMN and FPN. Although speculative, these fingerprinting results are compatible with observations from both resting-state and task-based studies and the attentional network hypothesis. This hypothesis posits that hallucinations are associated with overactivation of the DMN and SAL networks, whose interaction may lead to failures in stimulus-driven control and a bias towards internally generated perceptions^27^. Such overactivation could be reflected in reduced reliability of these networks.

By contrast, patients with PD-MH exhibited a stronger ΔICC in FPN within-network connections as well as FPN–DAN and FPN–CRB connections. Qualitatively, the FPN ICC values were also higher for patients with PD-MH compared with HCs (Supplementary Fig. 1). Although it is difficult to reconcile fingerprint results with findings from activation studies, our results are compatible with evidence of increased frontal theta activity in patients with MHs, associated with lower cognitive functioning in an earlier EEG study^9^ and potentially reflecting early compensatory mechanisms.

We also observed increased ΔICC in within-cerebellar pairs as well as in its connectivity with the DAN, SMT and VIS networks. Qualitatively, cerebellar ICC was also higher in the PD-MH group than in HCs (Supplementary Fig. 1). Cerebellar lesions have been associated with hallucinations in a cohort of >80 patients^53^, and reduction in gray-matter volume in the CRB has been implicated in MHs in a previous study^54^. As argued in a recent study^55^, there are a substantial number of findings providing support for the role of the CRB in psychosis as well as its role in the construction and adaption of internal models integrating sensorimotor inputs and cognitive control. This is in line with previous accounts for MHs highlighting the importance of the somatosensory and motor cues, and the integration of these signals in MHs, including presence hallucinations, which have recently been linked to specific somatomotor processes in patients with PD^31^ and in healthy individuals in whom presence hallucinations have been induced experimentally using somatomotor conflicts (based on tactile, motor and proprioceptive signals)^56^, associated with activation of primary motor and premotor cortex (compare with Fig. 2e in ref. ^31^). We note that only 0.5% of the top ΔICC PD-MH versus PD-nH edges overlapped with those differentiating PD from HC, confirming that these effects primarily reflect MH-specific features (Supplementary Fig. 1).

These differential results across the two patient groups were further supported by the finding that the identification of patient-specific functional networks was associated with different clinical traits—a result observed only when using ΔICC-based features selection and not when using group-level *t*-test differences. This suggests that ΔICC-based and *t*-test-based feature selections tap into complementary aspects of the functional architecture: the former identifies stable fingerprint-like connections that relate to clinical variation, whereas the latter isolates edges with maximally different mean connectivity between groups, which, however, are not associated with clinical scores. Similarly, unconstrained models using all possible connections failed to show significant association with clinical outcomes, highlighting the relevance of ΔICC-based fingerprinting-derived features in their association with clinical outcomes. In patients with PD-MH, stronger connectivity in these regions was associated with lower scores for frontal–subcortical functions (PD-CRS score), that is, with worse cognition (see Fig. 3b). Thus, although patients with early PD-MH (including the present sample) generally have normal cognitive scores, they may show subtle subclinical deficits in frontal–subcortical functions (including executive and attentional functions^9,57^). Standard resting-state fMRI analyses at the group level reported that altered connectivity in a priori defined fronto-parietal connections in PD-MH was associated with frontal–subcortical scores^31^. The present study sustantially extends these findings by showing that individualized functional connectivity patterns, rather than group-level effects, can be derived in an entirely data-driven manner and are detected in patients with PD-MH with early subclinical cognitive alterations. This concerned only frontal–subcortical functions and was not observed in patients with PD-nH. Together, these results suggest that MHs in PD involve a dysfunction of frontal-executive and cerebellar connectivity, differentiating patients with and without MHs. In contrast, for patients with PD-nH who typically show no or less rapid cognitive decline, we found that greater disconnection in regions with high fingerprint was associated instead with worse motor functions (UPDRS III total score, see Fig. 3b), further distinguishing a motor-only form of PD from a motor-cognitive form of PD. This may allow early distinction between both groups and different therapeutical strategies, as recent work has revealed different clinical trajectories and different sensitivities to disease-modifying treatments^3^.

Given these findings, it is important to clarify how our feature-selection approach captures individualized individual-specific information while leveraging symptom-level distinctions. The ICC-derived features are defined at the group level. Although the set of ICC-selected connections is identical for all individuals within a clinical group, each participant expresses these features through their own connectivity strengths, which remain fully individual-specific. Group-level ICC serves to identify connections that show reliable test–retest FC patterns in patients in the PD-MH and PD-nH groups, and ΔICC isolates those with the largest stability differences between groups. This approach allowed us to focus on MH-related connections while still leveraging the individual variability in connectivity strength for personalized prediction. In other words, the present study shows a method to derive individualized alterations among individuals sharing the same symptom/clinical features.

We argue that these findings are particularly noteworthy as they could open new avenues for individualized neuromodulation targets. Although the symptom-specific approach—where stimulation is personalized based on the most burdensome symptoms using group-derived targets—has notably advanced the field^58^, our results suggest a complementary individualized network-based strategy. Specifically, functional connections with high symptom-related fingerprint could inform which brain networks to target in a given patient. In practice, this could involve identifying the regions or subnetworks showing the strongest individual fingerprint alterations and using these as candidate sites for neuromodulation (for example, with real-time neurofeedback, focused ultrasound, transcranial magnetic stimulation or deep brain stimulation). If replicated in other cohorts, this approach could pave the way for algorithms that translate individualized network fingerprints into patient-specific stimulation protocols, moving beyond symptom-based personalization.

Finally, our analyses revealed a clear and distinctive neurochemical signature of MH-related brain fingerprints. Functional connections with stronger MH-related brain fingerprints (that is, positive ΔICC) were located in regions with lower cortical density of serotonin (5-HT2A), GABA and acetylcholine muscarinic M1 receptors, whereas connections with stronger fingerprint in PD-nH (that is, negative ΔICC) were located in regions with higher densities of these receptors. No significant associations were observed with dopaminergic receptors (D1, D2 and DAT), consistent with the fact that ΔICC primarily captures differences between PD subtypes, both of which share dopaminergic alterations. These findings also align with previous work on complex visual hallucinations^59^, emphasizing the role of non-dopaminergic neurotransmitters in PD-related hallucinations. Post-mortem studies have reported increased 5-HT2A receptor binding in the inferolateral temporal cortex of patients with PD and hallucinations^60^, and in vivo studies have shown higher binding in the visual, dorsolateral and medial–orbitofrontal cortices, and insula in patients with PD with hallucinations^49^. Similarly, occipital GABAa reduction^50^ and muscarinic receptor blockade^61^ along with gray-matter loss in cholinergic regions^62^ have been linked to hallucinations. Notably, these regions do not overlap with the MH-related brain-fingerprint areas. A related MEG study compared patients with PD to HCs and similarly reported negative co-localization between fingerprinting metrics and serotonin receptors (although no such effects were observed for GABA or M1)^46^, raising questions about the MH-relatedness of our findings. Direct comparison is, however, challenging as MEG and fMRI capture different physiological processes and hallucinations were not characterized in that study, leaving the possibility that some patients experienced MHs, which occur in up to 40% of patients with PD^9,30^. Nevertheless, our observations raise the intriguing possibility that the within-session stability of connectivity captured by brain-fingerprint ICC may reflect compensatory mechanisms in regions relatively spared from hallucination-related pathology, pointing to a new avenue for mechanistic understanding of hallucinations in PD.

The present study has some limitations. First, although the sample size is relatively small, this study offers a rare opportunity to investigate a cohort stratified by MHs. Such databases are scarce, making this work a valuable step towards understanding the brain fingerprint of this specific group of patients and early in the disease course. Future studies including larger and/or more densely sampled cohorts are warranted to further validate these findings. In addition, extending this framework to other patient populations presenting with MHs would be of interest (for example, ref. ^29^). Second, we agree that hallucinations are difficult to quantify, unlike other clinical signs such as tremor. Our group has addressed this by studying hallucinations in PD using behavioral paradigms, including experiments inducing presence hallucinations with robotic and virtual reality devices^31,63,64^, and comparing these induced experiences with the real-life hallucinatory symptoms of patients. Future neuroimaging studies should integrate such behavioral hallucination data and account for individual patient sensitivity. Third, this study divided the same scanning session into two to estimate brain identifiability, addressing challenges in obtaining two separate test–retest sessions in clinical cohorts as in a previous study^45^. Although this approach has been shown to yield similar results to between-session data^37^, future research to replicate our findings is needed. Fourth, although approximately 200 s of resting-state data are sufficient for good identifiability, longer acquisitions—as used in precision fMRI^65^—typically yield more robust connectivity estimates; future studies should therefore examine how fingerprint features change with extended scan durations. Finally, although our findings provide valuable insights into the brain-fingerprint features of patients with PD and MHs, it would be particularly beneficial to examine the longitudinal evolution of these features and their relationship with the variability of hallucinatory phenomena. Furthermore, future studies should investigate whether these fingerprinting patterns can predict subsequent cognitive impairment or adapt in response to emergent clinical symptoms.

In conclusion, our study provides novel evidence that fMRI brain fingerprints are preserved in PD and differ in patients with and without MHs. These differential fingerprinting features linked to distinct neurobiological substrates and allowed the identification of patient-specific functional networks associated with clinical traits. Notably, we identified patient-specific functional networks associated with subclinical cognitive impairment in patients at the early stage of PD with MHs, a subtype associated with cognitive decline and dementia. This fully data-driven framework could therefore open avenues for personalized early intervention, prognostic and therapeutic strategies. Our findings also align with previous research linking MHs to somatosensory and motor integration and frontal networks, reinforcing the relevance of these cortical networks in patients with PD and MHs^9,31^. Future work should explore how these patterns evolve alongside disease progression and whether they can be leveraged as early biomarkers for cognitive decline susceptibility in PD.

## Methods

This study utilized the clinical and PD imaging data originally presented in ref. ^35^.

### Participants

The study included *n* = 32 patients with PD from a previously study^35^ and *n* = 31 HCs (collected at Newcastle University) matched for age, sex and years of education. All patients fulfilled the MDS new criteria for PD^66^ with MHs (PD-MH; *n* = 18) and without hallucinations (PD-nH; *n* = 14). Patients in the PD-MH group included those experiencing presence (*n* = 11) and passage (*n* = 10) hallucinations, visual illusions or pareidolias (*n* = 6) and/or a combination of two or more types of MHs (*n* = 9). A summary of the demographics of the three groups is provided in Table 1. The presence and type of MHs were assessed by clinical professionals using the Hallucinations and Psychosis item of the MDS-UPDRS part I (ref. ^67^) and a semistructured interview capturing the range of MHs described in the literature (Supplementary Material in ref. ^35^). Participants reporting episodes of sense of presence, passage hallucinations, visual illusions or pareidolias at least weekly over the last month were classified as individuals with MHs. Exclusion criteria included a history of major psychiatric illness, cerebrovascular events or any medical condition other than PD that could affect cognitive functions. Individuals showing focal structural abnormalities on MRI or presenting with uncontrolled systemic disorders (such as poorly managed diabetes or hypertension) were not eligible. For patients experiencing motor fluctuations, cognitive testing was carried out during their ‘on’ phase. All participants had been maintained on stable dopaminergic treatment for at least 4 weeks before enrollment and only patients whose hallucination profile had remained unchanged for the preceding 3 months were included. No patients had a history of antipsychotic medication use. Vision was normal or corrected to normal in all participants.

### Image acquisition parameters

For the patients with PD, MRI scans were acquired using a 3T Philips Achieva system at the Hospital de la Santa Creu i Sant Pau in Barcelona. T1 weighted scans were obtained using an MPRAGE sequence (repetition time (TR) = 500 ms, echo time (TE) = 50 ms, flip angle = 8°, field of view = 23 cm with in-plane resolution of 256 × 256, slice thickness = 1 mm). Resting-state fMRI images were collected using an echo-planar sequence (TR = 2,000 ms, TE = 30 ms, flip angle = 78°, field of view = 240 mm, slice thickness = 3 mm, 180 volumes). For the HCs, MRI scans were acquired at Newcastle University using a 3T Philips Intera Achieva system (software release R5.1.7). T1 weighted scans were obtained using an MPRAGE sequence (TR = 8.3 ms, TE = 4.6 ms, flip angle = 8°, in-plane resolution = 1.0 × 1.0 mm^2^, slice thickness = 1.0 mm). Resting-state fMRI images were collected using a gradient echo echo-planar imaging sequence with 33 contiguous axial slices (TR = 2,072 ms, TE = 30 ms, field of view = 192 × 192 mm^2^, in-plane resolution = 3.0 × 3.0 mm, slice thickness = 3 mm with 1.0 mm gap, 290 volumes). Parallel imaging was performed using SENSE with an acceleration factor of two. To mitigate the confounding effect of unequal time-series length on fingerprinting metrics and to match the temporal resolution of the patient cohort, we limited the analyses to the first 180 of the 290 acquired volumes. This corresponded to 6 and 6.2 min of data for patients with PD and HCs, respectively.

### Image processing

An in-house MATLAB code based on state-of-the-art fMRI processing guidelines^68–70^, using the open-source MATLAB toolbox Apéro (https://github.com/juancarlosfarah/apero), was used to preprocess fMRI data. Preprocessing included standard structural and functional steps (denoising, segmentation, motion correction, nuisance regression, temporal filtering and parcellation). Full methodological details are provided in Supplementary Information. A whole-brain data-driven functional parcellation based on 278 regions including cortical and subcortical areas, as obtained by Shen et al.^71^, was projected into the T1 space of each study participant (FSL FLIRT six degrees of freedom, FSL FLIRT 12 degrees of freedom and finally FSL FNIRT) and then into the native EPI space of each study participant^72^.

### Functional connectivity and whole-brain within-session brain fingerprint

We estimated individual FC matrices using the Pearson’s correlation coefficient between the averaged signals of all region pairs. The resulting individual FC matrices were composed of 278 cortical nodes, as obtained in previous work ^71^. Finally, the resulting FCs were ordered according to seven cortical RSNs as proposed in ref. ^73^, plus subcortical and cerebellar regions (similarly to ref. ^74^; Fig. 1a).

We estimated within-session identifiability or fingerprinting, following the approach proposed. This method involves splitting the fMRI times series in halves and enables quantification of within-session connectome fingerprints. Previous work has demonstrated that this method produces very similar results to those obtained from data acquired across separate sessions (that is, between-sessions fingerprint) in healthy study participants from the Human Connectome Project (Supplementary Fig. 3 in ref. ^75^). Although within- and between-sessions fingerprinting held similar results, they are different approaches to quantifying the brain fingerprint and this should be compared in future studies. However, we note that there are no clinical datasets of patients with PD-MH available that include two fMRI sessions acquired within a short-time gap (that is, within around 1 or 2 weeks). Therefore, within-session fingerprint is at present the only method available for estimating brain fingerprint in patients with PD-MH. In this study, we estimated identifiability across the first-half (test) and second-half volumes (retest) within the same scan. Recent work has shown that a good level of identifiability across the different resting-state networks can be reached from around 200 s (Fig. 4b in ref. ^76^). In this work, each test and retest session had 90 volumes with a TR of 2 s, therefore providing sufficient data for achieving a good success rate and identifiability across the entire brain.

At the whole-brain level, the fingerprint was calculated for each study participant (*s*) as test–retest similarity between FCs (Fig. 1b; we called this metric ISelf).

$${\rm{ISelf}}(s)={\rm{corr}}({{\rm{FC}}}_{{\rm{test}}}(s),{{\rm{FC}}}_{{\rm{retest}}}(s))$$

Then, for each *s* we computed an index of the FCs similarity with the other study participants (*i*) in their group (IOthers), where N is the total number of individuals in each group:

$$\mathrm{IOther}(s)=\frac{{\sum }_{i\ne s}\displaystyle (\mathrm{corr}({\mathrm{FC}}_{\mathrm{test}}(s),{\mathrm{FC}}_{\mathrm{retest}}(i))+\mathrm{corr}({\mathrm{FC}}_{\mathrm{retest}}(s),{\mathrm{FC}}_{\mathrm{test}}(i)))}{2N-2}$$

A second metric, IDiff (Fig. 1b), provides a group-level estimate of the within- (ISelf) and between-individual (IOthers) test–retest reliability distance, where ${\rm{Subj}}$ is the set of study participants:

$${\rm{IDiff}}={\rm{mean}}\,{\rm{ISelf}}(s)-{\rm{mean}}\,{\rm{IOthers}}(s)$$

$$s\in {\rm{Subj}}\,s\in {\rm{Subj}}$$

Finally, we measured the Success-rate^36^ of the identification procedure as the percentage of cases with higher ISelf versus IOthers test–retest reliability. These metrics have been introduced and estimated in patients^77^ and healthy populations^75^ in previous work.

### Spatial specificity of brain fingerprint using edgewise ICC

Spatial specificity of FC fingerprints was derived using edgewise ICC, computed separately for each functional connection (edge) and for each group. For any given edge, the ICC was calculated across individuals using test and retest FC values as repeated measurements according to a one-way random-effects model (ICC(1,1))^78^ (Fig. 2a):

$${\rm{ICC}}=\frac{{{\rm{MS}}}_{{\rm{R}}}-{\rm{M}}{{\rm{S}}}_{{\rm{W}}}}{{{\rm{MS}}}_{{\rm{R}}}+(k-1){{\rm{MS}}}_{{\rm{W}}}}$$

where ${\rm{M}}{{\rm{S}}}_{{\rm{R}}}\,$ is the mean square between individuals (rows), ${\rm{M}}{{\rm{S}}}_{{\rm{W}}}$ the mean square for residual sources of variance (within-individual variability across sessions) and k is the number of sessions (here, test and retest). A high ICC for a given edge indicates that a larger proportion of the variance across test and retest is due to differences between the study participants, rather than within-individual differences between test and retest or random noise. Conversely, a low ICC indicates greater within-subjects variability than across subjects. Thus, edges with high ICC values represent connections that are both reliable within individuals and discriminative across participants, that is, driving identifiability consistent with the concept of functional connectivity fingerprint^75,76^. We note that ICC does however not directly infer differences between individuals.

The ICC has been commonly used in the functional connectivity literature to assess reliability^75,79–82^. A few strengths of the ICC include: the ability to assess absolute agreement in repeated measurements of an object (unlike, for example, Pearson’s correlation, wherein variables are scaled and cantered separately), the ability to explicitly model multiple known sources of variability (for example, scanner, brain response and head motion) and comparing within and between variability across the objects of measurement. Depending on whether and how sources of error (or ‘facets’; for example, scanner) may be specified, one of three ICC forms may be used^83^. Briefly, usage is as follows: ICC(1,1) is used to estimate agreement in exact values when sources of error are unspecified, ICC(2,1) is often referred to as ‘absolute agreement’ and is used to estimate agreement in exact values when sources of error are known (for example, repeated runs) and modeled as random, and ICC(3,1) is often referred to as or ‘consistent agreement’ and is used to estimate agreement in rankings when sources of error are known and modeled as fixed (resulting in a mixed effects ANOVA). In this Article we used ICC(1,1), following previous works^75^, because variability in fMRI data can come from different known (for example, scanner and head motion) and unknown sources. In the statistics literature the ICC is akin to a measure of discriminability and is commonly categorized as follows: poor, <0.4; fair, 0.4–0.59; good, 0.6–0.74; and excellent, ≥0.75 (ref. ^84^). In this Article, this categorization was taken as reference for the thresholds on the ICC matrices, which was set at 0.6 (good)^84^.

To control for sample-size differences across groups, bootstrapping was used: for each group, ICC was calculated across test and retest for subsets of randomly chosen *n* = 10 participants, across 1,000 bootstrap runs and then averaged within each group (Fig. 2a). Bootstrapping was performed in MATLAB using an in-house function.

### Minor hallucination-related differential ICC

In these analyses we aimed to identify edges that were MH-related, that is, those that contributed to differences in fingerprint between patients with PD-MH and PD-nH. To achieve this, we first computed a ΔICC matrix to capture MH-related differential identifiability defined as:

$$\Delta {{\rm{ICC}}}_{{\rm{PD}}-{\rm{MH}}-{\rm{PD}}-{\rm{nH}}}={{\rm{ICC}}{\rm{mat}}}_{{\rm{PD}}-{\rm{MH}}}-{{\rm{ICC}}{\rm{mat}}}_{{\rm{PD}}-{\rm{nH}}}$$

As a result, the ΔICC_PD−MH – PD−nH_ matrix (mat; Fig. 2b) contained positive values for functional connections with higher ICC in PD-MH and negative values for those with higher ICC in PD-nH. Values close to zero indicated similar ICC across both groups, suggesting individual variability that did not contribute to distinguishing features between them. We identified group-specific ICC edges by selecting the positive or negative ΔICC edges for PD-MH and PD-nH, respectively. To determine whether the features most strongly distinguishing PD-MH from PD-nH were MH-related rather than more generally associated with PD, we performed the following analysis. First, we computed a second, PD-specific, differential ICC map defined as:

$$\Delta {{\rm{ICC}}}_{{\rm{PD}}-{\rm{HC}}}\,=\,\frac{{{\rm{ICC}}{\rm{mat}}}_{{\rm{PD}}-{\rm{MH}}}\,+\,{{\rm{ICC}}{\rm{mat}}}_{{\rm{PD}}-{\rm{nH}}}}{2}-{{\rm{ICC}}{\rm{mat}}}_{{\rm{HC}}}$$

Next, from this ΔICC_PD–HC_ map, we retained only edges whose absolute values exceeded the minimum absolute value within the top 10% of ΔICC_PD-MH − PD-nH_ (shown in Supplementary Fig. 1b) and whose sign matched the direction of ΔICC_PD-MH – PD-nH_. This was done to test the extent to which PD–HC ICC differences followed the same directionality of the strongest PD-MH versus PD-nH ICC differences, thereby assessing whether edges most strongly associated with MHs overlapped with those reflecting PD-specific alterations. Finally, we quantified the overlap (Supplementary Fig. 1c).

We then computed nodal strength of these MH-related ICC matrices to visualize ICC hubs on the cortical surface (using BrainNet^85^). To highlight most prominent hubs, we applied a threshold, displaying only brain regions with nodal strength above the 75th percentile (Fig. 2c). Finally, we examined the distribution of the MH-related ICC edges across functional networks. Specifically, we computed the percentage of edges per network exceeding a ΔICC of ±0.5 (corresponding to the 97th percentile and considered to reflect a fair ICC^84^) separately for PD-MH and PD-nH, and tested for group differences using a binomial test (function binocdf in MATLAB), applying FDR correction for multiple comparisons (Fig. 2d). Results for analyses without thresholding, as well as for higher thresholds (98th and 99th percentiles), are reported in Supplementary Information.

### Associating clinical scores with brain-fingerprint features

In these analyses we tested whether clinical scores were associated with individuals’ functional connections that most strongly distinguished the two groups in terms of fingerprint. To identify these key connections, we selected in the top-0.1th percentile edges for each group-specific ICC matrix. These edges were visualized on the cortical surface using BrainNet^85^ (Fig. 3a). The binarized matrices served as masks to extract high-fingerprint edges from the FC of each individual (Fig. 3c). For each individual, we separated positive and negative functional connections to prevent mathematical cancellation of connectivity values. We then computed two summed connectivity scores: one for positive and one for negative connections. Next, we fitted linear regression models (using the fitlm function in MATLAB) to examine the relationship between clinical scores based and individual functional connectivity (Fig. 3b). Each model included a clinical score as the dependent variable and a functional connectivity value for each individual as the predictor, while controlling for age, years of education and disease duration. In addition to ICC-based selection, we repeated the regression analyses using edges showing significant between-group differences identified via two-sample *t*-tests (by selecting the top 0.1% of edges with the highest *t* values for each group) as well as using the full FC without any feature selection. The same modeling approach and covariates were applied in all analyses. These complementary analyses allowed us to test whether the brain fingerprinting approach is advantageous over different edge selection approaches, including data-driven group differences and the full connectome.

The clinical scores analysed included the UPDRS III total, a global measure of motor-symptom severity, where higher scores reflect greater impairment^67^. We also examined the PD-CRS frontal–subcortical score, a composite measure of executive function that includes sustained attention, working memory, verbal fluency, clock drawing, and verbal memory tasks, where lower scores reflect greater impairment. This score is sensitive to mild cognitive impairment in PD^86^ and has been linked to neural alterations specific to patients with PD and MH^9,31^. In contrast, the PD-CRS Posterior score, which assesses confrontation naming and clock copying, is reportedly altered later, in the transition to dementia^86^.

### Association between cortical topography of brain fingerprint and neurotransmitter systems

In these analyses we looked at which neurotransmitter systems showed topographical correspondence with the brain-fingerprint topography (Fig. 4). We estimated the spatial correlation between the ΔICC map derived from nodal strength of the ΔICC matrix and each of the normative atlas maps of the neurotransmitters’ receptors of dopamine, serotonin, GABA and acetylcholine given their implication in the neurobiology of hallucinations. Normative neurotransmitter density data are available from neuromaps (https://github.com/netneurolab/neuromaps)^87^.

For serotonin, 5-HT1A and 5-HT2A receptor densities were derived from positron emission tomography (PET) tracer binding potential (BPnd) in 95 healthy individuals^88^. For dopamine, D1 and D2 densities were derived from PET BPnd in 13 (ref. ^89^) and seven^90^ healthy individuals, respectively, with D2 measured across two retest sessions. Dopamine transporter (DAT) density was based on the SPECT standardized uptake value ratio in healthy volunteers^91^. GABAa density was derived from PET BPnd^91^. For acetylcholine, muscarinic M1 receptor density was obtained from PET BPnd in six HCs across test–retest sessions^92^, and nicotinic α4β2 receptor density was derived from PET free-fraction corrected distribution volume (VT) in eight healthy individuals^93^.

Each receptor map was parcellated using the 278 parcellation from ref. ^71^ to match the ΔICC map. Spearman rank correlations were computed between each receptor map and the ΔICC map. To assess statistical significance while accounting for spatial autocorrelation, null distributions were generated by computing Spearman correlations between the ΔICC map and 1,000 spatially permuted surrogate receptor maps using Moran spectral randomization (using neuromaps.nulls.moran from neuromaps^87^). Two-tailed *P* values were computed as the proportion of permuted correlations equal to or more extreme than the observed correlation. To correct for multiple comparisons, we applied FDR correction using the Benjamini–Hochberg procedure (multipletests function from the Python statsmodels package).

### Statistics and reproducibility

The statistical tests reported in this paper are two-sided and performed in RStudio 2022.07.2 (ref. ^94^), Python 3.9.18 and MATLAB R2022a^95^. Normality assumptions were checked before performing the analyses using the Shapiro–Wilk normality test (Shapiro_test R function, rstatix package or function swtest in MATLAB). When normality was not met, nonparametric equivalent was used, as detailed below. No statistical method was used to pre-determine the sample size of the patient group. Healthy controls were chosen from a pool to match the patient group in sample size, age, sex and education. No data were excluded from the analyses.

#### Demographics

Differences across groups were tested using a Fisher’s test for sex, and an ANOVA (for PD-MH, PD-nH and HC comparisons), two-samples *t*-test (for PD-MH and PD-nH comparisons) or their nonparametric equivalent—that is, Wilcoxon signed-rank and Kruskal–Wallis test—for the remaining variables. There were no differences across groups (that is, PD-MH versus PD-nH versus HCs) for sex (*P* = 0.652), age (*P* = 0.094) and years of education (*P* = 0.445). Patients did not differ for motor symptoms severity (UPDRS III total, *P* = 0.238), cognitive functions (PD-CRS frontal–subcortical, *P* = 0.848; PD-CRS posterior, *P* = 0.875), disease duration (*P* = 0.253) or daily dose of L-dopa (*P* = 0.394) and dopamine agonist dose (*P* = 0.833; Table 1).

#### ISelf and IOthers

ISelf and IOthers were normally distributed in each group. Analyses were performed using MATLAB and RStudio. To compare ISelf versus IOthers in each group we performed paired-sample *t*-test (ttest, MATLAB 2022a). We then used a one-way ANOVA to test the effect of group on ISelf and IOthers separately after checking for nuisance variables, with 5,000 permutations to control for sample-size differences (using aovperm from the permuco R-package^96^; Supplementary Table 1). For ISelf, the nuisance variables were age, sex, years of education (YoE), site of acquisition and the difference in motion between the test and retest scans, as absolute difference between FD (ISelf ~ group + age + sex + YoE + site + ΔFD). For IOthers, the nuisance variables were also age, sex, YoE and site of acquisition but motion (FD) was considered across the entire acquisition as IOthers is a composite measure across test and retest (IOthers ~ group + age + sex + YoE + FD). Finally, we performed permutation testing analysis to compare Success-rate and IDiff from 1,000 surrogate datasets of random ID matrices against the real value^97^. Permutation analyses were implemented in MATLAB using an in-house function where a permuted version of the ID matrix was built by randomly shuffling its elements.

### Inclusion and ethics statement

All participants gave their informed consent for inclusion before they participated in the study, in accordance with the guidelines of the Declaration of Helsinki. All ethical regulations relevant to human research participants were followed. Patients were recruited at the Movement Disorders Clinic at Hospital de la Santa Creu i Sant Pau (Barcelona) and the study was approved by the local Ethics Committee (study code IIBSP-PAR-2014-59; version: 14 April 2014). Healthy controls were recruited at Newcastle University. Ethical approval was obtained from the National Research Ethics Service Committee North East–Newcastle and North Tyneside 2 (REC reference numbers: 12/NE/0290 and 13/NE/0252).

### Reporting summary

Further information on research design is available in the Nature Portfolio Reporting Summary linked to this article.

## Supplementary information

Supplementary Information Supplementary Table 1, Figs. 1 and 2, Methods (image processing) and Analyses (whole-brain fingerprinting, MH-related differential identifiability and network differences in hallucination-related brain fingerprinting in PD).

Reporting Summary

Peer Review File

## Data Availability

The raw neuroimaging data supporting the findings of this study cannot be made publicly available in a repository, as the data contain identifiable features that could compromise the anonymity of study participants. In accordance with ethical obligations and the informed consent provided by participants, raw data may be made available on reasonable request to the corresponding authors, subject to approval by the local ethics committee. The derived FC matrices and behavioral data necessary to reproduce the main analyses of this study are available in S.S.’s GitHub repository (https://github.com/ss1913/fingerprints_parkinson_mh).

## Code availability

The full code necessary to reproduce the main results and figures are available in S.S.’s GitHub repository (https://github.com/ss1913/fingerprints_parkinson_mh).
