# Supplementary material for: Patient-specific functional networks track early cognitive alterations and minor hallucinations in Parkinson’s disease
Source: Nat Ment Health. 2026 Jun 8;4(6):1021–34. doi: 10.1038/s44220-026-00657-x (PMC13652722; doi:10.1038/s44220-026-00657-x)
Supplement: Supplementary file 1 [file 44220_2026_657_MOESM1_ESM.pdf]

nature mental health

Supplementary information https://doi.org/10.1038/s44220-026-00657-x

Patient-specific functional networks track early cognitive alterations and minor hallucinations in Parkinson's disease

In the format provided by the authors and unedited

Supplementary Materials

Supplementary methods: image processing

fMRI data were preprocessed using in-house MATLAB code based on state-of-the-art fMRI processing guidelines [70–72], using the open-source MATLAB toobox Apéro (https://github.com/juancarlosfarah/apero). Structural images were first denoised to improve the signal-to-noise ratio [73], bias-field corrected, and then segmented (FSL FAST) to extract white matter, grey matter and cerebrospinal fluid (CSF) tissue masks. These masks were warped in each individual subject's functional space by means of subsequent linear and non-linear registrations (FSL flirt 6dof, FSL flirt 12dof and FSL fnirt). The following steps were then applied on the fMRI data: BOLD volume unwarping with applytopup, slice timing correction (slicetimer), realignment (mcflirt), normalisation to mode 1000, demeaning and linear detrending (MATLAB detrend), regression (MATLAB regress) of 18 signals: 3 translations, 3 rotations, and 3 tissue-based regressors (mean signal of whole-brain, white matter (WM) and cerebrospinal fluid (CSF), as well as 9 corresponding derivatives (backwards difference; MATLAB). We tagged (but did not remove) high head-motion volumes on the basis of three metrics: frame displacement (FD, in mm), standardised DVARS [74] (D referring to temporal derivative of BOLD time courses, VARS referring to root mean square variance over voxels) as proposed in [71], and SD (standard deviation of the BOLD signal within brain voxels at every time-point). The FD, DVARS were obtained with fsl_motion_outliers and SD vectors with MATLAB. Volumes were motion-tagged when FD > 0.3 mm and standardised DVARS > 1.7 and SD > 75th percentile +1.5 of the interquartile range, as per FSL recommendation [75]. All subjects had ≤30% motion-tagged volumes. There was no significant difference across groups in the percentage of tagged volumes [p=.408] or DVARS [p=.520], but there was a significant difference in the average FD [p=.048], reflecting higher framewise displacement in HC relative to PD-nH [p=.042]. There was no difference across test and retest in the percentage of tagged volumes [p=.110] nor in DVARS [0.435] but there was a difference in the average FD [p=.047], revealing that on average subjects moved more in the second-half. As mentioned above, motion effects were already regressed out from the BOLD signal. Nonetheless, FD was additionally included as a covariate in analyses of fingerprinting metrics to further control for residual movement differences across groups.

A bandpass first-order Butterworth filter [0.01 Hz, 0.15 Hz] was applied to all BOLD time-series at the voxel level (MATLAB butter and filtfilt). The choice of the bandpass filter was aligned with previous works [76] where the choice of the filtering proved to be meaningful to capture the effect of brain fingerprints in the temporal domain, and in the analogy between MEG and fMRI fingerprints [77], and with respect to the relationship between brain fingerprints and structure-function coupling [78].

The first three principal components of the BOLD signal in the WM and CSF tissue were regressed out of the grey matter (GM) signal (MATLAB, pca and regress) at the voxel level. A whole-brain data-driven functional parcellation based on 278 regions including cortical and subcortical areas as obtained by [79], was projected into each subject's T1 space (FSL flirt 6dof, FSL flirt 12dof and finally FSL fnirt) and then into the native EPI space of each subject. We also applied FSL boundary-based-registration [80] to improve the registration of the structural masks and the parcellation to the functional volumes.

Supplementary analyses: whole-brain fingerprinting

Full statistics ANOVA, 5000 permutations (IOthers and ISelf)

Supplementary Table 1. Differences across groups in *ISelf* and *IOthers*

ISelf *~ Group + Age + Sex + YoE + Site + ΔFD*

Anova Table	SS	df	F	parametric P(>F)	resampled P(>F)	p resampled signif.
Group	0.002	2	0.1	0.877	0.603	ns
Age	0.012	1	1.6	0.209	0.212	ns
Sex	0.010	1	1.4	0.243	0.244	ns
YoE	0.001	1	0.1	0.736	0.730	ns
Site	0.000	1	0.0	0.966	0.647	ns
ΔFD	0.002	1	0.2	0.632	0.632	ns

IOthers *~ Group + Age + Sex + YoE + Site +FD*

Anova Table	SS	df	F	parametric P(>F)	resampled P(>F)	p resampled signif
Group	0.001	2.0	0.7	0.502	0.257	ns
Age	0.006	1.0	6.3	0.015	0.015	*
Sex	0.000	1.0	0.0	0.861	0.856	ns
YoE	0.001	1.0	0.7	0.419	0.419	ns
Site	0.000	1.0	0.1	0.810	0.614	ns
FD	0.004	1.0	4.3	0.042	0.041	*

Legend: YoE=years of education; ΔFD=differential frame-wise displacement at test vs. retest; FD=average frame-wise displacement. p-value was estimated using ANOVA with 5000 permutations to control for sample size differences, after controlling for nuisances variables (see Table title for specs about the model). Resampling test using Freedman-Lane to handle nuisance variables and 5000 permutations; ns=not significant, $p>0.05$; *$p\leq0.05$; ** $p\leq0.01$;*** $p\leq0.001$;**** $p\leq0.0001$.

Covariate effects on between-subject similarity (IOthers)

To investigate the influence of potential confounding variables, we ran a linear model with *IOthers* as the dependent variable and Group, Age, Sex, YoE, Site, and FD as predictors. The model revealed that PD-MH showed slightly higher *IOthers* compared to HC (Estimate = 0.02; p = 0.033),

while PD-nH was not significantly different from HC. Age was negatively associated with IOthers (Estimate = -0.002, p = 0.015), indicating that older participants tended to have slightly lower similarity with others. FD was positively associated with *IOthers* (Estimate = 0.093, p = 0.042), suggesting that motion has a small influence on similarity estimates. Sex, YoE, and Site were not significant predictors.

Analyses restricted to PD subgroups (PD-MH and PD-nH) confirmed that no significant group differences in IOthers were observed (F(1,26) = 0.95, p = 0.337), and none of the covariates (Age, Sex, YoE, FD) showed significant effects. This suggests that the covariate associations observed in the full model (notably for Age and FD) are mainly driven by the inclusion of HC participants, who were scanned at a different site, rather than by variability within the PD cohort.

Supplementary analyses: minor hallucination related differential identifiability

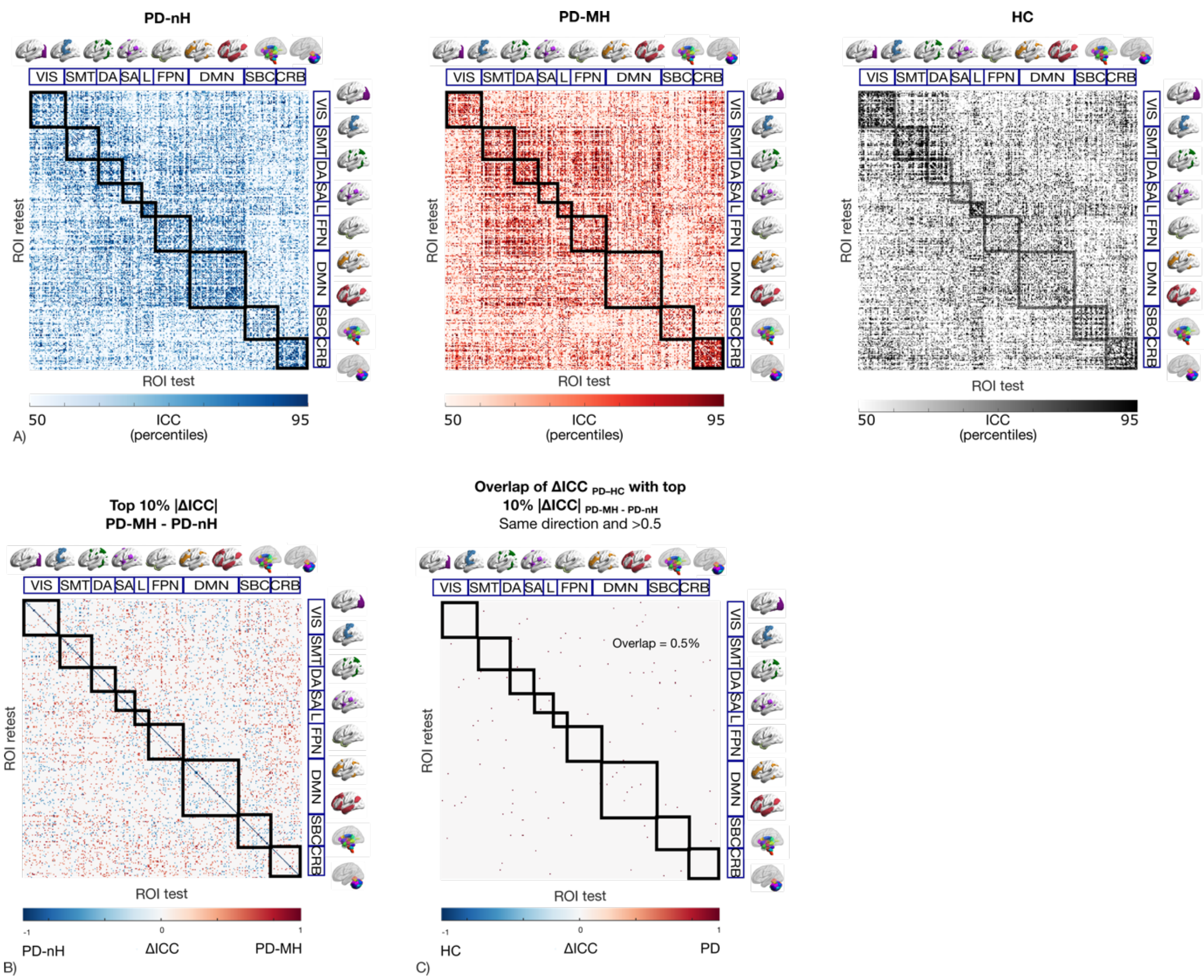

Supplementary Figure 1. Overlap between brain fingerprint features related to hallucinations and those differentiating Parkinson's disease patients from healthy controls. A) Edgewise intra-class correlations (ICC) matrices for Parkinson's Disease patients without hallucinations (PD-nH; N=14) and with minor hallucinations (PD-MH;N=18) and healthy controls (HC;N=31). ICC quantifies within-subjects similarity between test and retest for each edge (FC between 2 regions). ICC matrices for PD-nH and PD-MH are the same as those displayed in Figure 2A of the main text; they are reported here to allow visual comparison. We display edges with ICC between the 50th and the 95th percentile, across the 7 resting-state networks (RSN), subcortical and cerebellar regions. B) ΔICC matrix showing the difference in ICC between PD-MH and PD-nH, as in Figure 2B, here thresholded for the top 10% absolute values. C) ΔICC matrix showing the difference in ICC

between PD patients (PD-MH and PD-nH combined) and HC, restricted to edges showing the same direction of effect and exceeding the minimal ΔICC value observed in the PD-MH vs PD-nH comparison (ΔICC = 0.5, where ICC is considered 'good' above 0.6[1]). This corresponded to 0.5% of the total number of top edges shown in B). A), B) and C) are ordered according the 7 RSNs, subcortical and cerebellar regions. VIS=Visual Network; SMT=Somatomotor Network; DA=Dorsal Attention Network; SA=Salience Network; L=Limbic Network; FPN=Fronto-Parietal Network; DMN=Default-Mode Network; SBC=Subcortical regions; CRB=Cerebellum.

Supplementary analyses: network differences in hallucinations-related brain fingerprint in PD

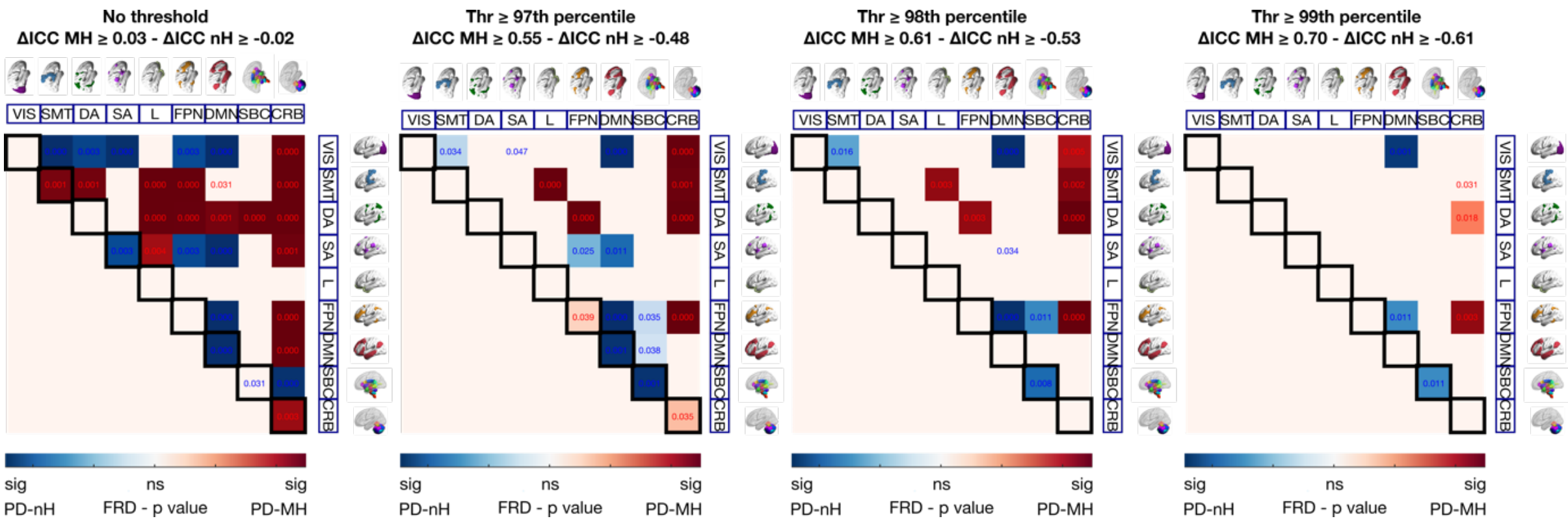

Supplementary Figure 2. Network differences in hallucination-related brain fingerprint in PD.

Matrices show network pairs with significant differences in the proportion of ΔICC edges (i.e., the hallucination-specific signature) between PD-MH (N=18) and PD-nH (N=14). This figure supplements Fig. 2D of the manuscript, demonstrating that these patterns are maintained across different thresholds. Panels are arranged from left to right: no threshold in ΔICC; threshold = 97th percentile; threshold = 98th percentile; threshold = 99th percentile. High ΔICC edges are defined as those exceeding the 97th percentile within each group (approximately corresponding to ΔICC ~ ±0.5, where ICC = 0.5 is typically interpreted as "fair", Cicchetti & Sparrow, 1981). Results are shown for all seven resting-state networks (7RSNs), subcortical, and cerebellar regions, including both within- and between-network connections. Network-pair comparisons were performed using a two-sided binomial test, testing whether the number of edges where ΔICC was higher in PD-MH exceeded what would be expected by chance (null hypothesis: p = 0.5). P-values were corrected for multiple comparisons across all network pairs using the Benjamini-Hochberg FDR procedure.. Colored cells indicate network pairs with significant group differences, with the FDR-corrected p-value reported; ns = p > 0.05. Colors reflect the directionality of the effect: blue indicates a higher proportion of high-ΔICC edges in PD-nH, and red indicates a higher proportion in PD-MH. VIS=Visual Network; SMT=Somatomotor Network; DA=Dorsal Attention Network; SA=Salience Network; L=Limbic Network; FPN=Fronto-Parietal Network; DMN=Default-Mode Network; SBC=Subcortical regions; CRB=Cerebellum.
