# Supplementary material for: Patient-specific functional networks track early cognitive alterations and minor hallucinations in Parkinson’s disease
Source: Nat Ment Health. 2026 Jun 8;4(6):1021–34. doi: 10.1038/s44220-026-00657-x (PMC13652722; doi:10.1038/s44220-026-00657-x)
Supplement: Supplementary file 2 [file 44220_2026_657_MOESM2_ESM.pdf]

nature portfolio

Corresponding author(s): Sara STAMPACCHIA

Last updated by author(s): Dec 19, 2025

Reporting Summary

Nature Portfolio wishes to improve the reproducibility of the work that we publish. This form provides structure for consistency and transparency in reporting. For further information on Nature Portfolio policies, see our Editorial Policies and the Editorial Policy Checklist.

Statistics

For all statistical analyses, confirm that the following items are present in the figure legend, table legend, main text, or Methods section.

n/a	Confirmed	
☐	☒	The exact sample size (*n*) for each experimental group/condition, given as a discrete number and unit of measurement
☐	☒	A statement on whether measurements were taken from distinct samples or whether the same sample was measured repeatedly
☐	☒	The statistical test(s) used AND whether they are one- or two-sided<br>*Only common tests should be described solely by name; describe more complex techniques in the Methods section.*
☐	☒	A description of all covariates tested
☐	☒	A description of any assumptions or corrections, such as tests of normality and adjustment for multiple comparisons
☐	☒	A full description of the statistical parameters including central tendency (e.g. means) or other basic estimates (e.g. regression coefficient) AND variation (e.g. standard deviation) or associated estimates of uncertainty (e.g. confidence intervals)
☐	☒	For null hypothesis testing, the test statistic (e.g. *F*, *t*, *r*) with confidence intervals, effect sizes, degrees of freedom and *P* value noted<br>*Give P values as exact values whenever suitable.*
☒	☐	For Bayesian analysis, information on the choice of priors and Markov chain Monte Carlo settings
☒	☐	For hierarchical and complex designs, identification of the appropriate level for tests and full reporting of outcomes
☐	☒	Estimates of effect sizes (e.g. Cohen's *d*, Pearson's *r*), indicating how they were calculated

Our web collection on statistics for biologists contains articles on many of the points above.

Software and code

Policy information about availability of computer code

Data collection	No software was used to acquire the data. The imaging data were acquired at the Hospital de la Santa Creu i Sant Pau in Barcelona for PD patients using a 3T Philips Achieva scanner, and at Newcastle University with a 3T Philips Intera Achieva scanner for healthy controls.
Data analysis	Custom-code was implemented in Matlab R2024b for fingerprinting analyses, and in R Studio 2022.07.2 for statistical analyses. Python scripts developed in Visual Studio Code were used for the neurotransmitter association analyses. The full code necessary to reproduce the main results and figures of the work is available online in a maintained GitHub repository (https://github.com/ss1913/fingerprints_parkinson_mh).

For manuscripts utilizing custom algorithms or software that are central to the research but not yet described in published literature, software must be made available to editors and reviewers. We strongly encourage code deposition in a community repository (e.g. GitHub). See the Nature Portfolio guidelines for submitting code & software for further information.

Data

Policy information about availability of data

All manuscripts must include a data availability statement. This statement should provide the following information, where applicable:

- Accession codes, unique identifiers, or web links for publicly available datasets
- A description of any restrictions on data availability
- For clinical datasets or third party data, please ensure that the statement adheres to our policy

The raw neuroimaging data supporting the findings of this study cannot be made publicly available in a repository, as the data contain identifiable features that

could compromise the anonymity of study participants. In accordance with ethical obligations and the informed consent provided by participants, raw data may be made available upon reasonable request to the corresponding author, subject to approval by the local ethics committee. PD dataset: available from co-authors Javier Pagonabarraga and Jaime Kulisevsky. HC dataset: available from John-Paul Taylor and Alan Thomas. The derived FC matrices and behavioural data necessary to reproduce the main analyses of this study is available online in SS's GitHub repository (https://github.com/ss1913/fingerprints_parkinson_mh). The full code necessary to reproduce the main results and figures is available online in SS's GitHub repository (https://github.com/ss1913/fingerprints_parkinson_mh).

Research involving human participants, their data, or biological material

Policy information about studies with human participants or human data. See also policy information about sex, gender (identity/presentation), and sexual orientation and race, ethnicity and racism.

Reporting on sex and gender	Participants' sex (as a biological attribute) was based on self-report. Sex-stratified analyses were not performed due to insufficient sample size for meaningful subgroup comparisons. The proportion of females and males did not differ significantly across groups, although males were more frequent overall, consistent with the known sex distribution of Parkinson's disease. PD with minor hallucinations: 8/18 females (44.4%); PD without hallucinations: 4/14 females (28.6%); Healthy controls: 10/31 females (32.3%).
Reporting on race, ethnicity, or other socially relevant groupings	Information about participant's race, ethnicity or other socially relevant information was not available or known.
Population characteristics	Covariate relevant population characteristics included sex, age, years of education and disease duration.
Recruitment	PD patients were prospectively recruited from a sample of outpatients regularly attending to the Movement Disorders Clinic at Hospital de la Santa Creu i Sant Pau , Barcelona. Healthy controls were recruited at Newcastle University.
Ethics oversight	Patients were recruited at the Movement Disorders Clinic at Hospital de la Santa Creu i Sant Pau (Barcelona) and the study was approved by the local Ethics Committee. Healthy controls were recruited at Newcastle University and the study was approved by the local Ethics Committee.

Note that full information on the approval of the study protocol must also be provided in the manuscript.

Field-specific reporting

Please select the one below that is the best fit for your research. If you are not sure, read the appropriate sections before making your selection.

☒ Life sciences ☐ Behavioural & social sciences ☐ Ecological, evolutionary & environmental sciences

For a reference copy of the document with all sections, see nature.com/documents/nr-reporting-summary-flat.pdf

Life sciences study design

All studies must disclose on these points even when the disclosure is negative.

Sample size	N=32 patients with PD and N=31 healthy controls (HC) were included in the analyses. All patients were included if they fulfilled the MDS new criteria for PD. HC were matched for age, sex and years of education, and collected at Newcastle University. Patients were stratified for having or not minor hallucinations in daily life (see below). This stratification of patients is uncommon, and therefore the sample size was determined by data availability rather than by an a priori power calculation.
Data exclusions	Exclusion criteria included a history of major psychiatric illness, cerebrovascular events, or any medical condition other than PD that could affect cognitive functions. Individuals showing focal structural abnormalities on MRI or presenting with uncontrolled systemic disorders (such as poorly managed diabetes or hypertension) were not eligible. For patients experiencing motor fluctuations, cognitive testing was carried out during their "on" phase. All participants had been maintained on stable dopaminergic treatment for at least four weeks prior to enrolment, and only patients whose hallucination profile had remained unchanged for the preceding three months were included. None had a history of antipsychotic medication use. Vision was normal or corrected to normal in all participants.
Replication	The entire analysis pipeline is available to facilitate attempts to reproduce the experimental findings. At the time of conducting the study, external datasets with comparable patient stratification were not incorporated into the analyses.
Randomization	Presence and type of minor hallucinations were assessed by clinical professionals using the Hallucinations and Psychosis item of the MDS-UPDRS Part I and a semi-structured interview capturing the range of minor hallucinations described in the literature. Participants reporting at least weekly over the last month episodes of sense of presence, passage hallucinations, visual illusions, or pareidolias in the past month were classified as minor hallucinators. This resulted in two patient subgroups: PD with minor hallucinations (PD-MH, N=18) and PD without hallucinations (PD-nH, N=14).
Blinding	All investigators were blind to group allocation during data analyses.

Reporting for specific materials, systems and methods

We require information from authors about some types of materials, experimental systems and methods used in many studies. Here, indicate whether each material, system or method listed is relevant to your study. If you are not sure if a list item applies to your research, read the appropriate section before selecting a response.

Materials & experimental systems

n/a	Involved in the study
☒	☐ Antibodies
☒	☐ Eukaryotic cell lines
☒	☐ Palaeontology and archaeology
☒	☐ Animals and other organisms
☐	☒ Clinical data
☒	☐ Dual use research of concern
☒	☐ Plants

Methods

n/a	Involved in the study
☒	☐ ChIP-seq
☒	☐ Flow cytometry
☐	☒ MRI-based neuroimaging

Clinical data

Policy information about clinical studies

All manuscripts should comply with the ICMJE guidelines for publication of clinical research and a completed CONSORT checklist must be included with all submissions.

Clinical trial registration	NA
Study protocol	NA
Data collection	NA
Outcomes	NA

Plants

Seed stocks	NA
Novel plant genotypes	NA
Authentication	NA

Magnetic resonance imaging

Experimental design

Design type	Resting-state fMRI
Design specifications	Resting-state fMRI data were acquired in a single run of approximately 6 minutes (TR = 2000 ms, 180 volumes) for PD patients, and 10 minutes (TR = 2072 ms, 290 volumes) for healthy controls. To ensure comparability, the healthy control data were truncated to 180 volumes to match the patient scans.
Behavioral performance measures	No behavioral performance measures were collected inside the MRI.

Acquisition

Imaging type(s)	functional, anatomical
Field strength	3T
Sequence & imaging parameters	PD patients: MRI scans were acquired with a 3T Philips Achieva at the Hospital de la Santa Creu i Sant Pau in Barcelona. T1 weighted scans were obtained using a MPRAGE sequence (TR = 500 ms, TE = 50 ms, flip angle = 8, field of view [FOV]

= 23 cm with in-plane resolution of 256 × 256 and 1mm slice thickness). Resting-state functional MRI images were collected using an 8-minutes sequence (TR = 2000 ms, TE = 30 ms, flip angle = 78, FOV = 240 mm, slice thickness = 3 mm, 180 volumes). HC: MRI scans were acquired with a 3T Philips Intera Achieva scanner at Newcastle University. T1 weighted scans were obtained using a MPRAGE sequence (TR = 8.3 ms, TE = 4.6 ms, flip angle = 8, in-plane resolution = 1.0×1.0 mm2, slice thickness = 1.0 mm). Resting-state functional MRI images were obtained with a gradient echo echo-planar imaging sequence with 33 contiguous axial slices (TR = 2072 ms, TE = 30 ms, FOV = 192×192 mm2, in-plane resolution = 3.0×3.0 mm, slice thickness = 3 mm (1.0 mm gap), 290 volumes). To mitigate the confounding effect of unequal time-series length on fingerprinting metrics and to match the temporal resolution of the patients' cohort, we limited the analyses to the first 180 of the 290 acquired volumes.

Area of acquisition: Whole brain

Diffusion MRI: ☐ Used ☒ Not used

Preprocessing

Preprocessing software: fMRI data were preprocessed using in-house MATLAB code publicly available at https://github.com/juancarlosfarah/apero

Normalization: The data were not normalised (i.e., not brought into standard space).Instead, parcellation in MNI coordinates was first normalised to the individuals' previously T1 image and then resampled to the lower functional BOLD resolution. Final analyses were in native space.

Normalization template: Parcellation was in Montreal Neurological Institute (MNI) standard space

Noise and artifact removal: Image preprocessing pipelines for the two cohorts included substantially similar steps.These included: T1-weighted volumes were skull-stripped, segmented into white, grey matter and cerebrospinal fluid and spatially coregistered to fMRI native space. fMRI volumes were corrected for nuisance signals including motion signals, average white matter and cerebrospinal fluid signals, and band-pass filtered to the band 0.01-0.15 Hz.

Volume censoring: No volume censoring was applied.

Statistical modeling & inference

Model type and settings: None, not relevant.

Effect(s) tested: None, not relevant.

Specify type of analysis: ☐ Whole brain ☐ ROI-based ☒ Both

Anatomical location(s): *Describe how anatomical locations were determined (e.g. specify whether automated labeling algorithms or probabilistic atlases were used).*

Statistic type for inference (See Eklund et al. 2016): None, not relevant.

Correction: None, not relevant.

Models & analysis

n/a	Involved in the study
☐	☒ Functional and/or effective connectivity
☐	☒ Graph analysis
☒	☐ Multivariate modeling or predictive analysis

Functional and/or effective connectivity: We estimated individual FC matrices using Pearson's correlation coefficient between the averaged signals of all regions pairs.

Graph analysis: Functional connectome graph analysis, weighted undirected graph, at the individual and group level. Statistical analysis of group differences at the edge level, nodal strength analysis at the individual and group level
