# Supplementary material for: Patient-specific functional networks track early cognitive alterations and minor hallucinations in Parkinson’s disease
Source: Nat Ment Health. 2026 Jun 8;4(6):1021–34. doi: 10.1038/s44220-026-00657-x (PMC13652722; doi:10.1038/s44220-026-00657-x)
Supplement: Supplementary file 3 [file 44220_2026_657_MOESM3_ESM.pdf]

nature portfolio

Peer Review File

Patient-specific functional networks track early cognitive alterations and minor hallucinations in Parkinson's Disease

Corresponding Author: Dr Sara Stampacchia

Parts of this Peer Review File have been redacted as indicated to maintain the confidentiality of unpublished data.

This file contains all reviewer reports in order by version, followed by all author rebuttals in order by version.

Version 0:

Reviewer comments:

Reviewer #1

(Remarks to the Author)
Review of Connectome-based brain fingerprints predict early cognitive decline in Parkinson's patients with minor hallucinations

Stampacchia and colleagues explore the brain-fingerprints of minor hallucinations in Parkinson's disease. The authors use a rare sample of patients to explore a very interesting and timely question: Can we distinguish individuals with PD who experience minor hallucinations from those who do? They demonstrate that both groups of patients remain highly differentiable from one another and that the most salient features that differentiate individuals from one another can be used to predict clinical features. While the methods and questions of the paper are clear, the interpretation of the results could be greatly improved with additional analyses. I strongly believe that clarifying the interpretations of the findings, as suggested below, will improve the impact of the paper.

Major points:

1- In the introduction, the authors put forth the 'attentional network hypothesis' from the literature. It is unclear to me whether this hypothesis is specific to visual hallucinations or if it is similarly used to explain minor hallucinations in PD. Could the authors be explicit if they share the same hypothesis as described in the introduction (i.e., decreased DAN activation)? If so, how do their resting-state results align with this hypothesis, which is focused on task-based, ambiguous visual stimuli? I think it would be important to discuss this in greater detail and be more explicit about the hypothesis. This is particularly important because their results are seemingly opposed to the hypothesis: PD-MH show differentiable activity in the DAN network, and less differentiable activity in the SAL and DMN networks. This could suggest that the decoupling of the DAN network is person-specific, while the compensatory mechanisms of the SAL and DMN are not unique (i.e., common to the group).

2- The authors report in the introduction that: "While electrophysiological studies have reported preserved connectome identifiability in relation to motor impairment in PD 46,47".

Yet, the papers they cite to support this claim do not directly show this. Both previous papers on PD fingerprinting report lower differentiation of PD patients relative to HC, and between patients. The work by da Silva Castanheira et al suggest that this challenge in differentiating patients is specific to arrhythmic brain activity. Therefore, the authors should carefully nuance their discussion of previous literature and refine the interpretation of their results. The findings of the present paper are seemingly opposed to previous MEG PD-fingerprint work.

Despite this, I wish to emphasize that both findings can be true: i.e., differences in imaging modalities and different comparisons can be driving these diverging conclusions. Yet, the authors do not carefully and rigorously discuss this.

3- Although you show that each individual is differentiable from the others within each group, I wonder how discriminable individuals are from healthy controls or the other group (i.e., differentiating nMH-PD vs MH-PD)? This is particularly

important because the delta ICC results are interpreted as the most salient features that discriminate each group from one another, yet this is not the case. These are the brain-fingerprint features that maximally differ between groups (which is a related but slightly different interpretation). This should be clarified in the manuscript.

In a similar vein, one could also imagine an analysis where patients are compared to the nMH and MH groups as a form of preclinical assessment. Here, one would hypothesize that individuals more similar to the nMH group fingerprint would show a better overall clinical trajectory. I understand that there are many proposed analyses, but I would encourage the authors to think more carefully about whether their analyses specifically look at the differentiation between nMH and MH patients.

4- Dovetailing with the previous point, to make claims about the PD fingerprint, and specifically the MH-PD fingerprint, I believe that including a healthy control analysis is absolutely necessary. It is unclear which of the highlighted features that discriminate between nMH and MH also differentiate individuals from healthy controls. This analysis would clarify the interpretation of the results. For example, the authors assert: “First, we observed that brain-fingerprints are preserved in the presence of PD, respective of our patients’ hallucination status (Fig. 1)”. Yet to make this claim, the authors need to define the PD fingerprint relative to healthy controls. Based on previous studies in the field, and as far as I know, this has not been established in fRMI functional connectivity.

Imagine the scenario where the ICC nodes that maximally differentiate MH-PD from nMH, do not differentiate HC from MH-PD. In this case, it would be difficult to claim that this is a signature of minor hallucinations in PD if healthy controls are similarly differentiated from these features.

5- The analyses of Figure 4 are hard to interpret for me. Why did the authors explore the spatial alignment to the ICC maps independently for the MH-PD group and the nMH-PD group? This makes it very difficult to interpret. For example, the nMH group shows high spatial alignment with the D1 receptor, but this is not true for the MH group. The maps that show alignment to both groups are opposite in sign by definition. This is because delta ICC maps are not independent. I would encourage the authors to report a single correlation for the alignment with the delta ICC map, rather than the individual ICC maps of the most salient features of each group. I think this would greatly improve the interpretation of their findings.

6- While the authors aimed to explore subject-specific predictions and features, they defined ICC features at the group level (delta ICC map). While I do not disagree that these features are potentially interesting and related to participant differentiation, these are group-level effects. How do the authors reconcile that their interest is in defining person-specific predictions, yet the nodes used for each participant in their linear model are the same for every subject?

One way that the authors may be able to demonstrate the unique advantage of brain-fingerprinting approaches relative to other methods is to compare them to a linear model predicting clinical traits based on randomly selected nodes. In a similar vein, if you define group-level stat differences between nMH and MH, how different are these features to delta ICC? Can they equally be used to predict clinical traits as well as the brain-fingerprint approach?

I am also having a hard time reconciling the fact that these features were selected from the delta ICC maps. If the argument is that these features are patient-specific because they are based on ICC, they should be defined as features with the highest overall ICC within each group, rather than the largest difference between groups. The largest absolute ICC values for a group best differentiate individuals from one another. Large delta ICC, on the other hand, are features that greatly differ between the two groups, but may not differentiate the groups very well. Take, for example, the case where one group has an ICC of 0.1 and the second group an ICC of 0.35, the delta ICC would be very large, but the overall ability to differentiate individuals from this feature within the group is relatively low (ICC < 0.5).

7- “Here, we report for PD-MH patients a significant reduction in DMN and SAL identifiability (Fig. 2B and 2D), suggesting a more substantial loss of subject-specific features in these two key networks, potentially reflecting an early fragility/impairment of this group.” While it is true that the authors demonstrate that these features show reduced ICC for the PD-MH group relative to the PD-nMH group, whether these features are due to specific subject-specific loss (Iself) or whether they are due to an overall increase in the similarity to other individuals (Iothers) remains to be demonstrated. I would encourage the authors to explore the reason why they observed lower ICC in these specific networks with a more thorough analysis. I believe a more in-depth analysis will help buttress their claims.

8- The authors discuss the greater salience of the somatomotor network for differentiating PD-MH patients “The somatomotor network is typically not among those with the highest fingerprint in healthy subjects; however, in PD-MH, it stands out as one of the networks with the highest ICC, both within- network and in its connections with the DAN, SAL, limbic, and FPN networks (Fig. 2B).” They also make note of this in the abstract. However, the authors do not explicitly test how these effects compare to healthy controls; this inference is drawn based on qualitative comparisons to previous literature. It could stand that both groups show increased ICC relative to healthy controls, but the PD-MH group shows an overall increase beyond the general trend in PD (i.e., PD-MH >> PD-nMH >> HC). This interpretation would fit with a previous MEG brain-fingerprinting study (da Silva Castanheira et al., 2024). I would encourage the authors to either include a control group such that they can support their interpretation with additional data or alter their interpretation of the results.

9- Previous work on brain-fingerprinting in PD also shows that salient brain regions which differentiate individuals with PD from controls negatively colocalize with the distribution of 5-HT1A, 5-HT2A receptors (da Silva Castanheira et al., 2024). I would appreciate it if the authors could contextualize their results with the literature and suggest whether they believe their effects are a reflection of disease load, or are specific to the etiology of minor hallucinations.

10- It is very unclear to me what the authors are plotting in Figure 3C. Could the authors elaborate on how they got these plots and what they represent? How did the authors compute ‘patient-specific’ renders?

Minor points:

In the abstract, the authors report “we show that each patient exhibited a unique brain fingerprint, revealing rich quantifiable, personalized features with medical relevance”. I would encourage the authors to elaborate specifically on what they mean by medical relevance so that it is clear to readers how these features are clinically relevant.

The authors ran, if I understood correctly, six linear models. It is unclear, however, whether the authors corrected for multiple comparisons and whether they had any specific hypotheses for these analyses or if these tests were entirely exploratory.

There is a typo in Figure 3 caption there is no panel D in the figure.

I would appreciate it if the authors could elaborate on how they think their results can translate to individualized neuromodulation protocols (e.g., last paragraph of page 11).

I think the authors need to be more specific and explain which spatial autocorrelation-preserving permutation test they performed.

(Remarks on code availability)

Reviewer #2

(Remarks to the Author)
The authors report on functional connectivity patterns generated by resting state fMRI in a group of subjects with relatively early PD with or without minor hallucinations.
The subjects and their separation into two groups based on the presence or not of minor hallucinations has been reported in a prior publication and is not elaborated here, although Table 1 summarizes main demographic and clinical features; it is important that the two groups are not different in any one of these main variables.
Although the exact way in which the presence or absence of hallucinations was determined is apparently stated in a prior publication, it would be nice to elaborate on this a little further than what is stated here. Is this separation black and white? Are there levels of severity within the MH group and does this correlate with any of the variables studied here?
The authors convincingly show the ability to distinguish unique individual patterns of brain connectivity in each one of the subjects of the study, regardless of the group they belong to. This is an important finding.
The authors state: “Our results demonstrate that individual functional connectivity patterns contain stable, subject-specific features, enabling patient identification in PD”. Perhaps here they should specify that the evidence concerns early PD, as such individual patterns may be lost in advanced disease, which is not studied here.
They also show, in Figure 2, significant differences in functional connectivity patterns in specific brain regions between the two groups; it is very interesting that the high identifiability in the nH group is similar to reported healthy controls, while the individuals in the MH group are divergent. It would have been preferable to have controls within the present study to further substantiate this point.
The authors state: “In the present study, PD patients without hallucinations –who are less likely to experience cognitive decline maintain high identifiability relying upon the DMN, FPN and SAL (Fig. 2A).” FPN identifiability is substantially lower than in the MH group; this is discussed, but perhaps the above statement could be tempered. In any case, it is also very interesting that the DMN and SAL networks, differentially involved in the MH group, have also been implicated in more advanced cases with complex visual hallucinations.
The deficiencies in frontal-executive networks observed here in the MH group have been reported before, but the entirely data-driven approach here provides robust confirmation of earlier findings in a most authoritative fashion.
The findings with the correlation of apparent decreased receptor binding in areas of high fingerprint connectivity in the MH group are for the most part highly significant, surviving Bonferroni correction for multiple comparisons, and remarkably in the opposite direction in almost all cases in the nH group. It is quite impressive how the two groups move in opposite directions in this regard, but the interpretation of this obvious effect is not very clearly stated in the discussion.
The limitation section is appreciated.
Ref 10 is repeated as ref 30.
Overall, this is an extremely well substantiated and presented piece of work on an important topic, that of minor hallucinations in relatively early stages of PD, an intriguing feature of the disease. Methodology used is state of the art. The manuscript is written in a very organized fashion, facilitating readership despite its highly complex nature. It represents a significant addition to the field.

(Remarks on code availability)

Reviewer #3

(Remarks to the Author)
In this manuscript, the authors studied minor hallucinations in PD patients using connectome-based brain fingerprints technique and associated it with cognitive decline in PD patients. A total of 32 PD patients is included, with 18 of them have minor hallucinations and 14 of them having no hallucinations. This study is a re-analysis of a previously published study (Bejr-kasem et al., Mov. Disord. 2019). There are a few concerns about this study though:
1. The sample size is small for fingerprints analysis.
2. The scan length also is barely enough for robust functional connectome analysis and fingerprints validation.
3. No obvious group differences are detected by the major fingerprints-based indices. Most of results are descriptive.
4. Spatial specific measures detected some changes and clinical association in both PD-MH and PD-nH groups. It is hard to say what really are the real pathological changes leading to mental hallucinations in PD.
5. The in-house fMRI processing pipeline might reduce the reproducibility of this study.
6. Mental hallucination is defined by using one study visit based on patient recall. There is no consideration about the accuracy of mental hallucination diagnosis.

(Remarks on code availability)

Version 1:

Reviewer comments:

Reviewer #1

(Remarks to the Author)
Minor comments:

The sentence “whereas da Silva Castanheira et al. 29 replicated and extended these findings by showing that identifiability remained high in PD relative to healthy controls only when focusing on arrhythmic activity, predicting symptoms laterality.” Should read “when focusing on rhythmic activity.” The findings of da Silva Castanheira et al. (2024) demonstrated that PD patients remained differentiable from rhythmic brain activity, and were more challenging to differentiate from arrhythmic brain activity.

I would refrain from describing brain-fingerprinting as an identification process, it much better described as differentiation. Identification comes with specific connotations and may misguide readers, specially those who are not familiar with the field. I would therefore rephrase the paper accordingly to remain consistent with the rest of the field and avoid confusion. e.g., “identification of individuals from a population” and “reported decreased identifiability in PD vs. healthy controls predicting clinical motor impairment.”

Brain renders in Figure 3C: I am still a bit confused about the interpretation of the results presented in Figure 3C. From what I understand, these maps correspond to the strength of each FC edge identified by the ΔICC approach, and the strength (colour) of the edges is how much each individual ‘expresses’ that connection. If that is the case, then each participant should have the nodes in the same location across renders, and the only thing that should differ between renders is the colour across edges between participants. But this is not the case. Could the authors clarify this?

(Remarks on code availability)

Reviewer #2

(Remarks to the Author)

The authors have made a great effort to respond to the points made.
Their responses are satisfactory, and the resultant modifications have enriched the manuscriphallucinatory phenomena t.
I still find it somewhat counter-productive that they have based this whole very complex analysis on a biniary classification based on a single question of the MDS/UPDRS I and a "semi-structured" interview. There are a number of scales that assess in a structured fashion the intensity and frequency of hallucinatory phenomena that could have been used. In any case, the results show that even this rather crude classification works, so perhaps this is a mute point; it would be important in future work for the group if such tools were utilized.
In point 2, it should rather be "short" than "mild-to-moderate" disease duration.

(Remarks on code availability)

Reviewer #3

(Remarks to the Author)
The authors addressed part of my concerns regarding sample size and length of MRI scans. It is helpful to include controls in

this revision.
I am not convinced by their rebuttal regarding statistical concerns. There are no group difference between MH and nH patients in terms of primary fingerprint indices (ISelf, IOthers). ICC matrices collapse individual information into a group-wise measure. Therefore, it is merely a descriptive measure for each group. Spatial specificity (network) comparisons at group level are merely secondary analysis of ICC matrices property between groups. It cannot infer between subject group differences. I would suggest that author clearly state the negative findings in the abstract, tune down overall value of fingerprint, and focus on ICC matrics and its value in detecting network differences and correlating with clinical scores.
Despite lengthy description of ICC, it is not quite clear how it is calculated. Please revise and make it clear.
The spatial correlation between ICC maps and neurotransmitter maps is interesting. In method part, it states that group-specific ICC matrix was correlated with neurotransmitter maps. But only MH results are present in results section. Are there any significant correlations between ICC matrix from nH patients with neurotransmitter maps? Please also discuss how the correlations between spatial patterns from MH patients (the fingerprint) and normal controls (neurotransmitters) are meaningful to infer the reached conclusion.
A minor issue: please provide parallel MRI acquisition parameters for patients and controls along with scan time duration of each group, scanner software version.

(Remarks on code availability)

Version 2:

Reviewer comments:

Reviewer #1

(Remarks to the Author)
The authors have addressed all my concerns.

(Remarks on code availability)
I have not looked at their code.

Reviewer #2

(Remarks to the Author)
The authors have responded to the comments in a satisfactory fashion.

(Remarks on code availability)

Reviewer #3

(Remarks to the Author)
The authors have answered all my concerns.

(Remarks on code availability)

Open Access This Peer Review File is licensed under a Creative Commons Attribution 4.0 International License, which permits use, sharing, adaptation, distribution and reproduction in any medium or format, as long as you give appropriate credit to the original author(s) and the source, provide a link to the Creative Commons license, and indicate if changes were made.
In cases where reviewers are anonymous, credit should be given to 'Anonymous Referee' and the source.
The images or other third party material in this Peer Review File are included in the article's Creative Commons license, unless indicated otherwise in a credit line to the material. If material is not included in the article's Creative Commons license and your intended use is not permitted by statutory regulation or exceeds the permitted use, you will need to obtain permission directly from the copyright holder.
To view a copy of this license, visit https://creativecommons.org/licenses/by/4.0/

Detailed response to reviewers

We thank the editors and reviewers for their careful and constructive evaluation of our work. We have addressed below all conceptual and methodological concerns, including the additional analyses, clarifications in the introduction and discussion, and the points raised regarding study limitations.

Table of Contents

Detailed response to reviewers ***1***

Reviewer #1: (Remarks to the Author) **3**

Major point 1 - Attentional network hypothesis 3

Major point 2 – Correct reference to paper da Silva Castanheira 6

Major point 3 - ICC: On identifiability and differentiability 8

Major point 4a - Healthy Controls: preserved identifiability 10

Major point 4b - Healthy Controls: differentiation PD-MH vs HC 15

Major point 5 – Neurotransmitter Systems 19

Major point 6a – ICC at the group level, how is this patient-specific? 21

Major point 6b - Advantage of ICC against null and group-comparison in associating with clinical traits 22

Major point 7 – Delta ICC vs. Group ICC 26

Major point 8 – ICC and ISelf IOthers 30

Major point 9 – Differences in SMT networks with HC 31

Major point 10 – Serotonin receptors da Silva Castanheira 32

Major point 11 – Brain renders legend 34

Minor point 1 – medical relevance 35

Minor point 2 – multiple comparisons 37

Minor point 3 -Typo 37

Minor point 4 – Individualised neuromodulation 37

Minor point 5 – Spatial autocorrelation 38

Reviewer #2 (Remarks to the Author): **39**

Major point 1 - Classification MH vs. nH 39

Major point 2 – early vs. advanced stages of PD. 40

Major point 3 – Difference in ICC with HC 41

Major point 4 - Interpretation of network results 45

Major point 5 – Improve interpretation of neurotransmitter 47

Minor points 48

Reviewer #3 (Remarks to the Author): **49**

Major point 1 – Small sample size 49
Major point 2 – Scan length 50
Major point 3 - Results are descriptive 51
Major point 4 – Interpretation of spatial features findings....53
Major point 5 – Reproducibility of pre-processing pipeline 56
Major point 6 – Hallucinations Assessment....56

References: 58

Reviewer #1: (Remarks to the Author)

Review of Connectome-based brain fingerprints predict early cognitive decline in Parkinson's patients with minor hallucinations

Stampacchia and colleagues explore the brain-fingerprints of minor hallucinations in Parkinson's disease. The authors use a rare sample of patients to explore a very interesting and timely question: Can we distinguish individuals with PD who experience minor hallucinations from those who do? They demonstrate that both groups of patients remain highly differentiable from one another and that the most salient features that differentiate individuals from one another can be used to predict clinical features. While the methods and questions of the paper are clear, the interpretation of the results could be greatly improved with additional analyses. I strongly believe that clarifying the interpretations of the findings, as suggested below, will improve the impact of the paper.

We thank the reviewer for the positive assessment of our work and for highlighting the importance of our study. We appreciate the constructive comments and address each point in detail below. All changes in the manuscript are highlighted in blue and underlined.

Major point 1 - Attentional network hypothesis

In the introduction, the authors put forth the 'attentional network hypothesis' from the literature. It is unclear to me whether this hypothesis is specific to visual hallucinations or if it is similarly used to explain minor hallucinations in PD. Could the authors be explicit if they share the same hypothesis as described in the introduction (i.e., decreased DAN activation)? If so, how do their resting-state results align with this hypothesis, which is focused on task-based, ambiguous visual stimuli? I think it would be important to discuss this in greater detail and be more explicit about the hypothesis. This is particularly important because their results are seemingly opposed to the hypothesis: PD-MH show differentiable activity in the DAN network, and less differentiable activity in the SAL and DMN networks. This could suggest that the decoupling of the DAN network is person-specific, while the compensatory mechanisms of the SAL and DMN are not unique (i.e., common to the group).

We mentioned the attentional network hypothesis in the original version of the manuscript in the context of task-based activation studies and complex visual hallucinations, because past research on hallucinations in PD has focussed on complex visual hallucinations and targeted much less frequently

minor hallucinations in PD. There is now also recent evidence from both task and resting-state fMRI also in PD patients with MH, and although there is less overall agreement, there seems to be some overlap in terms of involved functions and brain regions. We discuss this more explicitly now the in the introduction.

We also note that our study was primarily exploratory, given the limited literature on MH and the use of connectome fingerprinting analyses. Therefore, we did not want to formulate specific hypotheses regarding the directionality of network differentiability. In addition, following additional analyses suggested by Reviewer 1 (see our response to your Major point 7 below), we observed that the DAN network no longer shows specific effects, suggesting that previous interpretations should be reconsidered. We agree that the relationship between MH and VH, as well as between resting-state activity, task-based findings, and fingerprinting, is complex. Accordingly, in the revised manuscript, we have clarified the literature in the introduction, and provided a clearer interpretation of the relationship between our results and prior studies in the discussion.

We have amended two paragraphs in the Introduction to provide greater clarity regarding the existing literature (p. 3). The revised text now reads as follows (changes in blue):

Most studies on the neural substrates of hallucinations in PD have focused on complex visual hallucinations, the most common type in advanced PD [1]. Activation studies suggest altered bottom-up and top-down visual processing in visual hallucinations, with reduced activation in primary visual cortices and increased activation in fronto-parietal regions during visual stimulation [2] and also during ongoing hallucinatory episodes [3]. Similarly, resting-state functional connectivity studies reveal convergent findings, with increased connectivity between sensory and default-mode regions, consistent with over-reliance on top-down signals (cf. [4] for a recent review). In line with these results, the attentional network hypothesis [5–7] proposes that hallucinations in PD arise from impaired recruitment of the dorsal attentional network (DAN) in response to ambiguous visual input, itself a consequence of abnormal bottom-up processing in early visual areas [8]. This failure to stimulus-driven control may bias perception towards internally driven interpretations, mediated by default mode (DMN) and/or salience (SAL) networks.

Although the majority of past imaging work has focused on visual hallucinations in PD, so-called ‘minor’ hallucinations (MH) – including presence hallucinations, passage hallucinations, and pareidolias [9,10] – are of clinical relevance, because they often occur at earlier stages of PD [11,12], may even precede parkinsonian motor symptoms [13] and occur in up to 40% of patients [14,15]. Brain alterations in MH have been found to partially overlap with those observed in complex visual hallucinations and are consistent with the attentional network hypothesis. Recent work revealed increased activation in the posterior middle-temporal gyrus, a hub at the intersection of the DMN, SAL, and DAN, and inferior

frontal gyrus (part of the DMN) during robotically-induced MH [16], as well as increased frontal EEG activity during rest [14]. Structurally, MHs are associated with gray matter reduction in visuo-temporal regions [17,18]. These studies have linked these brain alterations with mild cognitive impairment. Resting-state connectivity studies have reported increased connectivity within the DMN [19,20], reduced DAN connectivity, and disrupted interactions between the DAN and the DMN [19]. Although this body of research propose a convergent account on the neural basis of MHs and visual hallucinations in PD, it has primarily relied on group-averaged analyses, which do not capture the individual-specific nature of functional brain architecture – also known as the brain fingerprint [21,22]. Considering the heterogeneity and individually distinct phenomenological expression of hallucinations, accounting for neural variability is particularly crucial.

We have also amended the last paragraph of the Introduction, which introduces the study and its hypotheses (p. 4). The revised text now reads as follows:

If functional connectomes are unique to each individual, they may also encode person-specific features that capture clinically meaningful variability. This exploratory study aims to determine the extent to which group-level alterations previously reported in PD-MHs reflect consistent patterns across individuals versus idiosyncratic effects. To this end, we investigate, for the first time, whether connectome-based fingerprints derived from fMRI can identify patient-specific functional networks in PD, and whether there are patterns unique to PD patients with minor-hallucinations (PD-MH) relative to those without (PD-nH). We further examine whether these person-specific alterations are associated with clinical traits, thereby testing whether individual neural signatures correspond to clinically relevant variability. Given that hallucinations represent a major risk factor for dementia [23], we hypothesise that patient-specific networks in PD-MH are associated with early signs of cognitive impairment. Finally, we investigate whether brain regions with high inter-individual neural variability are associated with the cortical density of major neurotransmitters systems implicated with hallucinations. Previous studies have reported alterations in cholinergic, serotonergic, dopaminergic, and GABAergic systems in PD patients with hallucinations [24–26]. Understanding whether inter-individual variability in functional connectivity reflects the cortical distribution of specific neurotransmitter systems is of considerable interest, as it could reveal the neurochemical underpinnings of individual differences in brain organization.

We have also linked the results with existing literature in the discussion (p. 14). The revised text now reads as follows:

In healthy subjects, the most prominent networks for subject identification are the DMN, FPN and SAL [21,22], a pattern that has also been confirmed in cognitively healthy older adults in previous work[27] and found also in the present HC dataset (see Fig. S1). Here, PD-MH, relative to PD-nH, exhibited lower ΔICC in DMN-within-network connections, as well as in DMN-FPN, DMN-SAL, DMN-VIS and SAL-FPN edges (Fig. 2D), indicating diminished subject-specific features in these networks. This pattern aligns with the notion that PD-MH have a more severe and rapidly advancing form of PD often associated with cognitive impairments [12] and may reflect early network fragility or emerging impairment. PD-nH, who are less likely to experience cognitive decline [23], maintain a more stable, cognitively-healthy brain fingerprint anchored in these core systems. Relative to PD-nH, PD-MH patients also showed lower ΔICC also in subcortical regions, and their connection with DMN and FPN. Although speculative, these fingerprinting results are compatible with observations from both resting-state and task-based studies and the attentional network hypothesis. This hypothesis posits that hallucinations are associated with overactivation of the DMN and SAL networks, whose interaction may lead to failures in stimulus-driven control and a bias toward internally generated perceptions [8]. Such overactivation could be reflected in reduced reliability of these networks.

Major point 2 – Correct reference to paper da Silva Castanheira

- The authors report in the introduction that: "While electrophysiological studies have reported preserved connectome identifiability in relation to motor impairment in PD 46,47". Yet, the papers they cite to support this claim do not directly show this. Both previous papers on PD fingerprinting report lower differentiation of PD patients relative to HC, and between patients. The work by da Silva Castanheira et al suggest that this challenge in differentiating patients is specific to arrhythmic brain activity. Therefore, the authors should carefully nuance their discussion of previous literature and refine the interpretation of their results. The findings of the present paper are seemingly opposed to previous MEG PD-fingerprint work.

Despite this, I wish to emphasize that both findings can be true: i.e., differences in imaging modalities and different comparisons can be driving these diverging conclusions. Yet, the authors do not carefully and rigorously discuss this.

We thank the reviewer for this insightful comment and for allowing us to clarify this point. We are aware that the works by Troisi et al.[28] and da Silva Castanheira et al.[29] did not directly report maintained identifiability in PD. Specifically, Troisi et al.[28] found decreased identifiability using MEG in PD compared to healthy controls, while da Silva Castanheira et al.[29] replicated and extended these findings by showing that differentiability remained high in PD relative to healthy controls, but only for arrhythmic activity.

In a more recent preprint, da Silva Castanheira et al.[30] further demonstrated that arrhythmic components capture changes in identifiability across the lifespan, with children showing reduced identifiability in arrhythmic activity compared to adults. This is consistent with the idea that arrhythmic components may better capture individual-specific features later in life and/or in disease. In the same work, the authors also showed that rhythmic activity carries identifiable features across the lifespan. Overall, these findings suggest that the mechanisms driving identifiability may vary in health and disease (specifically PD).

We agree that the statement in the original Introduction could have been perceived as overly simplified and that this aspect was insufficiently discussed in the Discussion section. We have therefore revised both sections to better reflect the nuances of previous findings and to discuss more explicitly how methodological differences between electrophysiological and fMRI approaches may account for the apparent divergence between our results and previous MEG-based PD fingerprinting work.

The revised part of the Introduction and Discussion now reads as follows:

Introduction (p.4)

Functional connectivity patterns estimated from resting-state functional magnetic resonance imaging (fMRI) or magnetoencephalography (MEG) data – referred to as functional connectomes (FCs) – are unique to each individual [21,22,31]. These so-called *brain-fingerprints* allow to identify individuals from a population, have been linked to cognitive traits [32,33] and are altered in psychiatric [34–36] as well as neurodegenerative diseases, including Alzheimer's Disease [37,27] and PD [28,29]. An electrophysiological study (MEG) by Troisi et al., [28] reported decreased identifiability in PD vs. healthy controls predicting clinical motor impairment, whereas da Silva Castanheira et al. [29] replicated and extended these findings by showing that identifiability remained high in PD relative to healthy controls only when focusing on arrhythmic activity, predicting symptoms laterality. This indicates that some aspects of individual-specific connectivity may be preserved in PD, depending on the neural signal components examined. Building on these insights, the present study investigates this phenomenon for the first-time using fMRI, leveraging its higher spatial resolution, to examine whether fMRI connectome-based fingerprints can differentiate PD patients, and in particular, those with versus without minor hallucinations.

Discussion (p.12)

Our results demonstrate that individual functional connectivity patterns contain stable, subject-specific features, enabling patient identification in PD. These findings extend previous evidence from MEG studies in PD [28,29] and align with fMRI studies in other neurodegenerative disorders [27]. While previous MEG studies reported that identifiability in PD may vary depending on the neural signal component considered, the present fMRI results show that stable, individual-specific connectivity is detectable at

the macroscale. This suggests that the preservation of brain fingerprints in PD may reflect broader organizational properties, despite potentially different physiological underpinnings.

Major point 3 - ICC: On identifiability and differentiability

Although you show that each individual is differentiable from the others within each group, I wonder how discriminable individuals are from healthy controls or the other group (i.e., differentiating nMH-PD vs MH-PD)? This is particularly important because the delta ICC results are interpreted as the most salient features that discriminate each group from one another, yet this is not the case. These are the brain-fingerprint features that maximally differ between groups (which is a related but slightly different interpretation). This should be clarified in the manuscript.

In a similar vein, one could also imagine an analysis where patients are compared to the nMH and MH groups as a form of preclinical assessment. Here, one would hypothesize that individuals more similar to the nMH group fingerprint would show a better overall clinical trajectory. I understand that there are many proposed analyses, but I would encourage the authors to think more carefully about whether their analyses specifically look at the differentiation between nMH and MH patients.

We thank Reviewer 1 for highlighting the important distinction between, on one hand, identifiability and reliability, characterized by ICC, and on the other hand, differentiability. We agree that the ΔICC results identify FC features that are *most reliable* within groups, rather than directly reflecting between-group differentiability. This conceptual distinction has now been explicitly clarified in various parts of the revised manuscript.

Importantly, we show that ΔICC features exhibit significant between-group differences across several networks of interest (see updated network-level analyses, Major point 7 and Fig. 2D), supporting their potential relevance to serve as group-differentiating fingerprint features.

With respect to differentiating patients from healthy controls, as discussed extensively in our response to Major point 4, we do not have access to a healthy control dataset acquired at the same site and with the same protocol. Nevertheless, we performed additional analyses to assess whether ΔICC features reflect an MH-related signature rather than more general disease-related effects, which are discussed extensively in Major point 4 and now reported in the revised manuscript.

Finally, we fully agree that longitudinal analyses examining whether individuals whose brain fingerprints are more similar to those of the PD-nH group show a more favorable clinical trajectory would be highly informative, but such analyses require longitudinal data. This represents another active

direction of ongoing work in our group, and we now explicitly highlight it as a promising avenue for future research in the Discussion.

The revised parts of the Results, and Discussion sections now reads as follows:

Results; Page 6:

Analysing the local properties of the brain fingerprint using edgewise intra-class correlation (ICC, Amico & Goñi, 2018a), we observed that the functional connections with the highest fingerprint (i.e., the most reliable within groups) exhibited different spatial distributions in PD-nH, PD-MH and HC (Fig. 2A and Fig. S1). To identify ICC edges that differed between the PD patients with and without MH – i.e., features related to MH rather than general PD characteristics - we computed an edge-based differential identifiability (ΔICC) matrix (i.e., $ICC_{PD\text{-}MH} - ICC_{PD\text{-}nH}$; Fig. 2B, see 'MH-related differential ICC'). To test whether the features that most strongly differed in PD-MH versus PD-nH represented a signature of MH, rather than features generally associated with PD, we compared them with an edgewise differential identifiability matrix contrasting PD patients (PD-MH + PD-nH) with healthy controls (i.e., $ICC_{PD} - ICC_{HC}$). We found that only 0.5% of the top $\Delta ICC_{PD\text{-}MH - PD\text{-}nH}$ connections showed the same direction of effect and exceeded the minimal ΔICC value observed in PD-MH vs PD-nH (ICC = 0.5), suggesting that the edges with the highest $\Delta ICC_{PD\text{-}MH - PD\text{-}nH}$ values are largely related to MH (see Fig. S1). [...]

Results; Page 8:

In this analysis, we aimed to explore whether the functional connections that maximally differed in brain fingerprint features between PD-nH and PD-MH would associate with clinical scores in the two groups. To identify ICC edges that maximally differed between the two groups, we selected the top 0.1% ΔICC edges separately in PD-MH and PD-nH.

Legend Figure 3; Page 9:

The top delta ICC matrices were used as masks to isolate the functional connections that maximise differentiability in fingerprint between the two groups in each individual functional connectome.

Discussion; Page 10:

Second, we identified the spatial features of brain-fingerprints that maximally differed across the two groups (Fig. 2). Crucially, the spatial features of brain-fingerprints that differed most strongly between PD-MH and PD-nH also allowed us to identify patient-specific functional networks that associated with clinical traits at the individual level, differentiating cognitive symptoms in PD-MH from motor symptoms in PD-nH (Fig. 3).

Discussion; Page 12:

Taken together, these results suggest that MH in PD involves a dysfunction of frontal-executive and somatomotor networks, differentiating PD patients with versus without MH.

Discussion (p 17):

[..] Future work should explore how these patterns evolve alongside disease progression and whether they can be leveraged as early biomarkers for cognitive decline susceptibility in PD.

Major point 4a - Healthy Controls: preserved identifiability

Dovetailing with the previous point, to make claims about the PD fingerprint, and specifically the MH-PD fingerprint, I believe that including a healthy control analysis is absolutely necessary. It is unclear which of the highlighted features that discriminate between nMH and MH also differentiate individuals from healthy controls. This analysis would clarify the interpretation of the results. For example, the authors assert: "First, we observed that brain-fingerprints are preserved in the presence of PD, respective of our patients' hallucination status (Fig. 1)". Yet to make this claim, the authors need to define the PD fingerprint relative to healthy controls. Based on previous studies in the field, and as far as I know, this has not been established in fRMI functional connectivity.

Imagine the scenario where the ICC nodes that maximally differentiate MH-PD from nMH, do not differentiate HC from MH-PD. In this case, it would be difficult to claim that this is a signature of minor hallucinations in PD if healthy controls are similarly differentiated from these features.

We thank Reviewer 1 for raising these points. We start by responding to the first point (preserved identifiability in PD) and later (point 4b, p. 18) respond to the second point (differentiation PD-MH vs HC).

With regards to the claim that PD have preserved fingerprint, we agree that comparisons with healthy controls (HC) can provide additional insight; however, we emphasize that our PD patients showed a success rate of 100%, which is comparable to that reported for brain fingerprints in healthy populations in numerous previous studies, substantiating our claim that identifiability is preserved in PD. Nevertheless, we have carefully considered adding further comparison with HC: unfortunately, we do not have an HC dataset acquired at the same site and with the same MRI protocol as our PD cohort. Moreover, collecting new site-matched HC data is not feasible. While using external HC datasets is an option, within-subject (I-Self) and between-subject (I-Others, ICC) similarity measures are known to be sensitive to signal-to-noise ratio and scanner-specific factors [39], which would also limit the interpretability of direct quantitative cross-site comparisons.

Given these considerations, we have now performed new analyses on an independent HC dataset, where HC were matched for age, sex, and years of education with respect to our PD data set. A permutation-based ANOVA (5,000 permutations; I-Self/I-Others ~ Group + Age + Sex + Years of Education + Framewise Displacement + Site) comparing PD–MH, PD–nH, and HC showed no main effect of group, indicating that fingerprint identifiability does not differ significantly between PD and HC. Success-rate identifiability was 100% across all groups and datasets, **supporting once again our main conclusion that identifiability is preserved in PD,** regardless of hallucination status. We hope this answer the reviewer's first concern.

The revised parts of the Introduction, Methods, Results, Discussion and Supplementary Materials sections now reads as follows:

Introduction (p. 4):

[…] If functional connectomes are unique to each individual, they may also encode person-specific features that capture clinically meaningful variability. This exploratory study aims to determine the extent to which group-level alterations previously reported in PD-MHs reflect consistent patterns across individuals versus idiosyncratic effects. To this end, we investigate, for the first time, whether connectome-based fingerprints derived from fMRI can identify patient-specific functional networks in PD similarly to healthy controls (HC), and whether there are patterns unique to PD patients with minor-hallucinations (PD-MH) relative to those without (PD-nH). […]

Methods (p. 14):

Participants

N=32 patients with PD who previously participated in 37 and N=31 healthy controls (HC) were included in the analyses. […] HC were matched for age, sex and years of education, and collected at Newcastle University. […]

Image acquisition parameters

PD patients: MRI scans were acquired with a 3T Philips Achieva at the Hospital de la Santa Creu i Sant Pau in Barcelona. T1 weighted scans were obtained using a MPRAGE sequence (TR = 500 ms, TE = 50 ms, flip angle = 8, field of view [FOV] = 23 cm with in-plane resolution of 256 × 256 and 1mm slice thickness). Resting-state functional MRI images were collected using an 8-minutes sequence (TR = 2000 ms, TE = 30 ms, flip angle = 78, FOV = 240 mm, slice thickness = 3 mm, 180 volumes). HC: MRI scans were acquired with a 3T Philips Intera Achieva scanner at Newcastle University. T1 weighted scans were obtained using a MPRAGE sequence (TR = 8.3 ms, TE = 4.6 ms, flip angle = 8, in-plane resolution = 1.0 × 1.0 mm2, slice thickness = 1.0 mm). Resting-state functional MRI images were obtained with a gradient echo echo-planar imaging sequence with 33 contiguous axial slices (TR

= 2072 ms, TE = 30 ms, FOV = 192 × 192 mm2, in-plane resolution = 3.0 × 3.0 mm, slice thickness = 3 mm (1.0 mm gap), 290 volumes). To mitigate the confounding effect of unequal time-series length on fingerprinting metrics and to match the temporal resolution of the patients' cohort, we limited the analyses to the first 180 of the 290 acquired volumes.

Image processing

All subjects had ≤30% motion-tagged volumes. There was no significant difference across groups in the percentage of tagged volumes [p=.408] or DVARS [p=.520], but there was a significant difference in the average FD [p=.048], reflecting higher framewise displacement in HC relative to PD-nH [p=.042]. There was no difference across test and retest in the percentage of tagged volumes [p=.110] nor in DVARS [0.435] but there was a difference in the average FD [p=.047], revealing that on average subjects moved more in the second-half. As mentioned above, motion effects were already regressed out from the BOLD signal. Nonetheless, FD was additionally included as a covariate in analyses of fingerprinting metrics to further control for residual movement differences across groups.

Demographics

N=32 subjects with PD were included, of which N=18 with minor hallucinations (PD-MH) and N=14 without hallucinations (PD-nH). N=31 healthy controls were also included. Differences across groups were tested using Fisher's test for sex, and ANOVA (for PD-MH, PD-nH, HC comparisons), two-samples t-test (for PD-MH, PD-nH comparisons) or their non-parametric equivalent – i.e., Wilcoxon signed-rank and Kruskal-Wallis test – for the remaining variables. There were no differences across groups (i.e., PD-MH vs. PD-nH vs. HC) for sex (p=.652), age (p=.094) and years of education (p=.445). PD patients did not differ for motor symptoms severity (UPDRS III total: p=.238), cognitive functions (PD-CRS Frontal Subcortical: p=.848; PD-CRS Posterior: p=.875), disease duration (p=.253) and daily dose of L-dopa (p=.394) and dopamine agonist dose (p=.833; cf. Table 1).

Table 1. Demographics and clinical variables

	PD-MH	PD-nH	HC	p value	p value sig.
N	18	14	31	-	
Sex (%F)	44.4	28.6	32.3	0.652	ns
Age M(SD)	70.4(5.5)	65.8(7.8)	70.9(4.2)	0.094	ns
YoE M(SD)	12.5(4.6)	11.6(4.9)	13.7(2.69)	0.445	ns
Motor symptoms severity: UPDRS III - M(SD)	21.9(8.6)	25.8(9.2)	-	0.238	ns
Cognitive functions: PD-CRS Frontal max=104	63.3(14.9)	64.4(14.8)	-	0.848	ns
Cognitive functions: PD-CRS Posterior max=30	28.6(1.4)	28.6(1.7)	-	0.875	ns
Disease duration: years - M(SD)	5.2(3.8)	4(2)	-	0.253	ns

Daily dose of L-dopa	697.2(311.4)	601.1(311.9)	-	0.394	ns
Dopamine agonist dose	143.7(117.9)	153.6(139.9)	-	0.833	ns

Legend: PD-MH=Parkinson's disease with minor hallucinations; PD-nH= Parkinson's disease with no hallucinations; HC = Healthy Controls. M=Mean; SD=Standard deviation; Sex(%F) = % of Females; YoE=years of education; UPDRS=Unified Parkinson's disease rating scale; PD-CRS=Parkinson's disease - cognitive rating scale; p-value was estimated using Fisher's test for sex, and ANOVA (for PD-MH, PD-nH, HC comparisons), two-samples t-test (for PD-MH, PD-nH comparisons) or their non-parametric equivalent, i.e., Wilcoxon signed-rank test or Kruskal-Wallis test for the remaining variables. ns=not significant, p>0.05; *p≤0.05; ** p≤0.01;*** p≤0.001;**** p≤0.0001.

Results (p. 5):

Whole-brain within-sessions brain fingerprint

Investigating *whole-brain* fingerprint, we observed a 100% *Success-rate* in PD-MH, PD-nH and HC, indicating that identifiability is preserved in the presence of PD. In other words, as in healthy individuals, each patient could be correctly identified based solely on their FC (Fig. 1B, *ISelf* > *IOthers*: p<.001). IDiff was equally high in PD-MH and PD-nH (0.40), indicating strong individual brain discriminability in both groups. IDiff was slightly lower in HC (0.33), although differences in magnitude between PD and HC should be interpreted with caution given the potential variability in signal-to-noise ratio across acquisition sites. Test-retest reliability (ISelf) was high in the three groups, with no significant group differences after controlling for nuisance variables [ANOVA, 5000 permutations; F(2,53)=0.1, p=.605; PD-nH: M(SD)=0.67(0.06); PD-MH: M(SD)=0.68(0.1); HC: M(SD)=0.59(0.08); Fig. 1B]. Similarly, between-subjects similarity (IOthers) showed no group differences [ANOVA, 5000 permutations; F(2,53)=0.70, p=.0.248; PD-nH: M(SD)=0.27(0.03); PD-MH: M(SD)=0.27(0.04); HC: M(SD)=0.25(0.03)]. While no group differences were observed, some covariates (e.g., Age, FD) were significantly associated with IOthers (see Supplementary Table 1 for full statistics and additional analyses). This covariate effect was primarily driven by the HC group rather than the PD patients, as confirmed by supplementary analyses (cf. Supplementary Materials). Permutation testing confirmed that *IDiff* and *Success-rate* significantly deviated from null distributions (p <.0001) in both PD subtypes and HC alike (Fig. 1B). In sum, these findings demonstrate that fingerprinting can be applied in PD: patients can be individually identified with accuracy comparable to HC based solely on within-scan brain connectivity patterns, independent of clinical status, and significantly above surrogate null models.

Redacted

Figure 1. Within and between-subjects variability in functional connectivity. A) Functional connectome estimation at test (first half of the volumes) and re-test (second-half of the volumes). B) Identifiability matrices show within- (*ISelf*) and between-subjects (*IOthers*) test-retest reliability as Pearson correlation coefficient in PD patients without hallucinations (PD-nH), PD patients with minor hallucinations (PD-MH) and healthy controls (HC). Individuals' *ISelf* and *IOthers* are displayed, respectively, in the diagonal and off-diagonal elements of the matrix. The average ISelf, IDiff and Success-rate were similar in the three groups and IDiff and Success-rate differed at $p < .001$ (***) from random distributions. Boxplots shows that *ISelf* was significantly higher (paired-sample t-test, $p < .0001$****) than *IOthers* in all individual cases in PD-nH, PD-MH and HC.

Discussion (p. 11):

In this study, we analysed for the first time the features of brain-fingerprints using resting-state fMRI in patients with Parkinson's disease (PD) with minor hallucinations (PD-MH) and without any hallucinations (PD-nH). First, we observed that identifiability is preserved in the presence of PD, comparable to healthy controls (HC), and irrespective of our patients' hallucination status (Fig. 1).

Supplementary Materials (p. 1):

Additional Analyses: Whole-brain Fingerprinting

Full statistics ANOVA, 5000 permutations (IOthers and ISelf)

Supplementary Table 1. Differences across groups in *ISelf* and *IOthers*

ISelf *~ Group + Age + Sex + YoE + Site + ΔFD*

Anova Table	SS	df	F	parametric P(>F)	resampled P(>F)	p resampled signif.
Group	0.002	2	0.1	0.877	0.603	ns
Age	0.012	1	1.6	0.209	0.212	ns
Sex	0.010	1	1.4	0.243	0.244	ns
YoE	0.001	1	0.1	0.736	0.730	ns
Site	0.000	1	0.0	0.966	0.647	ns

ΔFD	0.002	1	0.2	0.632	0.632	ns

***IOthers** ~ Group + Age + Sex + YoE + Site +FD*

Anova Table	SS	df	F	parametric P(>F)	resampled P(>F)	p resampled signif
Group	0.001	2.0	0.7	0.502	0.257	ns
Age	0.006	1.0	6.3	0.015	0.015	*
Sex	0.000	1.0	0.0	0.861	0.856	ns
YoE	0.001	1.0	0.7	0.419	0.419	ns
Site	0.000	1.0	0.1	0.810	0.614	ns
FD	0.004	1.0	4.3	0.042	0.041	*

Legend: YoE=years of education; ΔFD=differential frame-wise displacement at test vs. retest; FD=average frame-wise displacement. p-value was estimated using ANOVA with 5000 permutations to control for sample size differences, after controlling for nuisances variables (see Table title for specs about the model). Resampling test using Freedman-Lane to handle nuisance variables and 5000 permutations; ns=not significant, p>0.05; *p≤0.05; ** p≤0.01;*** p≤0.001;**** p≤0.0001.

Covariate effects on between-subject similarity (IOthers)

To investigate the influence of potential confounding variables, we ran a linear model with *IOthers* as the dependent variable and Group, Age, Sex, YoE, Site, and FD as predictors . The model revealed that PD-MH showed slightly higher *IOthers* compared to HC (Estimate = 0.02; p = 0.033), while PD-nH was not significantly different from HC. Age was negatively associated with IOthers (Estimate = -0.002, p = 0.015), indicating that older participants tended to have slightly lower similarity with others. FD was positively associated with *IOthers* (Estimate = 0.093, p = 0.042), suggesting that motion has a small influence on similarity estimates. Sex, YoE, and Site were not significant predictors.

Analyses restricted to PD subgroups (PD-MH and PD-nH) confirmed that no significant group differences in IOthers were observed (F(1,26) = 0.95, p = 0.337), and none of the covariates (Age, Sex, YoE, FD) showed significant effects. This suggests that the covariate associations observed in the full model (notably for Age and FD) are mainly driven by the inclusion of HC participants, who were scanned at a different site, rather than by variability within the PD cohort.

Major point 4b - Healthy Controls: differentiation PD-MH vs HC

To address the second concern by Reviewer 1 – namely, whether the features that discriminate between PD-nH and PD-MH reflect a specific signature of MH or instead more general disease-related effects – we conducted the following additional analysis. We examined whether the connections with the strongest differential identifiability between PD-MH and PD-nH (i.e., $\Delta ICC_{PD\text{-}MH - PD\text{-}nH}$) overlapped with those with the strongest differential identifiability between PD and HC (i.e., $\Delta ICC_{PD - HC}$). Only 0.5% of the top $\Delta ICC_{PD\text{-}MH - PD\text{-}nH}$ edges showed both (i) the same direction of effect in PD–HC and (ii)

an absolute ΔICC value greater than the minimal ΔICC observed between PD-MH and PD-nH (ΔICC = 0.5). This indicates that the edges with the largest ΔICC differences between PD-MH and PD-nH predominantly capture MH-related alterations rather than general PD-related connectivity fingerprints.

The revised parts of the Methods, Results, and Supplementary Materials sections now reads as follows:

Methods (p.21):

***Minor Hallucination-related* differential ICC**

In these analyses, we aimed to identify edges that were MH-related, meaning those that contributed to differences in fingerprint between the PD patients with and without MH. To achieve this, we first computed a ΔICC matrix to capture MH-related differential identifiability, defined as:

$$\Delta\, ICC_{PD-MH-PD-nH} = ICC\ mat_{PD-MH} - ICC\ mat_{PD-nH}$$

As a result, the $\Delta ICC_{PD\text{-}MH - PD\text{-}nH}$ matrix (Fig. 2B) contained positive values for functional connections with higher ICC in PD-MH and negative values for those with higher ICC in PD-nH. Values close to zero indicated similar ICC across both groups, suggesting individual variability that did not contribute to distinguishing features between them. We identified group-specific ICC edges by selecting the positive or negative delta-ICC edges, for PD-MH and PD-nH respectively. To determine whether the features most strongly distinguishing PD-MH from PD-nH were MH-related rather than more generally associated with PD, we performed the following analysis. First, we computed a second, PD-specific, differential ICC map defined as:

$$\Delta\, ICC_{PD-HC} = \frac{ICC\ mat_{PD-MH} + ICC\ mat_{PD-nH}}{2} - ICC\ mat_{HC}$$

Next, from this $\Delta ICC_{PD\text{-}HC}$ map, we retained only edges whose absolute values exceeded the minimum absolute value within the top 10% of $\Delta ICC_{PD\text{-}MH - PD\text{-}nH}$ (shown in Fig. S1B), and whose sign matched the direction of $\Delta ICC_{PD\text{-}MH - PD\text{-}nH}$. This was done to test the extent to which PD–HC ICC differences followed the same directionality of the strongest PD-MH vs PD-nH ICC differences, thereby assessing whether edges most strongly associated with MH overlapped with those reflecting PD-specific alterations. Finally, we quantified the overlap (Fig. S1C).

Results (p.6):

Spatial specificity of brain fingerprint of minor hallucinations in PD

Analysing the local properties of the brain fingerprint using edgewise intra-class correlation (ICC, Amico & Goñi, 2018a), we observed that the functional connections with the highest fingerprint exhibited different spatial distributions in PD-nH, PD-MH and HC (Fig. 2A and Fig. S1). To identify ICC edges that differed between the PD patients with and without MH – i.e., features related to MH

rather than general PD characteristics - we computed an edge-based differential identifiability (ΔICC) matrix (i.e., $ICC_{PD-MH} - ICC_{PD-nH}$; Fig. 2B, see 'MH-related differential ICC'). To test whether the features that most strongly differentiated PD-MH from PD-nH represented a signature of MH, rather than features generally associated with PD, we compared them with an edgewise differential identifiability matrix contrasting PD patients (PD-MH + PD-nH) with healthy controls (i.e., $ICC_{PD} - ICC_{HC}$). We found that only 0.5% of the top $\Delta ICC_{PD-MH - PD-nH}$ connections showed the same direction of effect and exceeded the minimal ΔICC value observed in PD-MH vs PD-nH (ICC = 0.5), suggesting that the edges with the highest $\Delta ICC_{PD-MH - PD-nH}$ values are largely related to MH (see Fig. S1).

Supplementary Materials (p.6):

Minor Hallucination related differential identifiability

Redacted

Figure S1. Overlap between brain fingerprint features related to hallucinations and those differentiating Parkinson's disease patients from healthy controls. A) Edgewise intra-class correlations (ICC) matrices for Parkinson's Disease patients without hallucinations (PD-nH) and with minor hallucinations (PD-MH) and healthy controls (HC). ICC quantifies within-subjects similarity between test and retest for each edge (FC between 2 regions). ICC matrices for PD-nH and PD-MH are the same as those displayed in Figure 2A of the main text; they are reported here to allow visual comparison. We display edges with ICC between the 50th and the 95th percentile, across the 7 resting-state networks (RSN), subcortical and cerebellar regions. B) ΔICC matrix showing the difference in ICC between PD-MH and PD-nH, as in Figure 2B, here thresholded for the top 10% absolute values. C) ΔICC matrix showing the difference in ICC between PD patients (PD-MH and PD-nH combined) and HC, restricted to edges showing the same direction of effect and exceeding the minimal ΔICC value observed in the PD-MH vs PD-nH comparison (ΔICC = 0.5, where ICC is considered 'good' above 0.6[40]). This corresponded to 0.5% of the total number of top edges shown in B). A), B) and C) are ordered according the 7 RSNs, subcortical and cerebellar regions. VIS=Visual Network; SMT=Somatomotor Network; DA=Dorsal Attention Network; SA=Salience Network; L=Limbic Network; FPN=Fronto-Parietal Network; DMN=Default-Mode Network; SBC=Subcortical regions; CRB=Cerebellum.

Discussion (p. 13)

[..]

We note that only 0.5% of the top ΔICC PD-MH vs PD-nH edges overlapped with those differentiating PD from HC, confirming that these effects primarily reflect MH-specific features (see Fig. S1).

Major point 5 – Neurotransmitter Systems

The analyses of Figure 4 are hard to interpret for me. Why did the authors explore the spatial alignment to the ICC maps independently for the MH-PD group and the nMH-PD group? This makes it very difficult to interpret. For example, the nMH group shows high spatial alignment with the D1 receptor, but this is not true for the MH group. The maps that show alignment to both groups are opposite in sign by definition. This is because delta ICC maps are not independent. I would encourage the authors to report a single correlation for the alignment with the delta ICC map, rather than the individual ICC maps of the most salient features of each group. I think this would greatly improve the interpretation of their findings.

We thank Reviewer 1 for this comment. Our initial rationale for splitting the ΔICC between the two groups (PD-MH and PD-nH) was to show differences between the two groups. To this end, in the original manuscript, we plotted the *absolute* ΔICC values: thus, the highest values in the MH-PD group represented hallucination-related nodes, whereas the highest values in the nH-PD group reflected non-hallucination-related nodes. While this approach allowed us to visualise group-specific features, it led to additional comparisons and, as pointed out by the Reviewer, perhaps introduced unnecessary complexity and increased the burden of multiple-comparison correction.

We appreciate that a single correlation analysis based on the signed ΔICC map may provide a clearer and more intuitive interpretation, where positive ΔICC values directly correspond to hallucination-signature regions. Therefore, following this suggestion, we have revised the analyses and now report alignment with the signed ΔICC map only.

We also note that during this reanalysis, we identified and corrected an error in the spatial autocorrelation–correction step of our original code. We additionally changed the correlation to Spearman, which is more appropriate for non-normally distributed data, as some of the neuroreceptor maps. These updates led to minor numerical changes, but critically, the main findings remain robust: regions with higher hallucination signatures (i.e., higher ΔICC) continue to show lower densities of 5-HT2A, GABAa, and M1 receptors, all of which are implicated in the neurobiology of hallucinations.

The revised parts of the Methods, Results, and Discussion sections now read as follows:

Methods (p. 23):

[…] Each receptor map was parcellated using the 278 parcellation from [41] to match the ΔICC map. Spearman rank correlations were computed between each receptor map and the ΔICC map. To assess statistical significance while accounting for spatial autocorrelation, null distributions were generated by computing Spearman correlations between the ΔICC map and 1,000 spatially permuted surrogate receptor maps using Moran spectral randomization (using neuromaps.nulls.moran from neuromaps [42]. Two-tailed p-values were computed as the proportion of permuted correlations equal to or more extreme

than the observed correlation. To correct for multiple comparisons, we applied the false discovery rate (FDR) correction using the Benjamini-Hochberg procedure (multipletests function from the python statsmodels package).

Results (p. 11)

Cortical regions with stronger hallucination-specific fingerprint exhibit reduced serotonin, GABA, and acetylcholine receptor density.

We examined whether the topography of the hallucination-specific brain fingerprint aligned with the cortical distribution of dopamine, serotonin, GABA, and acetylcholine receptors, given their role in the neurobiology of hallucinations (Fig. 4). We found that connections with a stronger MH-related brain fingerprint showed significantly lower cortical density of 5-HT2A serotonin (FDR-p = .028), GABAa (FDR-p = .045) and M1 acetylcholine (FDR-p = .016) receptors.

Redacted

Figure 4. Association between hallucination-specific brain-fingerprint and neurotransmitter receptor density. Scatterplots show Spearman spatial correlations between ΔICC map (where higher values indicate connections with a hallucination-specific brain fingerprint) and cortical neurotransmitter receptor densities for dopamine, serotonin, GABA, and acetylcholine. Violin plots depict the observed Spearman correlation (red or black dot with dotted line) relative to a null distribution of correlations obtained by computing Spearman correlations between the ΔICC map and 1,000 spatially permuted surrogate receptor maps (Moran spectral randomization), which accounts for spatial autocorrelation. Red dots indicate that the observed correlation exceeds the surrogate distribution at $p < 0.05$. Reported p-values are FDR-corrected (ns = FDR-p < .05). Serotonin receptors include 5-HT1a and 5-HT2a. Dopamine receptors include D1 and D2 and DAT refers to the dopamine

transporter. GABA receptor refers to GABAa. Acetylcholine receptors include M1 (muscarinic) and α4β2 (nicotinic). Abbreviations: BPnd = binding potential; SUVR = standardized uptake value ratio; VT = free-fraction corrected distribution volume.

Discussion (p. 15)

Finally, our analyses revealed that functional connections with stronger MH-related brain fingerprint (i.e., higher ΔICC in PD-MH) were associated with lower cortical density of serotonin (5-HT2A), GABAa and acetylcholine muscarinic M1 receptors. In contrast, patients without hallucinations in daily life (PD-nH) exhibited higher densities of these same receptors in regions with stronger differential fingerprint. No significant associations were observed with dopaminergic receptors (D1, D2, DAT), consistent with the fact that ΔICC primarily captures differences between PD subtypes, both of which share dopaminergic alterations. These findings also align with previous work on complex visual hallucinations [43], emphasizing the role of non-dopaminergic neurotransmitters in PD-related hallucinations. Previous studies have reported increased 5-HT2A receptor binding in the inferolateral temporal cortex in post-mortem PD patients with hallucinations [44], and in vivo studies have shown higher binding in the visual, dorsolateral, medial-orbitofrontal cortices, and insula in hallucinating PD patients [25]. Similarly, occipital GABAa reduction [26], and muscarinic receptor blockade [45], along with grey matter loss in cholinergic regions [46] have been linked to hallucinations. Notably, these regions do not overlap with the MH-related brain-fingerprint areas, where we observed lower receptor densities, suggesting that these high-differential-fingerprint regions may be relatively spared from hallucination-related alterations. Although speculative, this suggests that the within-session stability of connectivity captured by brain-fingerprint ICC could reflect compensatory mechanisms.

Major point 6a – ICC at the group level, how is this patient-specific?

While the authors aimed to explore subject-specific predictions and features, they defined ICC features at the group level (delta ICC map). While I do not disagree that these features are potentially interesting and related to participant differentiation, these are group-level effects. How do the authors reconcile that their interest is in defining person-specific predictions, yet the nodes used for each participant in their linear model are the same for every subject?

We thank the reviewer for this helpful comment and for allowing us to deepen this aspect. The edges selected via ICC are the same for each subject within a group; however, their connectivity strength differs across individuals, and this individual-specific component is incorporated into the model. ICC highlights connections that show stable test–retest patterns within a group, but it does not constrain the magnitude or direction of connectivity at the individual level: participants can show positive, negative, or absent connectivity along these edges.

We compute ICC separately for each clinical group because our aim is to identify connections that reliably drive the brain-fingerprint within the population of interest (PD-MH or PD-nH). By comparing

the ICC maps across groups and estimating the differential ICC (ΔICC), we isolate the connections whose fingerprint differs most between PD with or without hallucinations. These features isolate MH-related features common across all subjects, but each participant expresses them through their own individual connectivity strengths, making them simultaneously symptom-specific and subject-specific.

There could indeed be alternative approaches that select features in a fully individualised manner, independent of diagnostic grouping, for example, based solely on associations with behaviour. However, this is beyond the scope of the present work. Our aim was to leverage diagnostic-class information to identify condition-relevant features, and then to use these features to derive individualized predictions within each diagnostic group. We added a brief paragraph addressing this consideration to the Discussion section.

The revised parts of the Discussion now read as follows:

Discussion (p. 15):

[…]

Given these findings, it is important to clarify how our feature-selection approach captures individualized subject-specific information, while leveraging symptom-level distinctions. The ICC-derived features are defined at the group level. Although the set of ICC-selected connections is identical for all individuals within a clinical group, each participant expresses these features through their own connectivity strengths, which remain fully subject-specific. Group-level ICC serves to identify connections that show reliable test–retest FC patterns in PD-MH and PD-nH patients, and ΔICC isolates those with the largest stability differences between groups. MH-related connections, while still leveraging the individual variability in connectivity strength for personalized prediction. In other words, the present work shows a method to derive individualized alterations among individuals sharing the same symptom/clinical features.

[…]

Major point 6b - Advantage of ICC against null and group-comparison in associating with clinical traits

One way that the authors may be able to demonstrate the unique advantage of brain-fingerprinting approaches relative to other methods is to compare them to a linear model predicting clinical traits based on randomly selected nodes.
In a similar vein, if you define group-level stat differences between nMH and MH, how different are these features to delta ICC? Can they equally be used to predict clinical traits as well as the brain-fingerprint approach?

We thank the reviewer for these insightful suggestions. To address these points, we conducted two complementary analyses comparing the linear models based on fingerprinting-derived features versus: (1) features derived from the whole brain, and (2) features selected based on group-level statistical differences between PD-MH and PD-nH, in terms of their ability to account for inter-individual variability in clinical outcomes.
For the whole-brain analysis, we derived composite FC scores by aggregating positive and negative features across the entire connectome. For the group-level approach, we performed two-sample *t*-tests on all functional connectivity edges between PD-MH and PD-nH, and selected edges that exceeded the 99.9th percentile (as in ΔICC-based analyses) of the *t*-value distribution to mask FC features. In both cases, a composite FC score was computed for each individual and entered into a linear model predicting clinical outcomes (Clinical score ~ FC + age + years of education + disease duration), following the same procedure used for the ΔICC-based analyses.
These new analyses revealed the following: In contrast to the ΔICC-selected edges, which showed significant associations with clinical severity, neither the whole-brain features nor the group-difference–based features yielded significant relationships with any clinical measure (all $p > 0.05$). These findings indicate that neither unselected whole-brain connectivity nor features defined solely by group-level differences adequately capture the individual-level variability relevant to symptom expression.
Taken together, these results support the notion that the brain-fingerprinting approach – by emphasizing subject-specific stability of functional connectivity – encodes clinically meaningful information that is not captured by unsupervised whole-brain features or conventional group-level statistics. We have incorporated these results and their interpretation at several points in the revised manuscript.

The revised parts of the Methods, Results and Discussion now read as follows:
Methods (p. 22)

Associating clinical scores with brain fingerprint features
In these analyses, we tested whether clinical scores were associated with individuals' functional connections that most strongly distinguished the two groups in terms of fingerprint. To identify these key connections, we selected in the top 0.1th percentile edges for each group-specific ICC matrix. These edges were visualized on the cortical surface using BrainNet [47] (Fig. 3A). The binarized matrices served

as masks to extract high-fingerprint edges from each individual's functional connectome (Fig. 3C). For each individual, we separated positive and negative functional connections to prevent mathematical cancellation of connectivity values. We then computed two summed connectivity scores: one for positive and one for negative connections. Next, we fitted linear regression models (using MATLAB's fitlm function) to examine the relationship between clinical scores based and individual functional connectivity (Fig. 3B). Each model included a clinical score as the dependent variable and a functional connectivity value for each individual as the predictor, while controlling for age, years of education, and disease duration. In addition to ICC-based selection, we repeated the regression analyses using edges showing significant between-group differences identified via two-sample t-tests (by selecting the top 0.1% of edges with the highest t values for each group), as well as using the full functional connectome without any feature selection. The same modelling approach and covariates were applied in all analyses. These complementary analyses allowed us to test whether the brain-fingerprinting approach is advantageous over different edge selection approaches, including data-driven group differences and the full connectome.

The clinical scores analysed included the Unified Parkinson's Disease Rating Scale (UPDRS) III total, a global measure of motor symptom severity [48]. We also examined the PD-CRS Frontal Subcortical score, a composite measure of executive function that includes sustained attention, working memory, verbal fluency, clock drawing, and verbal memory tasks. This score is sensitive to mild cognitive impairment in PD [49] and has been linked to neural alterations specific to PD patients with MH [14,16]. In contrast, the PD-CRS Posterior score, which assesses confrontation naming and clock copying, is reportedly altered later, in the transition to dementia [49].

Results (p. 7)

Functional connections with high fingerprint are associated with clinical scores

In this analysis, we aimed to explore whether the functional connections that maximally differed in brain fingerprint features between PD-nH and PD-MH were associated with clinical scores in the two groups. To identify ICC edges that maximally differed between the two groups, we selected the top 0.1% edges separately in PD-MH and PD-nH. Their spatial distribution on the cortical surface is shown in Fig. 3A for both groups separately. These top ΔICC edges were then used to isolate functional connections with the highest edgewise differential identifiability in each individual's functional connectome, from which we derived a sum score separately for positive and negative connections. Using linear models, we tested whether this functional connectivity score was related to inter-individual variability in clinical scores of (i) motor symptoms severity (using UPDRS III total score), (ii) frontal-subcortical executive functioning (using PD-CRS frontal subcortical score) and (iii) posterior-cortical functions (using PD-CRS posterior score), separately in each group.

In PD-nH, negative connectivity in high-ΔICC edges was significantly associated with UPDRS III total score (Adjusted $R^2 = 0.65$; $p = .003$; Fig. 3B), indicating that more significant disconnection of fingerprinting hubs (individual brain renders, Fig. 3C) was associated with lower motor impairment. This differed for PD-MH, in whom functional connectivity was associated with the PD-CRS Frontal Subcortical neuropsychological score (Adjusted $R^2 = 0.64$; $p = .010$; Fig. 3B), with higher connectivity between fingerprinting hubs (Fig. 3C) linked to less executive impairment. No significant associations were found for the PD-CRS posterior score in neither group, which was consistent with the early disease stage. For transparency, we report both uncorrected and FDR-corrected p-values. After FDR correction, some results were marginally significant (e.g., PD-nH: $p = .003$, p FDR-corrected = .031; PD-MH: $p = .010$, p FDR-corrected = .059). Given the exploratory nature of these analyses, we emphasize that they are primarily hypothesis-generating rather than confirmatory, as fingerprinting is a relatively novel approach and we did not pre-register specific edgewise hypotheses.
Notably, neither the top 0.1% edges selected based on t-tests nor edges from the full connectome were associated with any of the clinical measures in either group, indicating that ICC-based edge selection identifies functionally relevant connections not captured by standard group-difference statistics nor when using unselected individualised edges.

Discussion (p. 14)

These differential results across the two patient groups were further supported by the finding that the identification of patient-specific functional networks was associated with different clinical traits – a result observed only when using ΔICC-based features selection, and not when using group-level t-test differences. This suggests that ΔICC-based and t-test–based feature selections tap into complementary aspects of the functional architecture: the former identifies stable, fingerprint-like connections that relate to clinical variation, whereas the latter isolates edges with maximally different mean connectivity between groups, which, however, are not associated with clinical scores. Similarly, unconstrained models using all possible connections failed to show significant association with clinical outcomes, highlighting the relevance of ΔICC-based, fingerprinting-derived features in their association with clinical outcomes. In PD-MH patients, stronger connectivity in these regions was associated with lower scores for frontal-subcortical functions (PDCRS score). Thus, although early PD-MH patients (including the present sample) generally have normal cognitive scores, they may show subtle sub-clinical deficits in frontal-subcortical functions (including executive and attentional functions [14,50]). Standard resting-state fMRI analyses at the group level reported that altered connectivity in a priori defined fronto-parietal connections in PD-MH was associated with frontal-subcortical scores [16]. The present study significantly extends these findings by showing that individualized functional connectivity patterns – rather than group-level effects – can be derived in an entirely data-driven manner and are detected in PD-MH patients with early sub-clinical cognitive alterations. This concerned only

frontal-subcortical functions and was not observed in PD-nH patients. Taken together, these results suggest that MH in PD involves a dysfunction of frontal-executive and cerebellar connectivity, differentiating PD patients with versus without MH. In contrast, for PD-nH patients – who typically show no or less rapid cognitive decline – we found that greater disconnection in regions with high fingerprint was associated instead with lower motor functions (UPDRS-III total score), further distinguishing a motor-only form of PD from a motor-cognitive form of PD. This may allow early distinction between both groups and different therapeutical strategies, as recent work has revealed different clinical trajectories and different sensitivities to disease-modifying treatments [51].

Major point 7 – Delta ICC vs. Group ICC

I am also having a hard time reconciling the fact that these features were selected from the delta ICC maps. If the argument is that these features are patient-specific because they are based on ICC, they should be defined as features with the highest overall ICC within each group, rather than the largest difference between groups. The largest absolute ICC values for a group best differentiate individuals from one another. Large delta ICC, on the other hand, are features that greatly differ between the two groups, but may not differentiate the groups very well. Take, for example, the case where one group has an ICC of 0.1 and the second group an ICC of 0.35, the delta ICC would be very large, but the overall ability to differentiate individuals from this feature within the group is relatively low (ICC < 0.5).

Our rationale for using ΔICC rather than absolute ICC within each group is that selecting features with the highest ICC per group would likely capture characteristics driven by general PD-related features (e.g., motor symptoms or other common PD manifestations), rather than features specific to MH.

To focus on MH-specific features – the only clinical difference between our two well-matched groups – we computed ΔICC. For behavioural association we retained only edges with extreme ΔICC values, applying minimum absolute thresholds of ΔICC=0.96 (MH) or ΔICC=0.85 (nH), (99.9th percentile). With these thresholds, cases such as the one proposed by Reviewer 1 (G1: ICC = 0.10; G2: ICC = 0.35; ΔICC = −0.25) are excluded, as only features with substantial and meaningful ΔICC values are considered.

The only exception was the network-level analyses, where we did not apply any thresholds. We therefore ran additional analyses applying different thresholds (97th, 98th, 99th percentiles, corresponding respectively to |ΔICC| MH=0.55 and |ΔICC| nH=0.48; |ΔICC| MH=0.61 and |ΔICC| nH=0.53; |ΔICC| MH=0.70 and |ΔICC| nH=0.61), ensuring that all included edges are based on fair group ICC values (ICC > =0.5, cf. Cicchetti and Sparrow, 1981). We also extended the analyses to between-networks to

provide an overall view. Results slightly varied at different thresholds, but the patterns were essentially maintained, confirming the robustness of our results, and have been added to the revised manuscript.

The revised parts of the Methods, Results and Discussion now read as follows:

Methods (p.22)

Finally, we examined the distribution of the MH-related ICC edges across functional networks. Specifically, we computed the percentage of edges per network exceeding ΔICC = ±~0.5 (corresponding to 97th percentile and considered to reflect "fair" ICC [40]) separately for PD-MH and PD-nH and tested for group differences using a binomial test (function binocdf in Matlab), applying FDR correction for multiple comparisons (Fig. 2D). Results for analyses without thresholding, as well as for higher thresholds (98th and 99th percentiles), are reported in the Supplementary Materials.

Results (p.6)

[..] The MH-related differential ICC edges ($\Delta ICC_{PD\text{-}MH\ -\ PD\text{-}nH}$) were then mapped onto the cortical surface as nodal strength (Fig. 2C) and the highest differences across the 7 resting-state networks (RSN), subcortical and cerebellar regions were identified (Fig. 2D). This analysis revealed differences between PD-MH and PD-nH. PD-MH showed a higher proportion of high ΔICC edges primarily involving the fronto-parietal network (FPN), including its connections with the dorsal attention network (DAN) and the cerebellum (CRB), as well as within-FPN networks connections. Additional differences were observed in several cerebellar pairs (CRB-CBR; CRB-DAN and CRB with visual network, VIS) and networks connected with the somatomotor network (SMT; Limbic-SMT, CRB-SMT).

In contrast, PD-nH showed a higher proportion of high-ΔICC edges primarily involving the default mode network (DMN), including its connection with the salience (SAL) and VIS, as well as within-network DMN connections. This was followed by the subcortical network (SBC) pairs, specifically SBC-FPN and SBC-DMN. Additional differences were observed in SAL connections (SAL-VIS, SA-FPN) and SMT–VIS connections (Fig. 2D). Supplementary Figure S2 shows that these patterns are largely maintained across different ΔICC thresholds (no threshold, 97th, 98th, 99th percentiles). In sum, these findings indicate that brain fingerprint is expressed through distinct functional connections in PD patients with (PD-MH) and without (PD-nH) minor hallucinations.

Redacted

Figure 2. FC patterns making subjects identifiable differ in PD-MH and PD-nH and allow between-group classification. […] D) Matrix illustrating network pairs that show significant differences in the proportion of high-ΔICC edges (i.e., hallucination-specific signature) between the two groups. High ΔICC edges are defined as those exceeding the 97th percentile within each group (approximately corresponding to ΔICC ~ ±0.5, where ICC = 0.5 is typically interpreted as "fair" [40]). Results are presented for all 7RSNs subcortical and cerebellar regions, including both within-network and between-network connections. Comparisons across groups are done using binomial test and corrected for multiple comparisons (FDR). Coloured cells indicate network pairs with significant group differences, with the FDR-corrected p value reported; ns = $p > 0.05$. Colours reflect the directionality of the effect, with blue indicating a higher proportion of high-ΔICC edges in PD-nH and red indicating a higher proportion in PD-MH.

Supplementary Materials (p.3)

Network differences in hallucination-related brain fingerprint in PD

Figure S2. Network differences in hallucination-related brain fingerprint in PD. Matrices show network pairs with significant differences in the proportion of ΔICC edges (i.e., the hallucination-specific signature) between PD-MH and PD-nH. This figure supplements Fig. 2D of the manuscript, demonstrating that these patterns are maintained across different thresholds. Panels are arranged from left to right: no threshold in ΔICC; threshold = 97th percentile; threshold = 98th percentile; threshold = 99th percentile. High ΔICC edges are defined as those exceeding the 97th percentile within each group (approximately corresponding to ΔICC ~ ±0.5, where ICC = 0.5 is typically interpreted as "fair", Cicchetti & Sparrow, 1981). Results are shown for all seven resting-state networks (7RSNs), subcortical, and cerebellar regions, including both within- and between-network connections. Group comparisons were performed using a binomial test and corrected for multiple comparisons using FDR. Colored cells indicate network pairs with significant group differences, with the FDR-corrected p-value reported; ns = $p > 0.05$. Colors reflect the directionality of the effect: blue indicates a higher proportion of high-ΔICC edges in PD-nH, and red indicates a higher proportion in PD-MH. VIS=Visual Network; SMT=Somatomotor Network; DA=Dorsal Attention Network; SA=Salience Network; L=Limbic Network; FPN=Fronto-Parietal Network; DMN=Default-Mode Network; SBC=Subcortical regions; CRB=Cerebellum.

Discussion (p.13)

In healthy subjects, the most prominent networks for subject identification are the DMN, FPN and SAL [21,22], a pattern that has also been confirmed in cognitively healthy older adults in previous work [27] and found also in the present HC dataset (see Fig. S1). Here, PD-MH, relative to PD-nH, exhibited lower ΔICC in DMN-within-network connections, as well as in DMN-FPN, DMN-SAL, DMN-VIS and SAL-FPN edges (Fig. 2D), indicating diminished subject-specific features in these networks. This pattern aligns with the notion that PD-MH have a more severe and rapidly advancing form of PD often associated with cognitive impairments [12] and may reflect early network fragility or emerging impairment. PD-nH, who are less likely to experience cognitive decline [23], maintain a more stable, cognitively-healthy brain fingerprint anchored in these core systems. Relative to PD-nH, PD-MH patients also showed lower ΔICC also in subcortical regions, and their connection with DMN and FPN, suggesting that subcortical pathology is also more diffuse in PD-MH. Although speculative, these fingerprinting results are compatible with observations from both resting-state and task-based studies and the attentional network hypothesis. This hypothesis posits that hallucinations are associated with overactivation of the DMN and SAL networks, whose interaction may lead to failures in stimulus-driven control and a bias toward internally generated perceptions [8]. Such overactivation could be reflected in reduced reliability of these networks.

By contrast, PD-MH patients exhibited a stronger ΔICC in FPN within-network connections, as well as FPN-DAN and FPN-cerebellum connections. Qualitatively, FPN ICC values also appeared higher in PD-MH than in HC (Fig. S1). Even though it is difficult to reconcile fingerprint results with findings from activation studies, our results are compatible with evidence of increased frontal theta activity in patients with MH, associated with lower cognitive functioning in an earlier EEG study [14], and potentially reflecting early compensatory mechanisms.

We also observed increased ΔICC in within-cerebellar pairs, as well as in its connectivity with DAN, SMT and VIS network. Qualitatively, cerebellar ICC also appeared higher in PD-MH than in HC (Fig. S1). Cerebellar lesions have been associated with hallucinations in a cohort of >80 patients [52], and reduction in grey matter volume in the cerebellum has been implicated in MH in a previous study [53]. As argued in recent work [54], there is a significant number of findings supporting the role of the cerebellum in psychosis, and specifically on its generalized role in the construction and adaption of internal models integrating sensorimotor inputs and cognitive control. This is in line with previously proposed accounts on the importance of the somatosensory and motor functions and the integration of these signals in the pathogenesis of MH, including presence hallucinations, which have recently been linked to specific somatomotor processes in PD patients [16] and in healthy subjects in whom presence hallucinations have been induced experimentally using a somatomotor conflicts (based on tactile, motor, and proprioceptive signals) [55], associated with activation of primary motor and premotor cortex (cf. Figure 2E in [16]).

Major point 8 – ICC and ISelf IOthers

"Here, we report for PD-MH patients a significant reduction in DMN and SAL identifiability (Fig. 2B and 2D), suggesting a more substantial loss of subject-specific features in these two key networks, potentially reflecting an early fragility/impairment of this group." While it is true that the authors demonstrate that these features show reduced ICC for the PD-MH group relative to the PD-nMH group, whether these features are due to specific subject-specific loss (Iself) or whether they are due to an overall increase in the similarity to other individuals (Iothers) remains to be demonstrated. I would encourage the authors to explore the reason why they observed lower ICC in these specific networks with a more thorough analysis. I believe a more in-depth analysis will help buttress their claims.

We thank Reviewer 1 for this comment. We would like to clarify that the ICC metric inherently captures the balance between Iself and Iothers: a high ICC corresponds to high within-subject consistency (Iself) and low between-subject similarity (Iothers). Conversely, a low ICC reflects reduced within-subject consistency, which may or may not be accompanied by changes in between-subject similarity. Therefore, the observed reduction in ICC in the identified networks for PD-MH patients directly reflects a loss of subject-specific features (i.e., features that do not exhibit reliable test-retest functional connectivity over time, equivalent – conceptually – to a reduction in Iself).

While further analyses of Iself and Iothers could be performed, we believe that these would risk complicating interpretation. For completeness, we provide here an exploration of Iself and Iothers within each network, but we prefer not to add these to the manuscript or supplementary materials. As shown in the present study (Fig. 2D), in networks with lower ICC for MH (i.e., DMN and SBC), nH subjects show higher Iself, although these differences are not statistically significant. Similarly, in networks with higher ICC for MH (i.e., CRB, FPN, and SMT), Iself is on average higher for MH, again without significance. The lack of significance may stem from the fact that correlation values here are computed across all edges in the network, whereas ICC is computed at the edgewise level. Our subsequent analyses examine whether one group has a higher number of edges with high ICC, providing a more detailed and edge-specific assessment than simple network-wide averages.

We consider that including these analyses does not substantially add to the current manuscript, as the edgewise ICC already provides a more detailed assessment. While this exploratory analysis highlights some differences in Iothers (between-subject variability), investigating such variability is outside the scope of the present study, which focuses on "fingerprintable" features. We are planning to address between-subject variability in a dedicated forthcoming publication.

Redacted

Major point 9 – Differences in SMT networks with HC

The authors discuss the greater salience of the somatomotor network for differentiating PD-MH patients "The somatomotor network is typically not among those with the highest fingerprint in healthy subjects; however, in PD-MH, it stands out as one of the networks with the highest ICC, both within- network and in its connections with the DAN, SAL, limbic, and FPN networks (Fig. 2B)." They also make note of this in the abstract. However, the authors do not explicitly test how these effects compare to healthy controls; this inference is drawn based on qualitative comparisons to previous literature. It could stand that both groups show increased ICC relative to healthy controls, but the PD-MH group shows an overall increase beyond the general trend in PD (i.e., PD-MH >> PD-nMH >> HC). This interpretation would fit with a previous MEG brain-fingerprinting study (da Silva Castanheira et al., 2024). I would encourage the authors to either include a control group such that they can support their interpretation with additional data or alter their interpretation of the results.

We note that the SMT shows relatively high ICC not only in PD-MH patients, but also qualitatively in older healthy controls - both in the controls analyzed here (see Fig. S1A) and in a previous fMRI dataset we published (Stampacchia et al., [27]). These data suggest that stronger SMT fMRI-fingerprinting may be a characteristic feature of aging rather than specific to Parkinson's disease. However, because these healthy-control datasets come from different studies and imaging protocols, a quantitative group-wise comparison would not be appropriate.

Regarding the comparison to da Silva Castanheira et al. [29], their findings are based on MEG data, making direct comparisons with our fMRI results also difficult.
Importantly, in the new and updated network-level analyses that we report in the revised manuscript we no longer observe statistically reliable SMT within-network alterations in PD-MH relative to PD-nH. Accordingly, we have therefore removed this interpretation from the revised manuscript to avoid overstatement.

<u>*Major point 10 – Serotonin receptors da Silva Castanheira*</u>

Previous work on brain-fingerprinting in PD also shows that salient brain regions which differentiate individuals with PD from controls negatively colocalize with the distribution of 5-HT1A, 5-HT2A receptors (da Silva Castanheira et al., 2024). I would appreciate it if the authors could contextualize their results with the literature and suggest whether they believe their effects are a reflection of disease load, or are specific to the etiology of minor hallucinations.

We thank Reviewer 1 for this suggestion. The MEG-based fingerprinting study by da Silva Castanheira et al. [29] reports that regions with high individual identifiability in PD negatively colocalize with 5-HT1A

and 5-HT2A receptor density. Although this finding is intriguing, it is challenging to directly relate their MEG results directly to ours for two reasons.

First, the clinical characterization of hallucinations was not reported in detail in that study. As minor or major hallucinations are often not systematically assessed in standard clinical evaluations, it is possible - though purely speculative – that their PD sample included patients with hallucinations. If so, part of the overlap with our findings could indeed reflect disease features related to hallucinations rather than general PD pathology.

Second, direct comparison between MEG- and fMRI-based fingerprinting remains difficult. The two modalities capture distinct physiological processes, and systematic work is still needed to understand which aspects of individual identifiability converge or diverge across modalities.

Given these considerations, we have revised the Discussion to highlight that our results may reflect neural mechanisms specifically associated with minor hallucinations, but we remain cautious in interpreting cross-modal parallels. We now explicitly state that whether the observed effects reflect overall disease load or mechanisms specific to hallucinations cannot be definitively established without harmonized, multimodal datasets.

The revised parts of the Discussion now read as follows:

Finally, our analyses revealed a clear and distinctive neurochemical signature of hallucination-specific brain fingerprints. Functional connections with stronger MH-related brain fingerprint (i.e., positive ΔICC) were located in regions with lower cortical density of serotonin (5-HT2A), GABA and acetylcholine muscarinic M1 receptors, whereas connections with stronger fingerprint in PD-nH (i.e., negative ΔICC) were located in regions with higher densities of these receptors. No significant associations were observed with dopaminergic receptors (D1, D2, DAT), consistent with the fact that ΔICC primarily captures differences between PD subtypes, both of which share dopaminergic alterations. These findings also align with previous work[43], emphasizing the role of non-dopaminergic neurotransmitters in PD-related hallucinations. Previous studies have reported increased 5-HT2A receptor binding in the inferolateral temporal cortex in post-mortem PD patients with hallucinations [44], and in vivo studies have shown higher binding in the visual, dorsolateral, medial-orbitofrontal cortices, and insula in hallucinating PD patients [25]. Similarly, occipital GABAa reduction [26], and muscarinic receptor blockade [45], along with grey matter loss in cholinergic regions [46] have been linked to hallucinations. Notably, these regions do not overlap with MH-related brain-fingerprint areas. A related MEG study compared PD patients to healthy controls and have similarly reported negative co-localization between fingerprinting metrics and serotonin receptors – though no such effects were observed for GABA or M1 [29] – raising questions about the MH-relatedness of our findings. Direct comparison, though, is challenging, as MEG and fMRI capture different physiological processes, and

hallucinations were not characterized in that study, leaving the possibility that some patients experienced minor hallucinations – occurring in up to 40% of PD patients [14,15]. Nevertheless, our observations raise the intriguing possibility that the within-session stability of connectivity captured by brain-fingerprint ICC may reflects compensatory mechanisms in regions relatively spared from hallucinations-related pathology, pointing to a new avenue for mechanistic understanding of hallucinations in PD.

Major point 11 – Brain renders legend

It is very unclear to me what the authors are plotting in Figure 3C. Could the authors elaborate on how they got these plots and what they represent? How did the authors compute 'patient-specific' renders?

Figure 3C shows patient-specific brain renders based on the functional connections that exhibited the strongest hallucination-related fingerprint (top 0.1% ΔICC edges identified at the group level, Fig. 3A). After identifying these edges, we applied the resulting mask to each participant's individual functional connectome and extracted the corresponding FC values. For every subject, we then computed the connectivity strength across these ΔICC-selected edges.

The resulting renders display, for each patient, the strength of these fingerprint-relevant connections, providing a subject-specific visualization of how strongly each individual's FC expresses this network. Although the mask is derived from group-level ΔICC metrics, the FC strength within this set of edges is inherently subject-specific, as previously argued.

The legend of Fig. 3 now reads as follows:

Redacted

Figure 3. Functional connections with high fingerprint associate with clinical scores. A) Brain renders showing the top 0.1% ΔICC edges for each group. The top ΔICC matrices were used as masks to isolate the functional connections that maximise differentiability in fingerprint between the two groups in each individual functional connectome. B) Large scatterplot: linear regression models where clinical scores were significantly associated with functional connectivity in the top ΔICC edges (Clinical score ~ $FC_{\text{top } \Delta ICC \text{ group}}$ + Age + YoE + Disease duration). Small scatterplot: correlation between residuals of the clinical score and functional connectivity to show directionality of the association. C) Patient's specific brain renders showing the functional connections with high fingerprint associated with clinical score. Each render displays, for every participant, the FC strength of the top 0.1% ΔICC edges identified at the group level (Fig. 3A). The group-derived ΔICC mask was applied to each subject's functional connectome, and the FC values within these edges were extracted (and averaged for the score in Fig. 3B). Here, we show the FC intensity of each edge, providing a subject-specific visualization of the expression of the minor hallucination-related fingerprint.

Minor point 1 – medical relevance

In the abstract, the authors report "we show that each patient exhibited a unique brain fingerprint, revealing rich quantifiable, personalized features with medical relevance". I would encourage the authors to elaborate specifically on what they mean by medical relevance so that it is clear to readers how these features are clinically relevant.

We thank the reviewer for this suggestion. By "medical relevance," we refer to the fact that the individualized brain fingerprint features we identify are associated with clinically meaningful measures in Parkinson's disease. Specifically, in our study, we show that the connectivity patterns that maximize fingerprint differentiability between patients with and without hallucinations significantly associates with clinical scores related to motor symptoms severity and cognitive functioning. Thus, these features – completely data-driven – do not merely describe brain connectivity, but reveal connections that are associated with patient-specific symptom profiles, highlighting their direct relevance to clinical assessment and potential personalized interventions. We eliminate this from the abstract to elaborate more in dept in the discussion.

The abstract and discussion now read as follows:

Abstract

Individual variability in connectome organization offers a unique framework for capturing patient-specific alterations. Minor hallucinations (MH) affect up to 40% of Parkinson's disease (PD) patients and are early indicators of cognitive decline, hence crucial for early intervention. Unlike previous group-level studies, connectome-based brain fingerprinting enables to capture individualized neural changes. Applying this approach to PD patients with and without MH using resting-state fMRI, we show that each patient exhibited unique brain fingerprint whose features were associated with clinically relevant measures such as motor symptom severity and cognitive performance. MH-patients showed a loss of subject-specific features in brain networks linked to cognitive health, while cerebellar-to-cortex connectivity – typically less distinctive – became more prominent, emphasizing the role of somatosensory and motor integration in MH pathogenesis. Moreover, MH-related fingerprint regions mapped onto cortical territories with lower densities of neurotransmitter systems implicated in hallucinations. Overall, our fully data-driven findings reveal a distinct, patient-specific connectomic signature that differentiates PD patients with MH.

Discussion

We argue that these findings are particularly noteworthy as they could open new avenues for individualized neuromodulation targets. While the current symptom-specific approach – where stimulation is personalized based on the most burdensome symptoms using group-derived targets – has significantly advanced the field [56], our results suggest a complementary individualized network-based

strategy. Specifically, functional connections with high symptoms-related fingerprint could inform which brain networks to target in a given patient. In practice, this could involve identifying the regions or subnetworks showing the strongest individual fingerprint alterations and using these as candidate sites for neuromodulation (e.g., with real-time neurofeedback, focussed ultrasound, TMS or DBS). If replicated in other cohorts, this approach could pave the way for algorithms that translate individualized network fingerprints into patient-specific stimulation protocols, moving beyond symptoms-based personalisation.

Minor point 2 – multiple comparisons

The authors ran, if I understood correctly, six linear models. It is unclear, however, whether the authors corrected for multiple comparisons and whether they had any specific hypotheses for these analyses or if these tests were entirely exploratory.

Indeed, we ran six linear models to explore the relationship between functional connectivity in high-ICC edges and clinical scores. These analyses were exploratory in nature, as brain fingerprinting is a relatively novel approach and we did not pre-register specific hypotheses for these edgewise connectivity-clinical associations. We acknowledge the importance of correcting for multiple comparisons. For transparency, we note that after FDR correction, some results were marginally significant (e.g., in PD-MH: p = .001, p FDR-corrected = .059). We now report both uncorrected and corrected p-values, but emphasize that these analyses are primarily hypothesis-generating rather than confirmatory.

Results Page 9

[…] For transparency, we report both uncorrected and FDR-corrected p-values. After FDR correction, some results were marginally significant (e.g., PD-nH: p = .003, p FDR-corrected = .031; PD-MH: p = .010, p FDR-corrected = .059). Given the exploratory nature of these analyses, we emphasize that they are primarily hypothesis-generating rather than confirmatory, as fingerprinting is a relatively novel approach and we did not pre-register specific edgewise hypotheses.

Minor point 3 -Typo

There is a typo in Figure 3 caption there is no panel D in the figure.

We thank the reviewer for pointing this out. The reference to panel D in the Figure 3 caption has been corrected.

Minor point 4 – Individualised neuromodulation

I would appreciate it if the authors could elaborate on how they think their results can translate to individualized neuromodulation protocols (e.g., last paragraph of page 11).

We thank Reviewer 1 for this comment and we have expanded the paragraph as follows.

The discussion now reads as follows:

We argue that these findings are particularly noteworthy as they could open new avenues for individualized neuromodulation targets. While the current symptom-specific approach – where stimulation is personalized based on the most burdensome symptoms using group-derived targets – has significantly advanced the field [56], our results suggest a complementary individualized network-based strategy. Specifically, functional connections with high symptom-related fingerprint could inform which brain networks to target in a given patient. In practice, this could involve identifying the regions or subnetworks showing the strongest individual fingerprint alterations and using these as candidate sites for neuromodulation (e.g., with real-time neurofeedback, focussed ultrasound, TMS or DBS). If replicated in other cohorts, this approach could pave the way for algorithms that translate individualized network fingerprints into patient-specific stimulation protocols, moving beyond symptoms-based personalisation.

Minor point 5 – Spatial autocorrelation

I think the authors need to be more specific and explain which spatial autocorrelation-preserving permutation test they performed.

We thank the reviewer for this comment. We have now provided details about the spatial autocorrelation test used. See Methods section at p.23 and response to Major point 5.

Reviewer #2 (Remarks to the Author):

The authors report on functional connectivity patterns generated by resting state fMRI in a group of subjects with relatively early PD with or without minor hallucinations.

We thank the reviewer for the positive assessment of our work and the suggestions. We appreciate the constructive comments and address each point in detail below. All changes in the manuscript are highlighted in blue and underlined.

Major point 1 - Classification MH vs. nH

The subjects and their separation into two groups based on the presence or not of minor hallucinations has been reported in a prior publication and is not elaborated here, although Table 1 summarizes main demographic and clinical features; it is important that the two groups are not different in any one of these main variables.

Although the exact way in which the presence or absence of hallucinations was determined is apparently stated in a prior publication, it would be nice to elaborate on this a little further than what is stated here. Is this separation black and white? Are there levels of severity within the MH group and does this correlate with any of the variables studied here?

We thank Reviewer 2 for the positive evaluation of our matching across groups and for requesting further clarification on group classification. We have now expanded the description in the revised Methods. As detailed, participants were classified as PD-MH if they reported at least weekly, over the past month, episodes of sense of presence, passage hallucinations, or visual illusions, or (i.e. pareidolias.). This resulted in a binary grouping, although we acknowledge that some variability in type, subjective severity, and frequency may exist within the PD-MH group.

Presence and type of minor hallucinations were assessed using the Hallucinations and Psychosis item of the MDS-UPDRS Part I [48] and a semi-structured interview followed to investigating other phenomenological aspects (e.g., frequency and intensity described in words, retention of insight, approximate onset, typical duration, and recency of the last episode). However, the item 1 of the MDS-UPDRS Part I does not collect severity information for minor hallucination specifically and the semi-structured interview was not designed as standardized quantitative assessment.

For these reasons, the present analyses do not include correlational tests between hallucination "severity" and the brain fingerprinting variables. We acknowledge that this would have been of additional interest, and we now highlight this point as a limitation in the manuscript. Our group is very much aware of this issue and in fact has proposed the use of quantitative, experimentally controlled

conditions (e.g., robotic-induced Presence Hallucinations) to capture continuous indices of hallucination susceptibility. Future studies on newly collected datasets within our group will be able to directly relate these quantitative markers to brain connectivity.

The methods and discussion sections now read as follows:

Methods (p.17)

Participants

N=32 patients with PD who previously participated in [20] and N=31 healthy controls (HC) were included in the analyses. All patients fulfilled the MDS new criteria for PD [57] with minor hallucinations (PD-MH, N=18) and without hallucinations (PD-nH, N=14). MH included presence (N=11), passage (N=10) hallucinations, visual illusions or pareidolias (N=6) and/or a combination of two or more types minor hallucinations (N=9). HC were matched for age, sex and years of education, and collected at Newcastle University. Demographics are provided in the 'Statistics and Reproducibility' section). Presence and type of minor hallucinations were assessed using the Hallucinations and Psychosis item of the MDS-UPDRS Part I [48] and a semistructured interview capturing the range of minor hallucinations described in the literature (see Supplementary Material in [20]). Participants reporting at least weekly over the last month episodes of sense of presence, passage hallucinations, visual illusions, or pareidolias in the past month were classified as minor hallucinators. Exclusion criteria included a history of major psychiatric illness, cerebrovascular events, or any medical condition other than PD that could affect cognitive functions. Individuals showing focal structural abnormalities on MRI or presenting with uncontrolled systemic disorders (such as poorly managed diabetes or hypertension) were not eligible. For patients experiencing motor fluctuations, cognitive testing was carried out during their "on" phase. All participants had been maintained on stable dopaminergic treatment for at least four weeks prior to enrolment, and only patients whose hallucination profile had remained unchanged for the preceding three months were included. None had a history of antipsychotic medication use. Vision was normal or corrected to normal in all participants.

Discussion (p.16)

Finally, while our results provide valuable insights into the features of brain-fingerprint in PD patients with minor hallucinations, it would be particularly interesting to examine how these features evolve over time, whether they relate with variability of hallucinatory phenomena, if they can predict later clinical cognitive impairments, and whether the observed fingerprinting patterns change as new symptoms appear.

Major point 2 – early vs. advanced stages of PD.

The authors convincingly show the ability to distinguish unique individual patterns of brain connectivity in each one of the subjects of the study, regardless of the group they belong to. This is an important finding.
The authors state: "Our results demonstrate that individual functional connectivity patterns contain stable, subject-specific features, enabling patient identification in PD". Perhaps here they should specify that the evidence concerns early PD, as such individual patterns may be lost in advanced disease, which is not studied here.

We thank Reviewer 2 for their positive feedback. We agree that the individual patterns we describe are observed in patients not in advanced stages of PD, and it remains to be investigated whether such stable, subject-specific connectivity features persist in later disease stages. We have added this in the discussion.

The Discussion section (p. 12) now reads as follows:

Our results demonstrate that individual functional connectivity patterns contain stable, subject-specific features, enabling patient identification in PD. These findings extend previous evidence from MEG studies in PD [28,29] and align with fMRI studies in other neurodegenerative disorders [27]. The persistence of brain fingerprinting in neurodegenerative conditions is a non-trivial finding because it shows that even within a homogeneous group – patients with the same diagnosis, same range of biological alterations and symptoms, and, in this case, belonging to the same subtype with potentially similar clinical trajectories – each individual remains highly distinct. Features embedded in these functional connectomes hold, therefore, great promise for advancing personalized prognostics and treatments by providing objective, data-driven biomarkers to identify individual differences, track disease progression, and may predict variability in therapeutic responses [58,59]. We note, however, that our cohort included patients with a relatively mild-to-moderate disease duration (mean ± SD: 5.2 ± 3.8 and 4 ± 2 years for the two groups). Future studies will be needed to determine whether these stable, subject-specific connectivity patterns persist in more advanced stages of PD.

Major point 3 – Difference in ICC with HC
3- They also show, in Figure 2, significant differences in functional connectivity patterns in specific brain regions between the two groups; it is very interesting that the high identifiability in the nH group is similar to reported healthy controls, while the individuals in the MH group are divergent. It would have been preferable to have controls within the present study to further substantiate this point.

We thank Reviewer 2 for raising this important point. While fingerprinting in healthy populations - including healthy older adults (e.g., [27] - has been extensively characterised in previous work, we agree that comparisons with HC can offer additional perspective on the present findings. Unfortunately, we do not have access to an HC dataset acquired at the same site and with the same MRI protocol as our PD cohort, and collecting new site-matched HC data is unfeasible. Although external HC datasets exist, direct quantitative comparisons are not recommended because fingerprinting measures (including ICC) are highly sensitive to scanner-specific factors and signal-to-noise ratio [39].

Nevertheless, to provide an additional point of reference and following a similar request by Reviewer 1 (see Major point 4), we have included an external HC dataset and confirmed that the connections differentiating PD-MH from PD-nH only minimally overlapped with those differentiating PD from HC (cf. new Supplementary Figure 1). This confirms that our results primarily reflect MH-related features. Consistently, as shown in the new Supplementary Figure 1, the high identifiability observed in the DMN, FPN, and SAL in the PD-nH group qualitatively resembles patterns reported in healthy controls, whereas the high identifiability in the FPN and cerebellum in PD-MH individuals diverges from healthy controls, supporting our interpretation even in the absence of a direct site-matched HC comparison.

The revised parts of the Methods, Results, and Supplementary Materials sections now reads as follows:
Methods (p.21):

Minor Hallucination-related differential ICC

In these analyses, we aimed to identify edges that were MH-related, meaning those that contributed to differences in fingerprint between the PD patients with and without MH. To achieve this, we first computed a ΔICC matrix to capture MH-related differential identifiability, defined as:

$$\Delta\, ICC_{PD-MH-PD-nH} = ICC\ mat_{PD-MH} - ICC\ mat_{PD-nH}$$

As a result, the $\Delta ICC_{PD\text{-}MH - PD\text{-}nH}$ matrix (Fig. 2B) contained positive values for functional connections with higher ICC in PD-MH and negative values for those with higher ICC in PD-nH. Values close to zero indicated similar ICC across both groups, suggesting individual variability that did not contribute to distinguishing features between them. We identified group-specific ICC edges by selecting the positive or negative delta-ICC edges, for PD-MH and PD-nH respectively. To determine whether the features most strongly distinguishing PD-MH from PD-nH were MH-related rather than more generally associated with PD, we performed the following analysis. First, we computed a second, PD-specific, differential ICC map defined as:

$$\Delta\, ICC_{PD-HC} = \frac{ICC\ mat_{PD-MH} + ICC\ mat_{PD-nH}}{2} - ICC\ mat_{HC}$$

Next, from this $\Delta ICC_{PD–HC}$ map, we retained only edges whose absolute values exceeded the minimum absolute value within the top 10% of $\Delta ICC_{PD\text{-}MH – PD\text{-}nH}$ (shown in Fig.S1B), and whose sign matched the direction of $\Delta ICC_{PD\text{-}MH – PD\text{-}nH}$. This was done to test the extent to which PD–HC ICC differences followed the same directionality of the strongest PD-MH vs PD-nH ICC differences, thereby assessing whether edges most strongly associated with MH overlapped with those reflecting PD-specific alterations. Finally, we quantified the overlap (Fig. S1C).

Results (p.6):

Spatial specificity of brain fingerprint of minor hallucinations in PD

Analysing the local properties of the brain fingerprint using edgewise intra-class correlation (ICC, Amico & Goñi, 2018a), we observed that the functional connections with the highest fingerprint exhibited different spatial distributions in PD-nH, PD-MH and HC (Fig. 2A and Fig. S1). To identify ICC edges that differed between the PD patients with and without MH – i.e., features related to MH rather than general PD characteristics - we computed an edge-based differential identifiability (ΔICC) matrix (i.e., $ICC_{PD\text{-}MH} – ICC_{PD\text{-}nH}$; Fig. 2B, see 'MH-related differential ICC'). To test whether the features that most strongly differentiated PD-MH from PD-nH represented a signature of MH, rather than features generally associated with PD, we compared them with an edgewise differential identifiability matrix contrasting PD patients (PD-MH + PD-nH) with healthy controls (i.e., $ICC_{PD} – ICC_{HC}$). We found that only 0.5% of the top $\Delta ICC_{PD\text{-}MH – PD\text{-}nH}$ connections showed the same direction of effect and exceeded the minimal ΔICC value observed in PD-MH vs PD-nH (ICC = 0.5), suggesting that the edges with the highest $\Delta ICC_{PD\text{-}MH – PD\text{-}nH}$ values are largely related to MH (see Fig. S1).

Supplementary Materials (p.6):

Minor Hallucination related differential identifiability

Redacted

Figure S1. Overlap between brain fingerprint features related to hallucinations and those differentiating Parkinson's disease patients from healthy controls. A) Edgewise intra-class correlations (ICC) matrices for Parkinson's Disease patients without hallucinations (PD-nH) and with minor hallucinations (PD-MH) and healthy controls (HC). ICC quantifies within-subjects similarity between test and retest for each edge (FC between 2 regions). ICC matrices for PD-nH and PD-MH are the same as those displayed in Figure 2A of the main text; they are reported here to allow visual comparison. We display edges with ICC between the 50th and the 95th percentile, across the 7 resting-state networks (RSN), subcortical and cerebellar regions. B) ΔICC matrix showing the difference in ICC between PD-MH and PD-nH, as in Figure 2B, here thresholded for the top 10% absolute values. C) ΔICC matrix showing the difference in ICC between PD patients (PD-MH and PD-nH combined) and HC, restricted to edges showing the same direction of effect and exceeding the minimal ΔICC value observed in the PD-MH vs PD-nH comparison (ΔICC = 0.5, where ICC is considered 'good' above 0.6[40]). This corresponded to 0.5% of the total number of top edges shown in B). A), B) and C) are ordered according the 7 RSNs, subcortical and cerebellar regions. VIS=Visual Network; SMT=Somatomotor Network; DA=Dorsal Attention Network; SA=Salience Network; L=Limbic Network; FPN=Fronto-Parietal Network; DMN=Default-Mode Network; SBC=Subcortical regions; CRB=Cerebellum.

Discussion (p. 13)

In healthy subjects, the most prominent networks for subject identification are the DMN, FPN and SAL [21,22], a pattern that has also been confirmed in cognitively healthy older adults in previous work [27] and found also in the present HC dataset (see Fig. S1). Here, PD-MH, relative to PD-nH, exhibited lower ΔICC in DMN-within-network connections, as well as in DMN-FPN, DMN-SAL, DMN-VIS and SAL-

FPN edges (Fig. 2D), indicating diminished subject-specific features in these networks. This pattern aligns with the notion that PD-MH have a more severe and rapidly advancing form of PD often associated with cognitive impairments [12] and may reflect early network fragility or emerging impairment. PD-nH, who are less likely to experience cognitive decline [23], maintain a more stable, cognitively-healthy brain fingerprint anchored in these core systems. Relative to PD-nH, PD-MH patients also showed lower ΔICC also in the subcortical regions, and their connection with DMN and FPN. Although speculative, these fingerprinting results are compatible with observations from both resting-state and task-based studies and the attentional network hypothesis. This hypothesis posits that hallucinations are associated with overactivation of the DMN and SAL networks, whose interaction may lead to failures in stimulus-driven control and a bias toward internally generated perceptions 27. Such overactivation could be reflected in reduced reliability of these networks.

By contrast, PD-MH patients exhibited a stronger ΔICC in FPN within-network connections, as well as FPN-DAN and FPN-cerebellum connections. Qualitatively, FPN ICC values also appeared higher in PD-MH than in HC (Fig. S1). Even though it is difficult to reconcile fingerprint results with findings from activation studies, our results are nonetheless compatible with evidence of increased frontal theta activity in patients with MH, associated with lower cognitive functioning in an earlier EEG study[14], and potentially reflecting early compensatory mechanisms.

We also observed increased ΔICC in within-cerebellar pairs, as well as in its connectivity with DAN, SMT and VIS network. Qualitatively, cerebellar ICC also appeared higher in PD-MH than in HC (Fig. S1). Cerebellar lesions have been associated with hallucinations in a cohort of >~80 patients [52], and reduction in grey matter volume in the cerebellum has been implicated in MH in a previous study [53]. As argued in recent work [54], there is a significant number of findings supporting the role of the cerebellum in psychosis, and on its role in the construction and adaption of internal models integrating sensorimotor inputs and cognitive control. This is in line with previous accounts for MH highlighting the importance of the somatosensory and motor cues and the integration of these signals in MH, including presence hallucinations, which have recently been linked to specific somatomotor processes in PD patients [16] and in healthy subjects in whom presence hallucinations have been induced experimentally using a somatomotor conflicts (based on tactile, motor, and proprioceptive signals) [55], associated with activation of primary motor and premotor cortex (cf. Figure 2E in [16]). We note that only 0.5% of the top ΔICC PD-MH vs PD-nH edges overlapped with those differentiating PD from HC, confirming that these effects primarily reflect MH-specific features (see Fig. S1).

Major point 4 - Interpretation of network results

The authors state: "In the present study, PD patients without hallucinations –who are less likely to experience cognitive decline maintain high identifiability relying upon the DMN, FPN and SAL (Fig. 2A)." FPN identifiability is substantially lower than in the MH group; this is discussed, but perhaps the above statement could be tempered. In any case, it is also very interesting that the DMN and SAL networks, differentially involved in the MH group, have also been implicated in more advanced cases with complex visual hallucinations.

The deficiencies in frontal-executive networks observed here in the MH group have been reported before, but the entirely data-driven approach here provides robust confirmation of earlier findings in a most authoritative fashion.

We thank the reviewer for recognizing the value of our data-driven approach in extending and confirming previous findings. We have tempered our interpretation of alterations in frontal executive regions as seen below, and further expanded the discussion by linking fingerprint differences in the DMN and SAL networks to the existing literature.

The Discussion section (p. 15) now reads as follows:

In healthy subjects, the most prominent networks for subject identification are the DMN, FPN and SAL [21,22], a pattern that has also been confirmed in cognitively healthy older adults in previous work[27] and found also in the present HC dataset (see Fig. S1). Here, PD-MH, relative to PD-nH, exhibited lower ΔICC in DMN-within-network connections, as well as in DMN-FPN, DMN-SAL, DMN-VIS and SAL-FPN edges (Fig. 2D), indicating diminished subject-specific features in these networks. This pattern aligns with the notion that PD-MH have a more severe and rapidly advancing form of PD often associated with cognitive impairments [12] and may reflect early network fragility or emerging impairment. PD-nH, who are less likely to experience cognitive decline [23], maintain a more stable, cognitively-healthy brain fingerprint anchored in these core systems. Relative to PD-nH, PD-MH patients also showed lower ΔICC also in subcortical regions, and their connection with DMN and FPN. Although speculative, these fingerprinting results are compatible with observations from both resting-state and task-based studies and the attentional network hypothesis. This hypothesis posits that hallucinations are associated with overactivation of the DMN and SAL networks, whose interaction may lead to failures in stimulus-driven control and a bias toward internally generated perceptions [8]. Such overactivation could be reflected in reduced reliability of these networks. By contrast, PD-MH patients exhibited a stronger ΔICC in FPN within-network connections, as well as FPN-DAN and FPN-cerebellum connections. Qualitatively, FPN ICC values also appeared higher in PD-MH than in HC (Fig. S1). Even though it is difficult to reconcile fingerprint results with findings from activation studies, our results are compatible with evidence of increased frontal theta activity in patients with MH,

associated with lower cognitive functioning in an earlier EEG study [14], and potentially reflecting early compensatory mechanisms.

Major point 5 – Improve interpretation of neurotransmitter

The findings with the correlation of apparent decreased receptor binding in areas of high fingerprint connectivity in the MH group are for the most part highly significant, surviving Bonferroni correction for multiple comparisons, and remarkably in the opposite direction in almost all cases in the nH group. It is quite impressive how the two groups move in opposite directions in this regard, but the interpretation of this obvious effect is not very clearly stated in the discussion.

Thank you. We have clarified the interpretation of these results in the Discussion, highlighting that areas with decreased receptor binding in regions of high fingerprint connectivity in the PD-MH group show the opposite pattern in PD-nH patients, emphasizing the contrasting neurochemical signatures of the two subgroups.

The Discussion section (p. 15) now reads as follows:
Finally, our analyses revealed a clear and distinctive neurochemical signature of MH-related brain fingerprints. Functional connections with stronger MH-related brain fingerprint (i.e., positive ΔICC) were located in regions with lower cortical density of serotonin (5-HT2A), GABA and acetylcholine muscarinic M1 receptors, whereas connections with stronger fingerprint in PD-nH (i.e., negative ΔICC) were located in regions with higher densities of these receptors. No significant associations were observed with dopaminergic receptors (D1, D2, DAT), consistent with the fact that ΔICC primarily captures differences between PD subtypes, both of which share dopaminergic alterations. These findings also align with previous work[43], emphasizing the role of non-dopaminergic neurotransmitters in PD-related hallucinations. Previous studies have reported increased 5-HT2A receptor binding in the inferolateral temporal cortex in post-mortem PD patients with hallucinations [44], and in vivo studies have shown higher binding in the visual, dorsolateral, medial-orbitofrontal cortices, and insula in hallucinating PD patients [25]. Similarly, occipital GABAa reduction [26], and muscarinic receptor blockade [45], along with grey matter loss in cholinergic regions [46] have been linked to hallucinations. Notably, these regions do not overlap with MH-related brain-fingerprint areas. Related MEG studied comparing PD patients to healthy controls have similarly reported negative co-localization between fingerprinting metrics and serotonin receptors – though no such effects were observed for GABA or M1 [29] – raising questions about the MH-relatedness of our findings. Direct comparison, though, is challenging, as MEG and fMRI capture different physiological processes, and hallucinations were not characterized in that study, leaving the possibility that some patients experienced minor hallucinations – occurring in up to

40% of PD patients [14,15]. For these reasons, it is not possible to fully reconcile the two studies. Nevertheless, our observations raise the intriguing possibility that the within-session stability of connectivity captured by brain-fingerprint ICC may reflect compensatory mechanisms in regions relatively spared from hallucinations-related pathology, pointing to a new avenue for mechanistic understanding of hallucinations in PD.

Minor points

The limitation section is appreciated.
We thank Reviewer 2 for the positive comment and appreciation of the limitations section.

Ref 10 is repeated as ref 30.
We thank Reviewer 2 for spotting this and bringing it to our attention. The bibliography has now been corrected.

Overall, this is an extremely well substantiated and presented piece of work on an important topic, that of minor hallucinations in relatively early stages of PD, an intriguing feature of the disease. Methodology used is state of the art. The manuscript is written in a very organized fashion, facilitating readership despite its highly complex nature. It represents a significant addition to the field.

We thank the reviewer for their positive and encouraging comments. We are glad that the significance, methodology, and clarity of our work are appreciated, and we hope it will indeed contribute meaningfully to the field.

Reviewer #3 (Remarks to the Author):

In this manuscript, the authors studied minor hallucinations in PD patients using connectome-based brain fingerprints technique and associated it with cognitive decline in PD patients. A total of 32 PD patients is included, with 18 of them have minor hallucinations and 14 of them having no hallucinations. This study is a re-analysis of a previously published study (Bejr-kasem et al., Mov. Disord. 2019). There are a few concerns about this study though.

We thank Reviewer 3 for assessing our work. We appreciate the opportunity to respond to the Reviewer's concerns and have addressed each point in detail below. All changes in the manuscript are highlighted in blue and underlined.

Major point 1 – Small sample size

1. The sample size is small for fingerprints analysis.

We agree that the sample size is relatively small for neuroimaging analysis in general. However, importantly, our PD cohort is highly unique: all patients underwent resting-state fMRI and were evaluated in detail using a high-quality clinical and hallucination assessment; they were also cognitively well-characterized and were without even mild cognitive impairment, and showed no differences in medication or other key clinical variables between groups. The present data therefore combine a highly clinically characterized Parkinson's disease cohort with resting-state fMRI data, which has now been complemented by an additional analysis of MRI data from a new healthy control group. Overall, this combination is extremely rare in PD cohorts with minor hallucinations.

In addition, the fact that with ICC-selected edges we can reveal individual patterns that relate with clinical variability, confirms that our analyses are capturing biologically meaningful features. ICC values can be influenced by sample size differences, however, and to address this, we performed permutation analyses, re-running the fingerprinting analyses with randomly subsampled groups of the same size 1,000 times, which supported the robustness of our findings despite the limited sample. Based on the comment by Reviewer 3, we now explicitly acknowledge that future studies with larger, and/or more densely-sampled specifically optimized cohorts for fingerprinting are warranted in studies of non-motor symptoms in PD to further validate and extend the present findings.

The Discussion section (p. 16) now reads as follows:

The present study has some limitations. First, although the sample size is relatively small, this study offers a rare opportunity to investigate a cohort stratified by minor hallucinations. Such databases are scarce, making this work a valuable step toward understanding brain-fingerprint in this specific group

of patients and early in the disease course. Future studies including larger and/or more densely sampled cohorts are warranted to further validate these findings. [..]

Major point 2 – Scan length

The scan length also is barely enough for robust functional connectome analysis and fingerprints validation.

We thank the reviewer for raising this important point regarding scan duration. As noted in the original Methods (p.19), recent work in connectome identifiability has demonstrated that a good level of fingerprinting accuracy across resting-state networks can already be achieved with approximately 200 seconds of data (see Fig. 4B in reference 75). In our study, both the test and retest segments contained 90 volumes with a TR of 2 seconds (180 seconds each), which falls within the range shown to yield reliable individual identifiability across the whole brain. We do, however, fully agree that longer acquisitions – such as those used in precision fMRI frameworks [60] – may further improve the reliability of functional connectivity estimates. However, long-duration resting-state protocols currently remain difficult to obtain in Parkinson's disease clinical cohorts. For this reason, we believe that the present dataset reflects an acceptable trade-off between feasibility and data quality in a clinical population.

We now explicitly acknowledge this limitation in the revised manuscript and highlight that future work should examine how brain-fingerprint features evolve with longer acquisitions, as well as assess whether extended scanning improves both identifiability and clinical predictiveness.

The Discussion section (p. 16) now reads as follows:

The present study has some limitations. First, although the sample size is relatively small, this study offers a rare opportunity to investigate a cohort stratified by minor hallucinations. Such databases are scarce, making this work a valuable step toward understanding brain-fingerprint in this specific group of patients and early in the disease course. Second, this study divided the same scanning session into two in order to estimate brain identifiability, addressing challenges in obtaining two separate test-retest sessions in clinical cohorts as in [27]. While this approach been shown to yield similar results to between-session data [22], future research will need to replicate our findings. Third, although ~200 seconds of resting-state data are sufficient for good identifiability, longer acquisitions – as used in precision fMRI [60] – typically yield more robust connectivity estimates; future studies should therefore examine how fingerprint features change with extended scan durations. Finally, while our results provide valuable insights into the features of brain-fingerprint in PD patients with minor hallucinations, it would be particularly interesting to examine how these features evolve over time, if they can predict later clinical

cognitive impairments, and whether the observed fingerprinting patterns change as new symptoms appear.

Major point 3 - Results are descriptive

No obvious group differences are detected by the major fingerprints-based indices. Most of results are descriptive.

We respectfully disagree and clarify that the manuscript includes formal statistical analyses at both whole-brain and spatially specific levels.

Specifically, for the whole-brain indices (ISelf, IOthers), we conducted ANOVAs comparing PD-MH, PD-nH, and now also healthy controls (HC). These analyses did not reveal significant group differences, confirming that identifiability is maintained in the presence of PD, and irrespective of minor hallucination status.

In addition, we examined group differences at the spatial level by analysing the distribution of connections with the highest fingerprint differences between PD-MH and PD-nH, using edgewise differential ICC (ΔICC). Group differences for high ΔICC across functional networks were tested using a binomial test with FDR correction. Also, in response to Reviewer 1 (see Major point 7), we now also reported results using a different threshold for high ΔICC and have added these new results in the Supplementary Materials (cf. new Supplementary Figure 2), strengthening the robustness of our results. This approach enabled us to identify specific connections showing statistically significant MH-related differences.

Finally, we also added another new analysis in response to Reviewer 1 (see Major point 3) to further explore whether ΔICC-selected edges could support group discrimination. For this, we trained a leave-one-out SVM classifier using edges exceeding percentile thresholds of ΔICC. Maximal classification accuracy was identified, permutation-tested, and compared with both random and t-test–based feature selection (Fig. 3E–G).

These results demonstrate that, although major fingerprint indices did not differ, ΔICC-based features provide statistically robust between-group discriminability. We hope this clarifies that our findings go beyond descriptive observations.

Below, we now report all group-level analyses performed, highlighting in blue those added in the current revision.

Please see below the part of the manuscript reporting group-level analyses:

As we note in the Methods section (p. 25):

"*ISelf* and *IOthers* were normally distributed in each group. Analyses were performed using MATLAB and RStudio. To compare *ISelf* vs. *IOthers* in each group we performed paired-sample t-test (ttest, MATLAB 2022a). Then, we used one-way ANOVAs to test the effect of group on *ISelf* and *IOthers* separately after checking for nuisance variables, with 5000 permutations to control for sample size differences (using aovperm from permuco R-package)[61] (Supplementary Table 1). For *ISelf*, the nuisance variables were age, sex, years of education (YoE), site of acquisition and the difference in motion between the test and retest scans, as absolute difference between FD (*ISelf* ~ Group + Age + Sex + YoE + Site + ΔFD). For *IOthers*, the nuisance variables were also age, sex, and YoE, site of acquisition but motion (FD) was considered across the entire acquisition, as *IOthers* is a composite measure across test and retest *(IOthers ~ Group + Age + Sex + YoE + FD)."*

As we note in the Methods section (p. 22):

"Finally, we examined the distribution of the hallucination-specific ICC edges across functional networks. Specifically, we computed the percentage of edges per network exceeding ΔICC = ±~0.5 (corresponding to 97th percentile and considered to reflect "fair" ICC[40]) separately for PD-MH and PD-nH and tested for group differences using a binomial test (function binocdf in Matlab), applying FDR correction for multiple comparisons (Fig. 2D). Results for analyses without thresholding, as well as for higher thresholds (98th and 99th percentiles), are reported in the Supplementary Materials."

Methods section (p.22)

Between-groups discriminability using ΔICC-selected FC-edges

In the above analyses, we identified the spatial features of brain-fingerprints that showed maximal differences between the two groups (ΔICC). However, it remained unclear whether these features also allowed between-group discriminability, that is, whether individuals with MH could be distinguished from those without (nH) based on FC features derived from ΔICC. To test this, we trained a support vector machine (SVM) classifier with leave-one-out cross-validation (LOOCV), using percentile thresholds ranging from the 1st to the 99.9th (in 1-percentile steps) of the absolute ΔICC distribution to classify participants as PD-MH versus PD-nH. This approach allowed us to identify the percentile threshold – and thus the number of FC edges – that yielded maximal between-group classification accuracy above chance level (chance level = proportion of participants in the larger group, i.e., MH = 18/32 = 56.25%, Fig. 3E).

Using the optimal number of FC features (n = 380) yielding maximal discriminability, we performed 1,000 permutation tests by randomly shuffling group labels to assess whether the observed accuracy exceeded chance-level classification (Fig. 3E). In addition, we compared this accuracy with that obtained when randomly selecting the same number of FC edges (n = 380) across the connectome (Fig.

3F), and with that obtained when selecting edges based on group-level statistical differences (two-sample t-tests between PD-MH and PD-nH). This latter analysis allowed us to compare the ΔICC-based feature selection with a classical group-difference–based feature selection approach, using the same classifier architecture and cross-validation scheme. Finally, to verify that random accuracy was not influenced by the number of selected edges, we progressively increased the number of FC features (from the 1st to the 99.9th percentile, in 1-percentile steps) and computed between-group classification accuracy at each step (Fig. 3G). All classifiers were implemented in MATLAB using the *fitclinear* function (Learner = 'svm'; Solver = 'lbfgs'; Regularization = 'ridge').

Results section (p.26)

Between-groups classification accuracy using FC-edges with highest brain-fingerprint

To assess between-group discriminability, we tested whether the FC features showing the largest ICC differences also supported classification between PD-MH and PD-nH. Using an SVM model with leave-one-out cross-validation, classification accuracy peaked at 71.9% when using the top 380 FC edges (99th percentile of the absolute ΔICC distribution), significantly above chance based on 1,000 label-shuffling permutations ($p = 0.005$; Fig. 2E). This accuracy was also higher than that obtained with randomly selected 380 FC edges (mean = 49.9%, $p = 0.006$; Fig. 2F), and all combination of randomly chosen features were below chance level when their number was varied up to the whole-brain level (~37'000 edges; Fig. 2G). Classification accuracy obtained by selecting the most different (99th percentile of the absolute t-values distribution) edges based on group-level statistical differences (two-sample t-tests between PD-MH and PD-nH) was however higher (accuracy = 100%). These results indicate that top ΔICC connections meaningfully contribute to between-group classification, above what expected by chance and/or random models. However, classification accuracy using ΔICC-selected edges was not higher than that obtained using edges selected based on group differences via t-tests, suggesting that while ICC highlights reproducible features, t-test-selected edges perform better for between-group classification.

Major point 4 – Interpretation of spatial features findings

Spatial specific measures detected some changes and clinical association in both PD-MH and PD-nH groups. It is hard to say what really are the real pathological changes leading to mental hallucinations in PD.

Our analyses show changes in connections with highest fingerprint in PD-MH and PD-nH, and that the individual-specific functional-connections with these maximal differences are associated with different clinical measures. Namely, in PD-MH patients, edges most strongly related to MH are associated with cognitive functioning, consistent with the higher risk of cognitive decline in these patients. In PD-nH patients, connections showing the highest differential fingerprinting are linked to non-cognitive

functions, such as motor performance. Furthermore, regions with high MH-related fingerprint correspond to areas with lower density of neurotransmitter receptors implicated in hallucinations, suggesting a potential compensatory role of these regions. Overall, MH-related fingerprinting highlights network and regional patterns plausibly linked to the pathophysiology of MH. However, it should be noted that identifying pathological changes related to MH using fingerprinting is currently less established than using activation studies or standard resting-state approaches, therefore interpretation should be cautious. We acknowledge that the relationship between prior literature on the neural basis of MH – including both resting-state connectivity and task-based findings - and fingerprinting is complex. Accordingly, in the revised manuscript, we provide a clearer interpretation of how our results relate to previous studies in the Discussion.

Please see below part of the discussion where we provide a clearer interpretation of our results:

[…]

In healthy subjects, the most prominent networks for subject identification are the DMN, FPN and SAL [21,22], a pattern that has also been confirmed in cognitively healthy older adults in previous work[27] and found also in the present HC dataset (see Fig. S1). Here, PD-MH, relative to PD-nH, exhibited lower ΔICC in DMN-within-network connections, as well as in DMN-FPN, DMN-SAL, DMN-VIS and SAL-FPN edges (Fig. 2D), indicating diminished subject-specific features in these networks. This pattern aligns with the notion that PD-MH have a more severe and rapidly advancing form of PD often associated with cognitive impairments [12] and may reflect early network fragility or emerging impairment. PD-nH, who are less likely to experience cognitive decline [23], maintain a more stable, cognitively-healthy brain fingerprint anchored in these core systems. Relative to PD-nH, PD-MH patients also showed lower ΔICC also in subcortical regions, and their connection with DMN and FPN. Although speculative, these fingerprinting results are compatible with observations from both resting-state and task-based studies and the attentional network hypothesis. This hypothesis posits that hallucinations are associated with overactivation of the DMN and SAL networks, whose interaction may lead to failures in stimulus-driven control and a bias toward internally generated perceptions [8]. Such overactivation could be reflected in reduced reliability of these networks. By contrast, PD-MH patients exhibited a stronger ΔICC in FPN within-network connections, as well as FPN-DAN and FPN-cerebellum connections. Qualitatively, FPN ICC values also appeared higher in PD-MH than in HC (Fig. S1). Even though it is difficult to reconcile fingerprint results with findings from activation studies, our results are compatible with evidence of increased frontal theta activity in patients with MH, associated with lower cognitive functioning in an earlier EEG study [14], and potentially reflecting early compensatory mechanisms.

We also observed increased ΔICC in within-cerebellar pairs, as well as in its connectivity with DAN, SMT and VIS network. Qualitatively, cerebellar ICC also appeared higher in PD-MH than in HC (Fig. S1). Cerebellar lesions have been associated with hallucinations in a cohort of >~80 patients [52], and reduction in grey matter volume in the cerebellum has been implicated in MH in a previous study [53]. As argued in recent work [54], there is a significant number of findings supporting the role of the cerebellum in psychosis, and on its role in the construction and adaption of internal models integrating sensorimotor inputs and cognitive control. This is in line with previous accounts for MH highlighting the importance of somatosensory and motor cues and the integration of these signals in MH, including presence hallucinations, which have recently been linked to specific somatomotor processes in PD patients [16] and in healthy subjects, in whom PH have been induced experimentally using a somatomotor conflicts (based on tactile, motor, and proprioceptive signals) [55], associated with activation of primary motor and premotor cortex (cf. Figure 2E in [16]).

[…]

Finally, our analyses revealed a clear and distinctive neurochemical signature of MH-related brain fingerprints. Functional connections with stronger MH-related brain fingerprint (i.e., positive ΔICC) were located in regions with lower cortical density of serotonin (5-HT2A), GABA and acetylcholine muscarinic M1 receptors, whereas connections with stronger fingerprint in PD-nH (i.e., negative ΔICC) were located in regions with higher densities of these receptors. No significant associations were observed with dopaminergic receptors (D1, D2, DAT), consistent with the fact that ΔICC primarily captures differences between PD subtypes, both of which share dopaminergic alterations. These findings also align with previous work on complex visual hallucinations [43], emphasizing the role of non-dopaminergic neurotransmitters in PD-related hallucinations. Previous studies have reported increased 5-HT2A receptor binding in the inferolateral temporal cortex in post-mortem PD patients with hallucinations [44], and in vivo studies have shown higher binding in the visual, dorsolateral, medial-orbitofrontal cortices, and insula in hallucinating PD patients [25]. Similarly, occipital GABAa reduction [26], and muscarinic receptor blockade [45], along with grey matter loss in cholinergic regions [46] have been linked to hallucinations. Notably, these regions do not overlap with the MH-related brain-fingerprint areas. A related MEG study compared PD patients to healthy controls and have similarly reported negative co-localization between fingerprinting metrics and serotonin receptors – though no such effects were observed for GABA or M1 [29] – raising questions about the MH-relatedness of our findings. Direct comparison, though, is challenging, as MEG and fMRI capture different physiological processes, and hallucinations were not characterized in that study, leaving the possibility that some patients experienced minor hallucinations – occurring in up to 40% of PD patients [14,15].

Nevertheless, our observations raise the intriguing possibility that the within-session stability of connectivity captured by brain-fingerprint ICC may reflect compensatory mechanisms in regions relatively spared from hallucinations-related pathology, pointing to a new avenue for mechanistic understanding of hallucinations in PD.

Major point 5 – Reproducibility of pre-processing pipeline

The in-house fMRI processing pipeline might reduce the reproducibility of this study.

We note that the used preprocessing is not a closed or idiosyncratic in-house tool: it is fully openly available on GitHub and has been extensively documented. The pipeline was originally developed in one of the leading connectomics laboratories (Indiana University, in collaboration with Olaf Sporns) and has been widely used in prior work [22,27,38]. Importantly, no standard package (e.g., fMRIPrep) currently performs the complete set of processing steps in native space required for fingerprinting analyses. For this reason, pipelines addressing these specific requirements bring along some degree of customization. In any case, the transparency, public availability, and prior validation of our pipeline ensure full reproducibility of the analyses presented here.

Major point 6 – Hallucinations Assessment

Mental hallucination is defined by using one study visit based on patient recall. There is no consideration about the accuracy of mental hallucination diagnosis.

We thank Reviewer 3 for this comment and acknowledge that the assessment procedure for minor hallucinations may not have been sufficiently clear in the previous version of the manuscript. We have now added further methodological details in the Methods section to clarify how minor hallucinations were evaluated. Importantly, hallucination classification did not rely on a single-visit subjective report. Clinical professionals used the standardized MDS-UPDRS hallucinations item together with a semi-structured interview. We do agree that hallucination evaluations remain difficult to quantify, as compared to other clinical signs, such as tremor. Our group is very much aware of this issue and in fact has proposed the use of quantitative, experimentally controlled conditions to capture continuous indices of hallucination susceptibility. This includes studies that experimentally induce specific clinically relevant hallucinations, like Presence Hallucinations in PD, using robotic devices and virtual reality [16,62,63] and to compare these quantified induced hallucinations with disease-related hallucinations that patients report in their daily life. Future studies on newly collected datasets within our group will be able to directly relate these quantitative markers to brain connectivity. We have added to the revised discussion that future fingerprinting studies in PD with hallucinations should also include behavioural data using hallucination-inducing paradigms and patient sensitivity to such procedures.

The Methods and Limitation sections now reads as follows:

Methods (p.17)

Participants

N=32 patients with PD who previously participated in [20] and N=31 healthy controls (HC) were included in the analyses. All patients fulfilled the MDS new criteria for PD [57] with minor hallucinations (PD-MH, N=18) and without hallucinations (PD-nH, N=14). MH included presence (N=11), passage (N=10) hallucinations, visual illusions or pareidolias (N=6) and/or a combination of two or more types minor hallucinations (N=9). HC were matched for age, sex and years of education, and collected at Newcastle University. Demographics are provided in the 'Statistics and Reproducibility' section). Presence and type of minor hallucinations were assessed by clinical professionals using the Hallucinations and Psychosis item of the MDS-UPDRS Part I [48] and a semi-structured interview capturing the range of minor hallucinations described in the literature (see Supplementary Material in [20]). Participants reporting at least weekly over the last month episodes of sense of presence, passage hallucinations, visual illusions, or pareidolias in the past month were classified as minor hallucinators. […]

Limitation (p. 16)

[…]

Second, we agree that hallucinations are difficult to quantify, unlike other clinical signs such as tremor. Our group has addressed this by studying hallucinations in PD using behavioural paradigms, including experiments inducing presence hallucinations with robotic and VR devices [16,62,63], and comparing these induced experiences with patients' real-life hallucinatory symptoms. Future neuroimaging studies should integrate such behavioural hallucination data and account for individual patient sensitivity.

References:

1. Diederich, N. J., Fénelon, G., Stebbins, G. & Goetz, C. G. Hallucinations in Parkinson disease. *Nat Rev Neurol* **5**, 331–342 (2009).
2. Lenka, A., Jhunjhunwala, K. R., Saini, J. & Pal, P. K. Structural and functional neuroimaging in patients with Parkinson's disease and visual hallucinations: A critical review. *Parkinsonism Relat Disord* **21**, 683–691 (2015).
3. Goetz, C. G., Vaughan, C. L., Goldman, J. G. & Stebbins, G. T. I finally see what you see: Parkinson's disease visual hallucinations captured with functional neuroimaging. https://doi.org/10.1002/mds.25554 (2014) doi:10.1002/mds.25554.
4. Bhome, R., Thomas, G. E. C., Zarkali, A. & Weil, R. S. Structural and Functional Imaging Correlates of Visual Hallucinations in Parkinson's Disease. *Curr Neurol Neurosci Rep* **23**, 287–299 (2023).
5. Shine, J. M., Halliday, G. M., Naismith, S. L. & Lewis, S. J. G. Visual misperceptions and hallucinations in Parkinson's disease: Dysfunction of attentional control networks? *Movement Disorders* **26**, 2154–2159 (2011).
6. Shine, J. M., O'Callaghan, C., Halliday, G. M. & Lewis, S. J. G. Tricks of the mind: Visual hallucinations as disorders of attention. *Progress in Neurobiology* **116**, 58–65 (2014).
7. Shine, J. M. *et al.* Abnormal connectivity between the default mode and the visual system underlies the manifestation of visual hallucinations in Parkinson's disease: a task-based fMRI study. *NPJ Parkinsons Dis* **1**, 15003 (2015).
8. Weil, R. S. *et al.* Visual dysfunction in Parkinson's disease. *Brain* **139**, 2827–2843 (2016).
9. Fénelon, G., Mahieux, F., Huon, R. & Ziégler, M. Hallucinations in Parkinson's disease: prevalence, phenomenology and risk factors. *Brain* **123 (Pt 4)**, 733–745 (2000).
10. Ravina, B. *et al.* Diagnostic criteria for psychosis in Parkinson's disease: Report of an NINDS, NIMH work group. *Movement Disorders* **22**, 1061–1068 (2007).
11. ffytche, D. H. *et al.* The psychosis spectrum in Parkinson disease. *Nat Rev Neurol* **13**, 81–95 (2017).

12. Lenka, A., Pagonabarraga, J., Pal, P. K., Bejr-Kasem, H. & Kulisvesky, J. Minor hallucinations in Parkinson disease: A subtle symptom with major clinical implications. *Neurology* **93**, 259–266 (2019).
13. Pagonabarraga, J. *et al.* Minor hallucinations occur in drug-naive Parkinson's disease patients, even from the premotor phase. *Movement Disorders* **31**, 45–52 (2016).
14. Bernasconi, F. *et al.* Theta oscillations and minor hallucinations in Parkinson's disease reveal decrease in frontal lobe functions and later cognitive decline. *Nat. Mental Health* **1**, 477–488 (2023).
15. Doé de Maindreville, A., Fénelon, G. & Mahieux, F. Hallucinations in Parkinson's disease: A follow-up study. *Movement Disorders* **20**, 212–217 (2005).
16. Bernasconi, F. *et al.* Robot-induced hallucinations in Parkinson's disease depend on altered sensorimotor processing in fronto-temporal network. *Sci. Transl. Med.* **13**, eabc8362 (2021).
17. Zhong, M. *et al.* Aberrant gray matter volume and functional connectivity in Parkinson's disease with minor hallucination. *Front Aging Neurosci* **14**, 923560 (2022).
18. Bejr-kasem, H. *et al.* Minor hallucinations reflect early gray matter loss and predict subjective cognitive decline in Parkinson's disease. *European Journal of Neurology* **28**, 438–447 (2021).
19. Baik, K. *et al.* Functional Brain Networks of Minor and Well-Structured Major Hallucinations in Parkinson's Disease. *Movement Disorders* **39**, 318–327 (2024).
20. Bejr-kasem, H. *et al.* Disruption of the default mode network and its intrinsic functional connectivity underlies minor hallucinations in Parkinson's disease. *Mov Disord.* **34**, 78–86 (2019).
21. Finn, E. S. *et al.* Functional connectome fingerprinting: identifying individuals using patterns of brain connectivity. *Nat Neurosci* **18**, 1664–1671 (2015).
22. Amico, E. & Goñi, J. The quest for identifiability in human functional connectomes. *Sci Rep* **8**, 8254 (2018).
23. Aarsland, D. *et al.* Parkinson disease-associated cognitive impairment. *Nat Rev Dis Primers* **7**, 47 (2021).
24. Vignando, M. *et al.* Mapping brain structural differences and neuroreceptor correlates in Parkinson's disease visual hallucinations. *Nat Commun* **13**, 519 (2022).

25. Ballanger, B. *et al.* Serotonin 2A Receptors and Visual Hallucinations in Parkinson Disease. *Archives of Neurology* **67**, 416–421 (2010).

26. Firbank, M. J. *et al.* Reduced occipital GABA in Parkinson disease with visual hallucinations. *Neurology* **91**, e675–e685 (2018).

27. Stampacchia, S. *et al.* Fingerprints of brain disease: connectome identifiability in Alzheimer's disease. *Commun Biol* **7**, 1–16 (2024).

28. Troisi Lopez, E. *et al.* Fading of brain network fingerprint in Parkinson's disease predicts motor clinical impairment. *Hum Brain Mapp* **44**, 1239–1250 (2022).

29. Da Silva Castanheira, J. *et al.* The neurophysiological brain-fingerprint of Parkinson's disease. *eBioMedicine* **105**, 105201 (2024).

30. Da Silva Castanheira, J., Wiesman, A. I., Taylor, M. J. & Baillet, S. The Lifespan Evolution of Individualized Neurophysiological Traits. *BioRxiv* https://doi.org/10.1101/2024.11.27.624077 (2024) doi:https://doi.org/10.1101/2024.11.27.624077.

31. Da Silva Castanheira, J., Orozco Perez, H. D., Misic, B. & Baillet, S. Brief segments of neurophysiological activity enable individual differentiation. *Nat Commun* **12**, 5713 (2021).

32. Finn, E. S. & Rosenberg, M. D. Beyond fingerprinting: Choosing predictive connectomes over reliable connectomes. *NeuroImage* **239**, 118254 (2021).

33. Rosenberg, M. D. *et al.* A neuromarker of sustained attention from whole-brain functional connectivity. *Nat Neurosci* **19**, 165–171 (2016).

34. Kaufmann, T. *et al.* Delayed stabilization and individualization in connectome development are related to psychiatric disorders. *Nat Neurosci* **20**, 513–515 (2017).

35. Kaufmann, T. *et al.* Stability of the Brain Functional Connectome Fingerprint in Individuals With Schizophrenia. *JAMA Psychiatry* **75**, 749 (2018).

36. Fu, Z., Liu, J., Salman, M. S., Sui, J. & Calhoun, V. D. Functional connectivity uniqueness and variability? Linkages with cognitive and psychiatric problems in children. *Nat. Mental Health* **1**, 956–970 (2023).

37. Sorrentino, P. *et al.* Clinical connectome fingerprints of cognitive decline. *NeuroImage* **238**, 118253 (2021).

38. Amico, E. & Goñi, J. Mapping hybrid functional-structural connectivity traits in the human connectome. *Network Neuroscience* **2**, 306–322 (2018).

39. Noble, S., Scheinost, D. & Constable, R. T. A decade of test-retest reliability of functional connectivity: A systematic review and meta-analysis. *NeuroImage* **203**, 116157 (2019).

40. Cicchetti, D. V. & Sparrow, S. A. Developing criteria for establishing interrater reliability of specific items: Applications to assessment of adaptive behavior. *American Journal of Mental Deficiency* **86**, 127–137 (1981).

41. Shen, X., Tokoglu, F., Papademetris, X. & Constable, R. T. Groupwise whole-brain parcellation from resting-state fMRI data for network node identification. *NeuroImage* **82**, 403–415 (2013).

42. Markello, R. D. *et al.* neuromaps: structural and functional interpretation of brain maps. *Nat Methods* **19**, 1472–1479 (2022).

43. Zarkali, A. *et al.* Changes in dynamic transitions between integrated and segregated states underlie visual hallucinations in Parkinson's disease. *Commun Biol* **5**, 928 (2022).

44. Huot, P. *et al.* Increased 5-HT2A receptors in the temporal cortex of parkinsonian patients with visual hallucinations. *Movement Disorders* **25**, 1399–1408 (2010).

45. Perry, E. K. & Perry, R. H. Acetylcholine and hallucinations: disease-related compared to drug-induced alterations in human consciousness. *Brain Cogn* **28**, 240–258 (1995).

46. Pasquini, J., Brooks, D. J. & Pavese, N. The Cholinergic Brain in Parkinson's Disease. *Movement Disorders Clinical Practice* **8**, 1012–1026 (2021).

47. Xia, M., Wang, J. & He, Y. BrainNet Viewer: A Network Visualization Tool for Human Brain Connectomics. *PLoS ONE* **8**, e68910 (2013).

48. Goetz, C. G. *et al.* Movement Disorder Society-sponsored revision of the Unified Parkinson's Disease Rating Scale (MDS-UPDRS): scale presentation and clinimetric testing results. *Mov Disord* **23**, 2129–2170 (2008).

49. Fernández de Bobadilla, R. *et al.* Parkinson's disease-cognitive rating scale: Psychometrics for mild cognitive impairment. *Movement Disorders* **28**, 1376–1383 (2013).

50. Bejr-Kasem, H. *et al.* The role of attentional control over interference in minor hallucinations in Parkinson's disease. *Parkinsonism Relat Disord* **102**, 101–107 (2022).

51. Pagonabarraga, J., Bejr-Kasem, H., Martinez-Horta, S. & Kulisevsky, J. Parkinson disease psychosis: from phenomenology to neurobiological mechanisms. *Nat Rev Neurol* **20**, 135–150 (2024).

52. Kim, N. Y. *et al.* Lesions causing hallucinations localize to one common brain network. *Mol Psychiatry* **26**, 1299–1309 (2021).

53. Pagonabarraga, J. *et al.* Neural correlates of minor hallucinations in non-demented patients with Parkinson's disease. *Parkinsonism & Related Disorders* **20**, 290–296 (2014).

54. Moberget, T. & Ivry, R. B. Prediction, Psychosis, and the Cerebellum. *Biol Psychiatry Cogn Neurosci Neuroimaging* **4**, 820–831 (2019).

55. Blanke, O. *et al.* Neurological and robot-controlled induction of an apparition. *Curr Biol* **24**, 2681–2686 (2014).

56. Rajamani, N. *et al.* Deep brain stimulation of symptom-specific networks in Parkinson's disease. *Nat Commun* **15**, 4662 (2024).

57. Postuma, R. B. *et al.* MDS clinical diagnostic criteria for Parkinson's disease. *Movement Disorders* **30**, 1591–1601 (2015).

58. Pini, L., Lista, S., Griffa, A., Allali, G. & Imbimbo, B. P. Can brain network connectivity facilitate the clinical development of disease-modifying anti-Alzheimer drugs? *Brain Communications* **7**, fcae460 (2024).

59. Ramduny, J. & Kelly, C. Connectome-based fingerprinting: reproducibility, precision, and behavioral prediction. *Neuropsychopharmacol.* **50**, 114–123 (2024).

60. Gordon, E. M. *et al.* Precision Functional Mapping of Individual Human Brains. *Neuron* **95**, 791-807.e7 (2017).

61. Frossard, J. & Renaud, O. Permutation Tests for Regression, ANOVA, and Comparison of Signals: The **permuco** Package. *J. Stat. Soft.* **99**, (2021).

62. Albert, L., Potheegadoo, J., Herbelin, B., Bernasconi, F. & Blanke, O. Numerosity estimation of virtual humans as a digital-robotic marker for hallucinations in Parkinson's disease. *Nat Commun* **15**, 1905 (2024).

63. Bernasconi, F. *et al.* Neuroscience robotics for controlled induction and real-time assessment of hallucinations. *Nat Protoc* 1–24 (2022) doi:10.1038/s41596-022-00737-z.

Detailed response to reviewers

We thank the Editor and Reviewers for considering the revised work and their careful evaluation. We indicate below how we addressed the remaining concerns.

Reviewer #1 (Remarks to the Author):

Minor comment 1

The sentence "whereas da Silva Castanheira et al. [29] replicated and extended these findings by showing that identifiability remained high in PD relative to healthy controls only when focusing on arrhythmic activity, predicting symptoms laterality." Should read "when focusing on rhythmic activity." The findings of da Silva Castanheira et al. (2024) demonstrated that PD patients remained differentiable from rhythmic brain activity, and were more challenging to differentiate from arrhythmic brain activity.

We thank Reviewer 1 for catching this. The sentence now correctly refers to *rhythmic* activity.

The manuscript has been corrected accordingly (page 3):

[…] whereas da Silva Castanheira et al.[1] replicated and extended these findings by showing that identifiability remained high in PD relative to healthy controls only when focusing on rhythmic activity, predicting the laterality of the symptoms.

Minor comment 2

I would refrain from describing brain-fingerprinting as an identification process, it much better described as differentiation. Identification comes with specific connotations and may misguide readers, specially those who are not familiar with the field. I would therefore rephrase the paper accordingly to remain consistent with the rest of the field and avoid confusion.

e.g., "identification of individuals from a population" and "reported decreased identifiability in PD vs. healthy controls predicting clinical motor impairment."

We thank Reviewer 1 for this comment. While brain fingerprinting can also be framed in terms of differentiation, the terms *identification* and *identifiability* are well-established in the literature that first introduced this approach [2,3] and continue to be used in more recent studies (e.g., [4,5]). In the present paper, "identification" specifically refers to the task of correctly matching individuals to their connectomes – that is, demonstrating higher within-subject similarity than between-subject similarity – consistent with this prior literature and established methodology. To clarify this and avoid any

potential confusion, we have revised the text to specify that our use of "identification" refers to "*connectome-based identification,*" which leverages individual differences.

The introduction (p. 3) now reads as follows *(changes in blue underlined):*

Functional connectivity patterns estimated from resting-state functional magnetic resonance imaging (fMRI) or magnetoencephalography (MEG) data – referred to as functional connectomes (FCs) – are unique to each individual [2,3,6]. Accordingly, these have been called *brain-fingerprints* as they allow for the identification of individuals from a population; i.e., the correct matching of an individual to their own functional connectome, leveraging individual differences across individuals. In this manuscript, we therefore refer to this property of functional connectomes as brain-fingerprinting or identifiability, used interchangeably. These unique features of functional connectomes have been linked to cognitive traits [7,8] , and are altered in psychiatric [9–11] as well as neurodegenerative diseases, including Alzheimer's Disease [12,4] and PD [1,13].

Minor comment 3

Brain renders in Figure 3C: I am still a bit confused about the interpretation of the results presented in Figure 3C. From what I understand, these maps correspond to the strength of each FC edge identified by the ΔICC approach, and the strength (colour) of the edges is how much each individual 'expresses' that connection. If that is the case, then each participant should have the nodes in the same location across renders, and the only thing that should differ between renders is the colour across edges between participants. But this is not the case. Could the authors clarify this?

Yes. We use ΔICC to select features (edges) with a high difference between the two groups in term of ICC. We then apply this selection to each individual and split them into positive (functional connectivity or Pearson's $r > 0$) and negative (functional connectivity or Pearson's $r < 0$) connections, in order to avoid cancellation effects when computing network strengths.

Given that this sign-based selection is performed at the individual level, the subset of retained edges (and therefore the nodes participating in those edges) can differ slightly across participants. For this reason, not all individuals show nodes in exactly the same locations across renders. However, as shown in Figure 3C, there is substantial overlap in the spatial distribution of nodes across subjects.

We have now clarified this point in the legend of figure 3 *(changes in blue underlined):*

Figure 3. Functional connections with high fingerprint associate with clinical scores. A) Brain renders showing the top 0.1% ΔICC edges for each group. The top ΔICC matrices were used as masks to isolate the functional connections that

maximise differentiability in fingerprint between the two groups in each individual functional connectome. B) Large scatterplot: linear regression models where clinical scores were significantly associated with functional connectivity in the top ΔICC edges (Clinical score ~ $FC_{\text{top }\Delta ICC\text{ group}}$ + Age + YoE + Disease duration). Small scatterplot: correlation between residuals of the clinical score and functional connectivity to show directionality of the association. C) Patient's specific brain renders showing the functional connections with high fingerprint associated with clinical score. Each render displays, for every participant, the FC strength of the top 0.1% ΔICC edges identified at the group level (Fig. 3A). The group-derived ΔICC mask was applied to each subject's functional connectome, and the FC values within these edges were extracted (and averaged for the score in Fig. 3B). Positive and negative ΔICC edges were treated separately to avoid cancellation effects when computing network strength. As this separation is performed at the individual level, the subset of expressed edges (and corresponding nodes) may vary slightly across participants, although their spatial distribution largely overlaps. The colour intensity reflects the subject-specific expression of the minor hallucination-related fingerprint.

Reviewer #2 (Remarks to the Author):

The authors have made a great effort to respond to the points made. Their responses are satisfactory, and the resultant modifications have enriched the manuscript hallucinatory phenomena t.

We thank Reviewer 2 for the positive evaluation of our revisions.

Comment 1

I still find it somewhat counter-productive that they have based this whole very complex analysis on a biniary classification based on a single question of the MDS/UPDRS I and a "semi-structured" interview. There are a number of scales that assess in a structured fashion the intensity and frequency of hallucinatory phenomena that could have been used. In any case, the results show that even this rather crude classification works, so perhaps this is a mute point; it would be important in future work for the group if such tools were utilized.

We thank the reviewer for raising this point and we fully agree that a more detailed and structured assessment of hallucinatory phenomena would be desirable. Indeed, much of the research of our group is dedicated to improved methods to describe and quantify hallucinations. This also includes developing experimental paradigms to systematically induce hallucination-like experiences in the laboratory in patients [14,15] and to quantify them using behavioral measures [16].

For the dataset used in the present study, more fine-grained clinical and experimental instruments were unavailable, and we therefore relied on the binary classification derived from the standard MDS-UPDRS Part I that were combined with a detailed semi-structured interview. This binary stratification has been employed in several previously published work when studying patients with MH (e.g., see [14,17–19]). We also note that a detailed interview is still the clinical gold-standard in the clinic when investigating hallucinations, including MH. The current results extend this prior work by demonstrating that this clinically defined distinction maps onto robust individual-specific brain-level connectivity differences. Future studies will build on these findings by further analyzing potential additional hallucination-related brain differences. We hope that these will also be based on experimental paradigms and behavioral measures related to hallucinations.

Comment 2

In point 2, it should rather be "short" than "mild-to-moderate" disease duration.

Thanks!

Page 10 now reads as follows:

We note, however, that our cohort included patients with a relatively short disease duration (mean ± SD: 5.2 ± 3.8 and 4 ± 2 years for the two groups).

Reviewer #3 (Remarks to the Author):

The authors addressed part of my concerns regarding sample size and length of MRI scans. It is helpful to include controls in this revision.

We appreciate the positive feedback of Reviewer 3 on the revisions.

Comment 1

I am not convinced by their rebuttal regarding statistical concerns. There are no group difference between MH and nH patients in terms of primary fingerprint indices (ISelf, IOthers). ICC matrices collapse individual information into a group-wise measure. Therefore, it is merely a descriptive measure for each group. Spatial specificity (network) comparisons at group level are merely secondary analysis of ICC matrices property between groups. It cannot infer between subject group differences.

As reported in the revised version of the manuscript, there were no significant differences in the whole-brain fingerprint indices (ISelf, IOthers). We consider this a meaningful and positive finding, as it demonstrates that identifiability is preserved in PD (as shown by the comparison against healthy controls) and it is not affected by hallucinations. This was the foundation of our subsequent ICC-based analyses, in which we identified which features drive this identifiability. We note that we never claim in the manuscript that ICC directly infers *between-subject* differences. ICC quantifies how much each functional connection supports fingerprinting. Specifically, a high ICC indicates that the connection is highly stable within individuals while varying across individuals, i.e., it contributes to identifiability. Therefore, using this metric, we quantify how connections drive identifiability for each group, separately. We then tested for differences in the distribution of MH-related ICC edges across functional networks by first computing the percentage of edges per network exceeding the 97th percentile, and assessed differences using a binomial test, with FDR correction across network comparisons. This approach is done to address differences in spatial specificity across the groups. We have carefully rechecked the entire manuscript and make no statements that ICC directly infers *between-subject* differences. Moreover, we added explicit statements in the discussion about this.

Discussion (Page 9) *(changes in blue underlined):*

First, we observed that identifiability is preserved in the presence of PD, comparable to healthy controls (HC), and irrespective of our patients' hallucination status, with no group differences between PD-MH, PD-nH, and HC in primary fingerprint indices (*ISelf, IOthers*) (Fig. 1).

Please also see our response to your Comment 2 below, based on which we have revised the methods part on ICC, clarifying the way it is calculated, and were we explicitly state:

Methods (Page 19) (changes in blue underlined):

We note that ICC does not directly infer *between-subject* differences

I would suggest that author clearly state the negative findings in the abstract, tune down overall value of fingerprint, and focus on ICC matrics and its value in detecting network differences and correlating with clinical scores.

In the abstract, we now explicitly state the lack of differences in identifiability between the two groups, which – as argued above – constitutes a positive finding, as it supports the hypothesis that fingerprints are maintained irrespective of hallucinations. We then shift the focus to the ICC-based results, highlighting their relevance to the suggested clinical associations. Given the high-level, concise format of a *Nature*-style abstract, it was considered more appropriate to emphasize the study's main take-home message rather than to detail the metric itself. We hope this addresses the Reviewers' concern.

The abstract now reads as follows. (changes in blue underlined, parts addressing reviewers concern in blue):

Abstract

Individual variability in connectome organization offers a unique framework for capturing patient-specific alterations. Minor hallucinations (MH) affect up to 40% of Parkinson's disease (PD) patients and are early indicators of cognitive decline, hence crucial for early intervention. Unlike previous group-level studies, connectome-based brain fingerprinting enables to capture individualized neural changes. Applying this approach to PD patients with and without MH using resting-state fMRI, we show that each patient exhibited unique brain fingerprint, albeit irrespective of MH status. These fingerprint features were associated with clinically relevant measures such as motor symptom severity and cognitive performance. MH-patients showed a loss of subject-specific features in brain networks linked to cognitive health, while cerebellar-to-cortex connectivity – typically less distinctive – became more prominent, emphasizing the role of somatosensory and motor integration in MH pathogenesis. Moreover, MH-related fingerprint regions mapped onto cortical territories with lower densities of neurotransmitter systems implicated in hallucinations. Overall, our fully data-driven findings reveal a distinct, patient-specific connectomic signature that differentiates PD patients with MH.

Comment 2

Despite lengthy description of ICC, it is not quite clear how it is calculated. Please revise and make it clear.

We have revised the methods section to more clearly and concisely describe the edge-wise computation of the ICC, bringing additional clarification to the procedure.

Page 19 (Methods) now reads as follows:

Spatial specificity of brain fingerprint: edge-wise intra-class correlation

Spatial specificity of FC fingerprints was derived using edgewise intra-class correlation (ICC), computed separately for each functional connection (edge) and for each group. For any given edge, ICC was calculated across subjects using test and retest FC values as repeated measurements, according to a one-way random-effects model (ICC(1,1))[20] (cf. Fig. 2A):

$$ICC = \frac{MS_R - MS_W}{MS_R + (k-1)MS_W},$$

where MS_R is the mean square between subjects (rows); MS_W the mean square for residual sources of variance (within-subjects variability across sessions); k the number of sessions (here, test and retest). A high ICC for a given edge indicates that a larger proportion of the variance across test and retest is due to differences between the subjects, rather than within-subjects differences between test and retest or random noise. Conversely, a low ICC indicates greater within-subjects variability than across subjects. Thus, edges with high ICC values represent connections that are both reliable within individuals and discriminative across subjects; i.e., driving identifiability, consistent with the concept of functional connectivity "fingerprint" [21,22]. We note that ICC does not directly infer *between-subject* differences.

The ICC has been commonly used in the functional connectivity literature to assess reliability [21,23–26]. A few strengths of the ICC include: 1) the ability to assess absolute agreement in repeated measurements of an object (unlike, for example, Pearson's correlation, wherein variables are scaled and cantered separately), 2) the ability to explicitly model multiple known sources of variability (e.g., scanner, brain response, head motion), and 3) comparing within and between variability across the objects of measurement. Depending on whether and how sources of error (or "facets"; e.g., scanner) may be specified, one of three ICC forms may be used [27]. [...]

Comment 3

The spatial correlation between ICC maps and neurotransmitter maps is interesting. In method part, it states that group-specific ICC matrix was correlated with neurotransmitter maps. But only MH results are present in results section. Are there any significant correlations between ICC matrix from nH patients with neurotransmitter maps? Please also discuss how the correlations

between spatial patterns from MH patients (the fingerprint) and normal controls (neurotransmitters) are meaningful to infer the reached conclusion.

We thank Reviewer 3 for appreciating this analysis. Reviewer 3 is correct that in the 1st round of revision, we stated in the methods section that *group-specific* ΔICC maps were derived from the nodal strength of the group-specific ICC matrices. However, this was an error from the originally submitted version that was not deleted. It does not reflect the current analyses. We have now updated the manuscript accordingly. We thank the reviewer for noticing this.

Concerning the neurotransmitter maps, we note that following the suggestion from Reviewer 1 during the first round of revisions, we correlated neurotransmitter maps with a single ΔICC map without splitting by group. This approach, as proposed by Reviewer 1, provides a more intuitive interpretation in which positive ΔICC values directly correspond to hallucination-signature regions.

Accordingly, the current results indicate that connections showing a stronger hallucination-specific brain fingerprint are associated with significantly lower cortical density of 5-HT2A serotonin (FDR-p = 0.028), GABAa (FDR-p = 0.045), and M1 acetylcholine (FDR-p = 0.016) receptors. Conversely, in non-hallucinating patients, regions with higher fingerprint scores have higher cortical density of the same receptors.

The Method section now reads as follows (p.22) ~~*(removed text)*~~

In these analyses, we looked at which neurotransmitter systems showed topographical correspondence with the brain fingerprint topography (Fig. 4). We estimated the spatial correlation between the ~~group-wise~~ ΔICC map derived from nodal strength of the ~~group-specific~~ ΔICC matrix and each of the normative atlas maps of the neurotransmitters' receptors of dopamine, serotonin, GABA and acetylcholine given their implication in the neurobiology of hallucinations. Normative neurotransmitter density data are available from neuromaps (https://github.com/netneurolab/neuromaps) [29].

Minor comment 1:

A minor issue: please provide parallel MRI acquisition parameters for patients and controls along with scan time duration of each group, scanner software version.

We now provide the scan time duration for each group. Parallel MRI acquisition parameters and scanner software version are provided for Newcastle dataset, but these parameters were not available for the Barcelona data set.

Page 16 now reads as follows:

Image acquisition parameters

PD patients: MRI scans were acquired with a 3T Philips Achieva at the Hospital de la Santa Creu i Sant Pau in Barcelona. T1 weighted scans were obtained using a MPRAGE sequence (TR = 500 ms, TE = 50 ms, flip angle = 8°, field of view = 23 cm with in-plane resolution of 256 × 256, slice thickness = 1mm). Resting-state fMRI images were collected using an echo-planar sequence (TR = 2000 ms, TE = 30 ms, flip angle = 78°, field of view = 240 mm, slice thickness = 3 mm, 180 volumes). HC: MRI scans were acquired at Newcastle University with a 3T Philips Intera Achieva scanner (software release R5.1.7). T1 weighted scans were obtained using a MPRAGE sequence (TR = 8.3 ms, TE = 4.6 ms, flip angle = 8°, in-plane resolution = 1.0 × 1.0 mm2, slice thickness = 1.0 mm). Resting-state fMRI images were collected using a gradient echo echo-planar imaging sequence with 33 contiguous axial slices (TR = 2072 ms, TE = 30 ms, field of view = 192 × 192 mm^2, in-plane resolution = 3.0 × 3.0 mm, slice thickness = 3 mm with 1.0 mm gap, 290 volumes). Parallel imaging was performed using SENSE with an acceleration factor of 2. To mitigate the confounding effect of unequal time-series length on fingerprinting metrics and to match the temporal resolution of the patients' cohort, we limited the analyses to the first 180 of the 290 acquired volumes. This corresponded to 6 and 6.2 minutes of data for PD patients and HC, respectively.

References

1. Da Silva Castanheira, J. *et al.* The neurophysiological brain-fingerprint of Parkinson's disease. *eBioMedicine* **105**, 105201 (2024).
2. Finn, E. S. *et al.* Functional connectome fingerprinting: identifying individuals using patterns of brain connectivity. *Nat. Neurosci.* **18**, 1664–1671 (2015).
3. Amico, E. & Goñi, J. The quest for identifiability in human functional connectomes. *Sci. Rep.* **8**, 8254 (2018).
4. Stampacchia, S. *et al.* Fingerprints of brain disease: connectome identifiability in Alzheimer's disease. *Commun. Biol.* **7**, 1–16 (2024).
5. Sareen, E. *et al.* Exploring MEG brain fingerprints: Evaluation, pitfalls, and interpretations. *NeuroImage* **240**, 118331 (2021).

6. Da Silva Castanheira, J., Orozco Perez, H. D., Misic, B. & Baillet, S. Brief segments of neurophysiological activity enable individual differentiation. *Nat. Commun.* **12**, 5713 (2021).
7. Finn, E. S. & Rosenberg, M. D. Beyond fingerprinting: Choosing predictive connectomes over reliable connectomes. *NeuroImage* **239**, 118254 (2021).
8. Rosenberg, M. D. *et al.* A neuromarker of sustained attention from whole-brain functional connectivity. *Nat. Neurosci.* **19**, 165–171 (2016).
9. Kaufmann, T. *et al.* Delayed stabilization and individualization in connectome development are related to psychiatric disorders. *Nat. Neurosci.* **20**, 513–515 (2017).
10. Kaufmann, T. *et al.* Stability of the Brain Functional Connectome Fingerprint in Individuals With Schizophrenia. *JAMA Psychiatry* **75**, 749 (2018).
11. Fu, Z., Liu, J., Salman, M. S., Sui, J. & Calhoun, V. D. Functional connectivity uniqueness and variability? Linkages with cognitive and psychiatric problems in children. *Nat. Ment. Health* **1**, 956–970 (2023).
12. Sorrentino, P. *et al.* Clinical connectome fingerprints of cognitive decline. *NeuroImage* **238**, 118253 (2021).
13. Troisi Lopez, E. *et al.* Fading of brain network fingerprint in Parkinson's disease predicts motor clinical impairment. *Hum. Brain Mapp.* **44**, 1239–1250 (2022).
14. Bernasconi, F. *et al.* Robot-induced hallucinations in Parkinson's disease depend on altered sensorimotor processing in fronto-temporal network. *Sci. Transl. Med.* **13**, eabc8362 (2021).
15. Bernasconi, F. *et al.* Neuroscience robotics for controlled induction and real-time assessment of hallucinations. *Nat. Protoc.* 1–24 (2022) doi:10.1038/s41596-022-00737-z.
16. Albert, L., Potheegadoo, J., Herbelin, B., Bernasconi, F. & Blanke, O. Numerosity estimation of virtual humans as a digital-robotic marker for hallucinations in Parkinson's disease. *Nat. Commun.* **15**, 1905 (2024).
17. Bernasconi, F. *et al.* Theta oscillations and minor hallucinations in Parkinson's disease reveal decrease in frontal lobe functions and later cognitive decline. *Nat. Ment. Health* **1**, 477–488 (2023).
18. Bejr-Kasem, H. *et al.* The role of attentional control over interference in minor hallucinations in Parkinson's disease. *Parkinsonism Relat. Disord.* **102**, 101–107 (2022).

19. Bejr-kasem, H. *et al.* Disruption of the default mode network and its intrinsic functional connectivity underlies minor hallucinations in Parkinson's disease. *Mov. Disord.* **34**, 78–86 (2019).

20. McGraw, K. O. & Wong, S. P. Forming Inferences About Some Intraclass Correlation Coefficients. *Psychological Methods* 17 (1996) doi:1082-989X/96/S3.00.

21. Amico, E. & Goñi, J. Mapping hybrid functional-structural connectivity traits in the human connectome. *Netw. Neurosci.* **2**, 306–322 (2018).

22. Van De Ville, D., Farouj, Y., Preti, M. G., Liégeois, R. & Amico, E. When *Makes You Unique: Temporality of the Human Brain Fingerprint*. http://biorxiv.org/lookup/doi/10.1101/2021.03.24.436733 (2021) doi:10.1101/2021.03.24.436733.

23. Birn, R. M. *et al.* The effect of scan length on the reliability of resting-state fMRI connectivity estimates. *NeuroImage* **83**, 550–558 (2013).

24. Somandepalli, K. *et al.* Short-term test–retest reliability of resting state fMRI metrics in children with and without attention-deficit/hyperactivity disorder. *Dev. Cogn. Neurosci.* **15**, 83–93 (2015).

25. Shehzad, Z. *et al.* The resting brain: unconstrained yet reliable. *Cereb. Cortex N. Y. N 1991* **19**, 2209–2229 (2009).

26. Shah, L. M., Cramer, J. A., Ferguson, M. A., Birn, R. M. & Anderson, J. S. Reliability and reproducibility of individual differences in functional connectivity acquired during task and resting state. *Brain Behav.* **6**, e00456 (2016).

27. Noble, S., Scheinost, D. & Constable, R. T. A decade of test-retest reliability of functional connectivity: A systematic review and meta-analysis. *NeuroImage* **203**, 116157 (2019).

28. Cicchetti, D. V. & Sparrow, S. A. Developing criteria for establishing interrater reliability of specific items: Applications to assessment of adaptive behavior. *Am. J. Ment. Defic.* **86**, 127–137 (1981).

29. Markello, R. D. *et al.* neuromaps: structural and functional interpretation of brain maps. *Nat. Methods* **19**, 1472–1479 (2022).
